# Supplementary material for: Diagnostic value of ASVS for insulinoma localization: A systematic review and meta-analysis
Source: PLoS One. 2019 Nov 19;14(11):e0224928. doi: 10.1371/journal.pone.0224928 (PMC6863549; doi:10.1371/journal.pone.0224928)
Supplement: S2 File — (ZIP) [file pone.0224928.s002.zip › included studies/Arterial Calcium Stimulation with Hepatic Venous Sampling in.pdf]

Hindawi Publishing Corporation
International Journal of Endocrinology
Volume 2016, Article ID 4581094, 6 pages
http://dx.doi.org/10.1155/2016/4581094

Research Article

Arterial Calcium Stimulation with Hepatic Venous Sampling in the Localization Diagnosis of Endogenous Hyperinsulinism

Paloma Moreno-Moreno,[1,2] María Rosa Alhambra-Expósito,[1,2] Aura Dulcinea Herrera-Martínez,[1,2] Rafel Palomares-Ortega,[2] Luis Zurera-Tendero,[3] Juan José Espejo Herrero,[3] and María Angeles Gálvez-Moreno[1,2]

[1] *Maimonides Institute for Biomedical Research of Cordoba (IMIBIC), Córdoba, Spain*
[2] *Endocrinology and Nutrition Service, Reina Sofia University Hospital, Córdoba, Spain*
[3] *Radiology Department, Reina Sofia University Hospital, Córdoba, Spain*

Correspondence should be addressed to Aura Dulcinea Herrera-Martínez; aurita.dhm@gmail.com

Received 21 February 2016; Revised 25 June 2016; Accepted 4 August 2016

Academic Editor: Thomas J. Fahey

Copyright © 2016 Paloma Moreno-Moreno et al. This is an open access article distributed under the Creative Commons Attribution License, which permits unrestricted use, distribution, and reproduction in any medium, provided the original work is properly cited.

Objective. The aim of this study was to assess the utility of arterial calcium stimulation with hepatic venous sampling (ASVS) in the localization diagnosis of endogenous hyperinsulinism. *Patients and Methods*. A retrospective descriptive study was performed including patients with endogenous hyperinsulinism who underwent ASVS. The histopathological diagnosis in patients who underwent a surgical procedure was used as the reference for the statistical study of the accuracy of this technique. *Results*. 30 patients were included with endogenous hyperinsulinism and nonconclusive imaging diagnosis was included. ASVS was performed in all cases. Surgery was performed in 20 cases. Insulinoma was removed in 19 patients; the location of all cases was detected in the ASVS. All cases of endogenous hyperinsulinism had a positive result for the ASVS, with this association being statistically significant ($\chi^2 = 15.771$; $p < 0.001$). A good and statistically significant agreement was obtained between histopathologic diagnosis and ASVS results ($K = 0.518$, $p < 0.001$). *Conclusions*. ASVS is a useful procedure in the localization diagnosis of endogenous hyperinsulinism undetected by other imaging tests. This technique allows the localization of intrapancreatic insulinomas and represents useful tool for the diagnosis and surgical management of these tumors.

1. Introduction

Hypoglycemia is manifested by the appearance of Whipple's triad: hypoglycemia (plasma glucose < 50 mg/dL), neuroglycopenic symptoms, and prompt relief of symptoms following the administration of glucose. The most common causes of hypoglycemia in nondiabetic healthy adults are factitious hypoglycemia and endogenous hyperinsulinism [1, 2].

The diagnosis of hyperinsulinemic hypoglycemia is established by demonstrating inappropriately high serum insulin concentrations during fasting hypoglycemia, specifically a prolonged supervised fast. Endogenous hyperinsulinism includes insulinoma, nesidioblastosis, and hyperinsulinemia of autoimmune origin. Hypoglycemia due to hyperinsulinism of autoimmune origin is a very rare disorder, and nesidioblastosis is a rare cause of hypoglycemia in adults. Insulinoma is after factitious hypoglycemia the most common cause of hypoglycemia in apparently healthy adults.

Insulinomas are the most common functioning endocrine neoplasm of the pancreas and the most common cause of hypoglycemia in apparently healthy adults once factitious hypoglycemia has been excluded. These tumors represent 1%-2% of all pancreatic neoplasms, with an estimated incidence of 4 per million population per year; they can occur at any age, with a highest incidence between the third and sixth decade and an equal gender distribution; 90% are benign, solitary, with a maximum diameter less than two centimeters; only 5–10% of cases are part of multiple endocrine neoplasia

type 1 (MEN-1) or have malignant behavior. Extrapancreatic insulinomas are extremely rare and most commonly found in the duodenal wall [1–4].

Diagnosis and treatment of these tumors are curative in most cases; preoperative localization improves the chance of cure and reduces the likelihood of complications [5, 6]. Erroneous preoperative localization of insulinoma is estimated at 75% of cases depending on the diagnosis method [7]. Preoperative localization methods include transabdominal ultrasonography (US), computed tomography (CT), magnetic resonance imaging (MRI) [8], endoscopic ultrasonography (EUS) [9, 10], and intra-arterial calcium stimulation test with hepatic venous sampling (ASVS) [2, 5, 11]. Noninvasive techniques globally allow the localization of less than 80% of insulinomas. The sensitivity of US in the localization of insulinomas is less than 64% [9]; CT has reported sensitivity in the range 33–64% and MRI 40–90% [10]. In current practice, CT is currently accepted as the first-line technique for the localization of insulinomas while MRI is considered as a second-line one.

Invasive techniques, such as EUS and ASVS, have been shown to be highly accurate in the preoperative localization of insulinomas compared to noninvasive localization techniques. EUS is currently the test of choice in most centers, with reported detection rates ranging from 86.6% to 92.3% [12]. Combination of preoperative multislice CT with EUS allows the location of insulinoma with 100% sensitivity and 95% specificity; other invasive techniques are recommended only if these are negative [13]. However, the tumor localization by EUS depends on the examiner's experience and the location and the size of the insulinoma; some limitations have been reported in tumors with a different localization than the head of the pancreas [14].

The selective venous sampling after percutaneous transhepatic puncture is an invasive technique with greater sensitivity (75–100%) and specificity (94–100%) [2, 15]. Doppman et al. [16, 17] introduced ASVS as an alternative method with similar results and less morbidity. The use of ASVS allows for a more accurate surgical approach and can minimize the likelihood of reoperation [18].

After the identification of an insulinoma, surgery is indicated in all localized tumors if the patient is able to support the surgery. The surgical technique depends mainly on the location and size of insulinoma. Conservative surgery techniques as enucleation, partial pancreatectomy, or middle pancreatectomy have the advantage of preserving pancreatic parenchyma as much as possible, thereby reducing the risk of late exocrine/endocrine insufficiency.

The aim of this study is to evaluate the usefulness of ASVS in the localization diagnosis of endogenous hyperinsulinism: insulinomas and nesidioblastosis.

2. Patients and Methods

We carried out a retrospective review as a single-center study in Reina Sofia University Hospital of Córdoba (Spain).

2.1. Subjects. Inclusion criteria were all patients with clinical and biochemical diagnosis felt to be consistent with endogenous hyperinsulinism. In all the analyzed cases, the noninvasive localization techniques (US, abdominal CT, or MRI) were inconclusive. For the analysis of localization the patient group was restricted to those who had been treated surgically, in whom subsequent histological analysis confirmed insulinoma or nesidioblastosis. We reviewed data from all eligible patients presenting to our center during the period 1997–2011.

2.2. Biochemical Diagnosis. Clinical history, demographic characteristics, and relevant biochemical investigations were reviewed. Symptomatic hypoglycemia (glucose ≤ 50 mg/dL), elevated plasma insulin (>6 mU/L), elevated C-peptide levels (>0.2 pmol/mL), and negative serum detection of sulfonylureas were confirmed by means of prolonged supervised fast test.

2.3. Imaging Techniques. Noninvasive localization methods included CT and MRI. CT was performed using a fine slice multidetector scanner; when possible, intravenous (IV) contrast was administrated and a triple-phase CT scan of the pancreas was obtained. Octreoscan was also performed. Results were discussed in multidisciplinary sessions and in some cases EUS was performed.

2.4. Arterial Calcium Stimulation with Hepatic Venous Sampling (ASVS). Under conscious sedation, 5 Fr catheters were inserted into both the right femoral artery and vein using the Seldinger technique. Under fluoroscopic guidance, the venous catheter was positioned in the right hepatic vein for blood sampling. Standard pancreatic arteriography was performed following selective catheterization of the celiac, gastroduodenal, splenic, and superior mesenteric arteries. The gastroduodenal artery supplies the top of the head and neck of the pancreas; the superior mesenteric artery supplies the lower part of the head and uncinate process; the proximal splenic artery irrigates the body and tail of the pancreas; and the hepatic artery itself detects possible liver metastases [13, 14, 19, 20]. At the same time, gastroduodenal, superior mesenteric, hepatic proper, and proximal splenic arteries were selectively catheterized for performing selective angiography and the injection of calcium gluconate.

After introducing the catheter in the right hepatic vein, selective arteriography was performed for confirming the location of the catheter; it was also useful for analyzing vascular irregularities or for diagnosing a vascularized pancreatic tumor.

After each selective arteriogram, 10% calcium gluconate (Calcium-Sandoz, 5 mL ampoules diluted to a volume of 5 mL with normal saline) was injected into the selected artery at a dose of 0.025 mEq/Kg body weight. Five-milliliter blood samples from the hepatic vein were obtained 10 minutes before the calcium injection and 30, 60, 90, and 120 seconds after. Samples were kept on ice, until they could be centrifuged. The resulting serum was assayed for insulin microparticle enzyme immunoassay (AxSYM Insulin, Abbott, USA). An increase in insulin concentration from baseline to twofold peak or greater constituted a positive response. Depending on the selective artery positive response, the tumor localization

could be predicted. When a positive response was observed in more than one artery, the territory with the greatest insulin rise was used to predict regionalization.

2.5. Statistical Analysis. Descriptive statistical analysis was performed. *Chi-squared* test with Yates' correction for contingency tables was used for comparing proportions; *Cohen's kappa coefficient* determined the measure of interrate agreement between two observations. The quality of diagnostic tests was determined, including sensitivity, specificity, positive predictive value, negative predictive value, positive likelihood ratio, and negative likelihood ratio.

3. Results

ASVS was performed in 30 patients. 83.3% of them were female, with a mean age of 40.56 ± 12.59. 16.70% were male; their mean age was 32.40 ± 15.39.

US did not detect any pancreatic anomaly: conventional abdominal CT and abdominal MRI suggested a possible image of insulinoma in only 6 cases. EUS was performed only in 4 cases being positive in only 1 of them. Arteriography was 10 performed in the same way as ASVS, with a positive or suspicious result in 7 cases. Intraoperative US was positive in 8 of 19 cases. The ASVS was positive in 23 cases (76.70%) and negative in 7 patients (see Figure 1; specific results for each patient are available in Table 1).

In our group, 20 patients underwent surgery; in 13 cases, the surgical resection was based only on the ASVS result; in 16 patients a neuroendocrine tumor was removed ($\chi^2 = 16.02$, $p \leq 0.01$). The tumor was located in the body tail of the pancreas in 9 cases (tumor mean size: 1.31 cm; range: 0.20–3.50 cm); only in one case, tumor was found in the head body, and the remaining 6 cases' tumor was located in the head of the pancreas (mean tumor size: 1.37 cm range: 0.80–2.00 cm).

In other patients with positive ASVS, histological techniques diagnosed nesidioblastosis ($n = 3$) and islets hyperplasia ($n = 1$). After surgery, hypoglycemia disappeared in all patients.

Table 2 shows the rates of assessment of CT, MRI, pancreatic angiography, intraoperative US, and ASVS in the studied patients. ASVS showed a sensitivity of 100% and specificity of 20% to detect insulinoma. When patients who had nesidioblastosis were included in the statistical analysis, its sensitivity was 100% and specificity increased to 50%. The same table lists the positive predictive value, negative predictive value, the positive likelihood ratio, and negative likelihood ratio corresponding to each method for the localization of insulinoma and nesidioblastosis.

4. Discussion

Insulinoma is the most frequent neuroendocrine pancreatic tumor. In our report, this tumor was identified in 80% of the studied cases, with a curative rate of 100% after its removal. Owing to the unsatisfactory effect of long-term medical management, surgical resection is considered the treatment of choice in patients with organic hyperinsulinism, especially in nonmetastatic tumors. Surgical techniques depend on tumor location, being curative in most cases. However, in cases of multiple small adenomas or diffused pancreatic injury (nesidioblastosis and beta cell hyperplasia), surgery is not curative in 4–20% of cases [21].

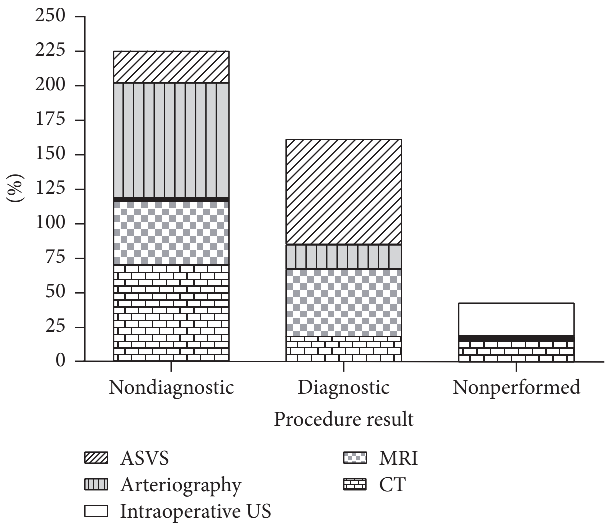

Figure 1: Diagnostic tests for the detection of insulinoma.

Once biochemical diagnosis has been established, preoperative tumor localization is important for treatment decisions, especially for the organization of a conservative surgical procedure, reducing the time of surgery and the surgical morbidity and mortality rate [11]. Usually, a combination of diagnostic techniques is required; available procedures have a sensitivity ranging between 35 and 63% [19, 22] depending on the study series. For this reason, it is important to make a good selection of imaging studies that could offer a reliable location with a high rate of efficiency and, if possible, with a decrease in costs.

ASVS is not only a localization technique but also a diagnostic test for endogenous hyperinsulinism, avoiding unnecessary interventions in cases with negative or inconclusive results. It represents a diagnosis option that may greatly influence the surgical approach for promoting conservative surgery.

In our study, the imaging technique which showed the highest sensitivity (35.71%) was the conventional CT. EUS has had poor results, probably due to a lack of experience, the small tumor size (1.34±0.89 cm), and the insulinoma location (56.25% in body tail). ASVS had the highest quality indices (sensitivity: 100%, specificity: 20%) confirmed by histological analysis ($K = 0.479$, $p < 0.001$). In our series, this procedure also allowed the diagnosis of three cases of nesidioblastosis, showing a rise in insulin secretion in several pancreatic arteries, especially in the head of the pancreas where the most islet mass is located.

Previous studies have reported that conventional location techniques mainly detected tumors larger than 2 cm; intraoperative US and multislice CT progressively demonstrate increased sensitivity to detect the existence of an insulinoma [2, 5, 13, 23]. The EUS is the procedure of choice recommended for some authors [23]; it allows diagnosis of

Table 1: Patients and diagnosis tests for the detection of insulinoma.

Patient number	CT scan	MRI	Intraoperative US	Arteriography	ASVS	Surgery	Final diagnosis
1	ND	ND	ND	ND	P	DPC	I
2	ND	NP	NP	ND	P	DPC	NB
3	ND	NP	NP	ND	P	TP	I
4	NP	ND	NP	ND	N	TP	NB
5	ND	NP	NP	ND	N	NP	NP
6	ND	NP	NP	ND	P	TP	I
7	ND	NP	ND	ND	P	DPC	I
8	D	NP	NP	ND	P	TP	I
9	NP	ND	NP	ND	P	TP	I
10	D	ND	NP	D	P	TE	I
11	D	ND	NP	D	P	DPC	I
12	ND	NP	NP	ND	N	NP	NP
13	ND	NP	NP	ND	P	TP	NB
14	ND	NP	NP	ND	P	DPC	I
15	ND	ND	NP	D	P	TE	I
16	D	NP	NP	ND	P	TE	I
17	NP	ND	NP	ND	P	TP	NB
18	NP	D	NP	D	P	TP	I
19	ND	ND	NP	ND	P	TP	I
20	ND	ND	ND	ND	N	TP	NP
21	ND	NP	NP	ND	N	NP	NP
22	ND	NP	NP	ND	P	NP	NP
23	ND	ND	NP	ND	P	NP	NP
24	ND	ND	NP	ND	P	TP	I
25	D	NP	NP	ND	P	TP	I
26	ND	ND	NP	ND	N	NP	NP
27	ND	NP	NP	ND	N	NP	NP
28	ND	NP	D	D	P	DPC	I
29	ND	ND	NP	ND	P	DPC	NB
30	ND	ND	NP	ND	P	NP	NP

CT: computed tomography; MRI: magnetic resonance imaging; intraoperative US: intraoperative ultrasonography; ASVS: intra-arterial calcium stimulation test with hepatic venous sampling; ND: nondiagnostic; NP: nonperformed; D: diagnostic; P: positive; N: negative; DPC: duodenopancreatectomy; TP: total pancreatectomy; TE: tumor enucleation; I: insulinoma; NB: nesidioblastosis.

Table 2: Indices for evaluating diagnostic tests for the detection of insulinoma.

	S	E	PPV	NPV	PR	NR
CT	35.71%	100%	100%	25%		0.64
MRI	16.67%	100%	100%	37.5%	0.83	0.58
Intraoperative US	53.33%	100%	100%	36.36%		0.47
Arteriography	43.75%	100%	100%	35.71%		0.56
ASVS	100%	20%	80%	100%	1.25	

CT: computed tomography; MRI: magnetic resonance imaging; intraoperative US: intraoperative ultrasonography; ASVS: intra-arterial calcium stimulation test with hepatic venous sampling; S: sensitivity; E: specificity; PPV: positive predictive value; NPV: negative predictive value; NR: negative likelihood ratio; PR: positive likelihood ratio.

84–100% tumors located in the head or body of the pancreas, but this technique has a decreased sensitivity in lesions located in the tail of pancreas (60%) [7]. Selective arteriography has been traditionally proposed by many authors as the choice test for location due to its sensitivity, which reaches 40–90% [22]; this is an invasive procedure, where frequent anatomical variability must be evaluated; it also requires high contrast doses and radiation exposure [24].

It has been reported that intraoperative US and pancreatic palpation by experienced surgeons could locate up to 100% of insulinomas [23]. The intraoperative US has a sensitivity of 95%,but in our series does not exceed 55%, probably due to the tumor size and the surgeon expertise.

One limitation of this study is related to the low incidence of insulinomas being rare tumors; the comparison of different localization techniques is difficult because series are usually collected over a period of years; changes and advances in imaging techniques may substantially alter the detection rate.

In our study, the ASVS, evaluated as a functional and morphological technique, was the procedure that allowed the confirmation of pathological insulin production due to a pancreatic mass or islet hyperplasia as in nesidioblastosis. For all of this, according to our results and those published by other authors [1, 2, 5, 11, 13, 16, 20], we suggest the ASVS as the preoperative invasive test of choice when an insulinoma is suspected.

Competing Interests

All the authors declare that there is no conflict of interests that could be perceived as prejudicing the impartiality of the research reported.

References

[1] P. E. Cryer, L. Axelrod, A. B. Grossman et al., "Evaluation and management of adult hypoglycemic disorders: an Endocrine Society Clinical Practice Guideline," *The Journal of Clinical Endocrinology & Metabolism*, vol. 94, no. 3, pp. 709–728, 2009.

[2] P. M. Moreno, C. G. Alcántara, M. del Carmen Muñoz-Villanueva et al., "Usefulness of arterial calcium stimulation with hepatic venous sampling in the localization diagnosis of endogenous hyperinsulinism," *Endocrinología y Nutrición*, vol. 57, no. 3, pp. 95–99, 2010.

[3] T. Okabayashi, Y. Shima, T. Sumiyoshi et al., "Diagnosis and management of insulinoma," *World Journal of Gastroenterology*, vol. 19, no. 6, pp. 829–837, 2013.

[4] M. R. Druce, V. M. Muthuppalaniappan, B. O'Leary et al., "Diagnosis and localisation of insulinoma: the value of modern magnetic resonance imaging in conjunction with calcium stimulation catheterisation," *European Journal of Endocrinology*, vol. 162, no. 5, pp. 971–978, 2010.

[5] R. Palomares, L. Zurera, M. A. Gálvez, S. Tofé, M. Canis, and P. Benito, "Utilidad de la arteriografía con inyección intraarterial selectiva de calcio en el diagnóstico del insulinoma," *Medicina Clínica*, vol. 119, no. 15, pp. 568–570, 2002.

[6] F. J. Service, M. M. McMahon, P. C. O'Brien, and D. J. Ballard, "Functioning insulinoma—incidence, recurrence, and long-term survival of patients: a 60-year study," *Mayo Clinic Proceedings*, vol. 66, no. 7, pp. 711–719, 1991.

[7] K. A. Placzkowski, A. Vella, G. B. Thompson et al., "Secular trends in the presentation and management of functioning insulinoma at the mayo clinic, 1987–2007," *Journal of Clinical Endocrinology and Metabolism*, vol. 94, no. 4, pp. 1069–1073, 2009.

[8] T. C. Noone, J. Hosey, F. Zeynep, and R. C. Semelka, "Imaging and localization of islet-cell tumours of the pancreas on CT and MRI," *Best Practice and Research: Clinical Endocrinology and Metabolism*, vol. 19, no. 2, pp. 195–211, 2005.

[9] O. N. Tucker, P. L. Crotty, and K. C. Conlon, "The management of insulinoma," *British Journal of Surgery*, vol. 93, no. 3, pp. 264–275, 2006.

[10] G. McAuley, H. Delaney, J. Colville et al., "Multimodality preoperative imaging of pancreatic insulinomas," *Clinical Radiology*, vol. 60, no. 10, pp. 1039–1050, 2005.

[11] J.-M. Guettier, A. Kam, R. Chang et al., "Localization of insulinomas to regions of the pancreas by intraarterial calcium stimulation: the NIH experience," *Journal of Clinical Endocrinology and Metabolism*, vol. 94, no. 4, pp. 1074–1080, 2009.

[12] B. K. P. Goh, L. L. P. J. Ooi, P.-C. Cheow et al., "Accurate preoperative localization of insulinomas avoids the need for blind resection and reoperation: analysis of a single institution experience with 17 surgically treated tumors over 19 years," *Journal of Gastrointestinal Surgery*, vol. 13, no. 6, pp. 1071–1077, 2009.

[13] A. Bassam and B. Joe, "Occult sporadic insulinoma: localization and surgical strategy," *World Journal of Gastroenterology*, vol. 14, no. 5, pp. 657–665, 2008.

[14] R. Sotoudehmanesh, A. Hedayat, N. Shirazian et al., "Endoscopic ultrasonography (EUS) in the localization of insulinoma," *Endocrine*, vol. 31, no. 3, pp. 238–241, 2007.

[15] L.-M. Tseng, J.-Y. Chen, J. G.-S. Won et al., "The role of intra-arterial calcium stimulation test with hepatic venous sampling (IACS) in the management of occult insulinomas," *Annals of Surgical Oncology*, vol. 14, no. 7, pp. 2121–2127, 2007.

[16] J. L. Doppman, D. L. Miller, R. Chang, T. H. Shawker, P. Gorden, and J. A. Norton, "Insulinomas: localization with selective intra-arterial injection of calcium," *Radiology*, vol. 178, no. 1, pp. 237–241, 1991.

[17] J. L. Doppman, "Questions and answers," *American Journal of Roentgenology*, vol. 168, no. 5, pp. 1376–1377, 1997.

[18] S. Morita, H. Machida, R. Kuwatsuru et al., "Preoperative localization of pancreatic insulinoma by super selective arterial stimulation with venous sampling," *Abdominal Imaging*, vol. 32, no. 1, pp. 126–128, 2007.

[19] M. Imamura, K. Takahashi, H. Adachi et al., "Usefulness of selective arterial secretin injection test for localization of gastrinoma in the Zollinger-Ellison syndrome," *Annals of Surgery*, vol. 205, no. 3, pp. 230–239, 1987.

[20] P. L. Pereira, A. J. Roche, G. W. Maier et al., "Insulinoma and islet cell hyperplasia: value of the calcium intraarterial stimulation test when findings of other preoperative studies are negative," *Radiology*, vol. 206, no. 3, pp. 703–709, 1998.

[21] M. L. del Olmo Martínez, P. González de Canales Simón, and M. T. Arranz Santos, "Tumores endocrinos del páncreas," *Medicine*, vol. 8, no. 8, pp. 404–414, 2000.

[22] N. M. Kuzin, A. V. Egorov, S. A. Kondrashin, A. N. Lotov, N. S. Kuznetzov, and J. B. Majorova, "Preoperative and intraoperative topographic diagnosis of insulinomas," *World Journal of Surgery*, vol. 22, no. 6, pp. 593–598, 1998.

[23] M. Dieguez, M. Riestra, and E. Menéndez, "Insulinoma: criterios diagnósticos y tratamiento," *Avances en Diabetología*, vol. 25, no. 4, pp. 293–299, 2009.

[24] M. Brändle, T. Pfammatter, G. A. Spinas, R. Lehmann, and C. Schmid, "Assessment of selective arterial calcium stimulation and hepatic venous sampling to localize insulin-secreting tumours," *Clinical Endocrinology*, vol. 55, no. 3, pp. 357–362, 2001.
