# Supplementary material for: Diagnostic value of ASVS for insulinoma localization: A systematic review and meta-analysis
Source: PLoS One. 2019 Nov 19;14(11):e0224928. doi: 10.1371/journal.pone.0224928 (PMC6863549; doi:10.1371/journal.pone.0224928)
Supplement: S2 File — (ZIP) [file pone.0224928.s002.zip › included studies/comparison of two dose of ASVS.pdf]

 doi: 10.1111/cen.12253

ORIGINAL ARTICLE

Comparison of two low-dose calcium infusion schedules for localization of insulinomas by selective pancreatic arterial injection with hepatic venous sampling for insulin

G. Braatvedt, E. Jennison and I. M. Holdaway

Department of Endocrinology, Greenlane Clinical Centre and Auckland City Hospital, Auckland, New Zealand

Summary

Objective Localization of small insulinomas may be difficult. Selective pancreatic arterial injection of calcium with hepatic venous insulin measurement (SACST) has been used for this purpose, but can rarely cause hypoglycaemia. Two low-dose concentrations of calcium, 0·25 and 0·1 of the usual concentration used for the test, have been compared for sensitivity of localization and safety.

Design Selective pancreatic arterial injection of calcium with hepatic venous insulin measurement was performed at calcium concentrations of 0·0025 (Protocol A) and 0·00625 (Protocol B) mEq calcium per kg. The standard concentration is 0·025 mEq/kg.

Patients Twenty one successive patients with biochemical evidence of insulinoma were studied.

Results Using surgical localization as the gold standard, Protocol A had a sensitivity of 91% and Protocol B 75% for correct localization. The false-positive localization rate was 16%. No hypoglycaemia was observed. These results compare favourably with published data using the standard calcium concentration. Selective pancreatic arterial injection of calcium with hepatic venous insulin measurement was superior to localization by noninvasive imaging; in seven cases, SACST was correct when conventional imaging was negative (five) or false positive (two).

Conclusion Low concentrations of calcium are effective and safe when performing SACST for localization of insulinoma.

(Received 6 February 2013; returned for revision 20 May 2013; finally revised 21 May 2013; accepted 22 May 2013)

Introduction

Although the biochemical diagnosis of insulinomas is straightforward, the clinical challenge lies in localization of the tumour within the pancreas. Preoperative localization is essential to increase the chances of surgical success, minimize operating room time, reduce the rate of recurrence and avoid unnecessary resection of the pancreas.[1,2] A number of noninvasive localization techniques are available including ultrasonography (US), computed tomography (CT), magnetic resonance imaging (MRI) and photon emission tomography (PET), as well as older invasive procedures such as pancreatic angiography and transhepatic portal venous sampling. In general, the sensitivity of these imaging techniques has been reported to be low. Furthermore, despite advances in the technology of scanners, a recent study by the National Institute of Health found that there has been no improvement in the sensitivity of present-day noninvasive imaging tests for localization of insulinomas compared with those available two decades ago.[3] More recent invasive localization modalities include endoscopic US (EUS), intraoperative US and selective intra-arterial calcium stimulation of insulin release by injection into the major pancreatic arteries with hepatic venous sampling for serum insulin (SACST). Although these have all had generally superior detection rates compared with non-invasive scans, SACST has been particularly successful, with reported successful localization rates of 67–100%.[4–8]

Doppman and co-workers originally described the SACST technique in 1991 using calcium doses of 0·01–0·025 mEq/kg.[9] This has been successfully repeated by numerous other studies using the 0·025 mEq/kg dosage.[3–8] In an effort to lower the dose of calcium required and reduce the risk of hypoglycaemia, O'Shea *et al.*[10] titrated the dose of calcium gluconate to 0·00625 mEq/kg, which they found to be equally as effective as the 0·025 mEq/kg dose used by Doppman *et al.*[9] In the present study, the sensitivity of the SACST technique has been studied using two low doses of calcium gluconate, 0·00625 mEq/kg and 0·0025 mEq/kg.

Methods

Patients

Twenty one successive patients, 19 female and two male (mean age 55 year, range 32–73), were diagnosed with a probable

Correspondence: Ian M. Holdaway, Dept of Endocrinology, Greenlane Clinical Centre, PO Box 92189, Victoria St West, Auckland 1142, New Zealand. Tel.: 0064 9 367 0000; Fax: 0064 93074993;
E-mail: ian@adhb.govt.nz

 © 2013 John Wiley & Sons Ltd

insulinoma at Auckland City Hospital between 1995 and 2011. Diagnosis of insulinoma was based on the development of symptomatic hypoglycaemia with inappropriately elevated levels of plasma insulin and C-peptide during fasting and resolution of symptoms with glucose administration. Other causes of hypoglycaemia were excluded by usual means. Baseline data for the whole patient group are shown in Table 1.

SACST procedure

A SACST study was performed on each patient prior to surgery according to the protocol of Doppman *et al.*[9] using two different calcium concentrations for arterial injection. Between 1994 and 2004, a calcium dose of 0·0025 mEq/kg was used (Protocol A), and between 2005 and 2011, a dose of 0·0065 mEq/kg (Protocol B) was employed. The calcium concentration used by Doppman *et al.* was 0·025 mEq/kg, but was erroneously stated as 0·0025 mEq/kg in the original paper.[9] The initial ultralow dose used for Protocol A in the present study was adopted from the original Doppman paper before the subsequent correction[11] was recognized, and was then continued in view of apparently satisfactory results, but was changed in 2005 to Protocol B employing the calcium concentration used by O'Shea *et al.*[10] which is one quarter of the dose used by Doppman *et al.*[9,11] Hepatic venous glucose was monitored frequently during the procedure using a HemoCue machine, and intravenous 5% glucose was infused and adjusted as needed to maintain euglycaemia. Attempts were made to cannulate the standard pancreatic arterial regions described by Doppman *et al.*[9] (gastroduodenal, SMA, splenic and proper hepatic), but on occasions the radiologists cannulated other pancreatic arteries and also injected calcium at proximal, mid and distal splenic artery sites.

Table 1. Baseline patient data

Patient no	Sex	Age	Glucose nadir (mmol/l) (time h*)	Insulin at glucose nadir (mIU/l)
1	F	50	1·9 (8)	18
2	F	51	1·4 (45)	19·3
3	F	73	1·5 (12)	109
4	F	77	1·4 (12)	47
5	F	61	2·3 (13)	20
6	F	42	2 (12)	12·1
7	F	58	2 (20)	10
8	F	52	1·9 (12)	14
9	F	32	1·5 (15)	10
10	M	31	1·5 (8)	100
11	F	68	1·4 (6)	23
12	F	53	2·1 (20)	3·1
13	F	42	1·5 (13)	12·9
14	M	47	1·9 (12)	148
15	F	63	1·9 (14)	18·9
16	F	37	2·1 (17)	4
17	F	48	2·1 (43)	5·3
18	F	69	2 (25)	41·6
19	F	64	2·5 (14)	7
20	F	61	1·8 (13)	10·1
21	F	47	1·8 (6)	26·8

*hours fasting.

Insulin assay

Insulin levels were determined in samples 1994–2004 by Abbot IMX immunoassay and subsequently by Abbott Architect sandwich-type chemiluminescent immunoassay. The coefficient of variation was ±2·8% at an insulin level of 38 mIU/l with either assay. There was no cross-reaction with proinsulin in the assays, and basal proinsulin measurements were unfortunately not available.

Surgery

Surgical resection of the insulinoma was performed in 19 of the patients. Two patients from the Protocol B group did not have surgery: patient 18 had major comorbidities and has subsequently been managed conservatively with frequent meals and has remained symptom-free, and patient 20 did not have a localized lesion and has been managed successfully with diazoxide therapy. The decision on site of resection was at the discretion of the surgeon, who in some instances performed resections at regions other than predicted by SACST.

Results

The procedure was well tolerated with no significant complications. Most individuals experienced minor flushing with each calcium infusion, but this was not troublesome. The intensity of flushing was not graded in a systematic fashion, but the nurses involved in the procedure noted that flushing and tingling was milder in the very low-dose calcium group (protocol A) than in protocol B. No patient experienced hypoglycaemia.

The definition of a positive response to calcium infusion in a given artery has not been rigorously defined in the literature on the SACST procedure. Traditionally, a twofold rise in serum insulin concentration has been used to define a sufficient increase to indicate localization of an insulinoma perfused by the injected artery. In order to estimate the increment in insulin production that would indicate a positive response using the current calcium dosages, the individual SACST test results were reviewed to compare the fold step-up in hepatic vein insulin concentration following injection into arteries at the opposite end of the pancreas from where the tumour was detected surgically in those with histologically confirmed tumours (e.g. the splenic artery for patients who had insulinomas in the head of the pancreas, or the gastroduodenal artery or hepatic artery for those with tumours in the pancreatic tail). The insulin increments following calcium injection into arteries in these regions are shown in Table 2 and were used to provide control data for noninsulinoma islet tissue. For Protocol A, the mean insulin increment compared with baseline in noninvolved regions was 1·0 (range 0·7–1·2) for patients with proven insulinomas (patients 1–10), and for Protocol B was 1·1 (range

© 2013 John Wiley & Sons Ltd

Table 2. Results of localization tests and surgery

Patient no	Pre-SACST scans and localization*	Artery with maximum insulin increase†	Artery with minimal insulin increase‡	SACST localization	Site of tumour at surgery	Histology
Group A: Calcium infusion 0·0025 mEq/kg						
1	C = neg	Hepatic (6·2)	Distal splenic (1)	Head	Head	Insulinoma
2	C = neg. U = neck	SMA (2·2)	Distal splenic (0·9)	Inferior head	Inferior head	Insulinoma
3	Nil	Gastroduodenal (2·5)	Distal splenic (1)	Head	Anterior head	Insulinoma
4	Nil	Distal splenic (8·4)	Hepatic (0·8)	Body/tail	Tail	Insulinoma
5	C = body, U = body	Coeliac (5·5)	Distal splenic (0·9)	Head	Superior neck	Insulinoma
6	C = neg. U = neg	Dorsal pancreatic and splenic (1·3)	Hepatic (1)	Body/tail	Inferior body	Insulinoma
7	M = neg	Distal splenic (17·5)	Gastroduodenal (1)	Tail	Tail	Insulinoma
8	C = neg	Hepatic (1·9)§	Nil available	Head	Head	Insulinoma
9	M = tail	Superior pancreaticoduodenal (1·8)	Hepatic (0·7)	Mid body	Between body/tail	Insulinoma
10	C = neg, U = neg	Distal splenic (3·8)	Gastroduodenal (1·2)	Tail	Between body/tail	Insulinoma
11	C = neg	Common hepatic (2·7)	Distal splenic (1·2)	Head	Nil	Normal pancreas
Group B: Calcium infusion 0·0065 mEq/kg						
12	C = neg, M = tail	Prox splenic (62)	Gastroduodenal (1·1)	Body	Body	Insulinoma
13	C = neg, EU = neg P = head	Gastroduodenal (31)	Distal splenic (0·5)	Head	Head	Insulinoma
14	C = inferior tail M = body/tail	Prox splenic (12·7)	Gastroduodenal (1·1)	Body	Body	Insulinoma
15	C = tail	Mid splenic (4·4)	Gastroduodenal (1·5)	Tail	Tail	Insulinoma
16	C = neg, M = tail	Distal splenic (6·1)	Gastroduodenal (1·1)	Tail	Tail	Insulinoma
17	C = body, M = neg EU = neg	SMA (4)	Distal splenic (1·1)	Inferior head	Neck	Insulinoma
18	C = neg, EU = neg	Coeliac (2·4)	Gastroduodenal (1·7)	Uncertain	No surgery	No surgery
19	C = neg, M = ?tail 0 = neg	SMA (3·2)¶	Distal splenic (2·4)	Head	Nil detected	Nesidioblastosis
20	C = neg, M = neg, EU = neg	Not identified	Not identified	Not identified	No surgery	No surgery
21	C = tail	Gastroduodenal (8·8)	Splenic (1·2)	Head	Not identified**	Normal pancreas**

*C, CT scan; M, MRI scan; U, transabdominal ultrasound scan; EU, endoscopic ultrasound scan; O, octreoscan; P, PET scan.

†() = ratio of maximum to basal insulin value following calcium infusion

‡Artery at opposite end of pancreas to the site of maximum insulin increase. () = ratio of maximum to basal insulin value following calcium infusion

§Only three vessels able to be cannulated (hepatic, coeliac and SMA)

¶Significant increase by over twofold in several arterial territories so localization uncertain presurgery.

**CT scan identified mass adjacent to pancreatic tail. No insulinoma found at surgery. Distal pancreatectomy performed with no lesion found on histology. Postoperative CT shows persisting mass adjacent to tail of pancreas, ? accessory spleen.

© 2013 John Wiley & Sons Ltd

0·5–1·5) for patients 12–17 with proven insulinomas. A positive response to calcium infusion was thus defined as a step-up in insulin concentration of >1·3 for Protocol A patients and >1·5 for the Protocol B group.

The results of the SACST test are shown in Table 2, together with the findings from preoperative pancreatic imaging. In Protocol A patients, a positive increment in serum insulin (>1·3) was seen in all 11 patients, and an insulinoma was found at the expected site in 10 individuals. Patient 11 had a positive insulin response (×2·7) in the common hepatic artery suggesting a lesion in the neck or head of the pancreas, and a lesser increment of 1·9 in the coeliac axis artery. A preoperative CT scan was nondiagnostic. No tumour was detected at surgery. A distal pancreatectomy was performed but the patient was not cured and histology showed normal islet tissue. The patient has subsequently been managed without specific therapy and has had only occasional further documented hypoglycaemia. Six patients in the Protocol B group had an insulinoma at the site predicted from the SACST study. Patient 19 demonstrated a significant insulin increment from infusion into the superior meseteric artery and was thought to most likely have a lesion near the head of the pancreas. No tumour was found at surgery, but a distal pancreatectomy showed nesidioblastosis. In retrospect, the distal splenic artery territory also showed a significant increment of 2·4, and the positive response in two widely separate arterial regions was consistent with diffuse islet cell disease. She has subsequently been managed with medical therapy. Patient 18 had a SACST result which was uncertain (positive in the coeliac axis, supplying multiple pancreatic regions), and elected not to proceed to surgery and has been treated medically. Patient 20 did not show a significant increment with calcium infusion in any arterial territory with SACST and did not localize using other imaging and has also been treated medically. The SACST in patient 21 predicted a tumour in the pancreatic body, whereas CT scan indicated a lesion 'adjacent to' the pancreatic tail which was not detected at surgery and which persists on CT scan and may be unrelated (? accessory spleen). This patient has unfortunately defaulted from further follow-up.

Analysis of the SACST results with Protocol A indicates a sensitivity of 91% for tumour localization and may indeed be 100% since the predicted lesion in the pancreatic neck in patient 11 has not been excluded histologically. Patient 11 has however been considered a false-positive result. With Protocol B, the correct prediction from SACST was six of eight patients coming to surgery (75%). The 25% false-positive localization may be less than stated, since the tumour localization in patient 21 remains uncertain. As mentioned above, patient 19 was probably misclassified in the SACST analysis. Patients 18 and 20 did not undergo surgery so the localizing ability of the SACST procedure is uncertain; a tumour was not localized in either patient. If these are considered, in the worst case scenario, as incorrect localization, then the positive prediction rate falls to 6/10 (60%) with Protocol B.

Discussion:

This study compares the use of two different concentrations of calcium in the performance of the SACST procedure for localization of insulinoma in patients with biochemically proven hypoglycaemia and endogenous insulin excess. The results indicate that calcium concentrations lower than in the original published procedure[9] are effective in provoking insulin release from insulinomas and can localize tumours within the pancreas. Protocol A, using one tenth of the calcium concentration described by Doppman *et al.*[9] correctly identified the site of an insulinoma in 10 of 11 patients, using subsequent surgical localization as the gold standard. There was one false-positive result (patient 11) in whom an arterial territory showing maximum insulin release was thought to indicate a tumour in the head/neck region of the pancreas, but no tumour was identified at surgery. Normal pancreatic tissue was found histologically in the tail of the pancreas. It remains possible that this patient has a small tumour in the head of the pancreas which was not apparent at surgery, but the patient has been classified as a false positive.

Protocol B, using one quarter of the original calcium concentration suggested by Doppman *et al.*[9] and utilized by O'Shea *et al.*[10] correctly identified the site of an insulinoma in six of eight patients coming to surgery. Patient 19 was considered a false-positive result, with the SACST indicating a tumour in the head of the pancreas, but in retrospect, there was a significant although lesser insulin release with calcium injection in another arterial territory at the opposite end of the pancreas, which should have alerted us to the possibility of nesidioblastosis, which was the eventual diagnosis. The final diagnosis in patient 21 remains uncertain, with a mass adjacent to the pancreatic tail seen on CT scan but not identified or removed at surgery, whereas the SACST identified a likely tumour in the body of the pancreas. This patient has defaulted from follow-up so the final diagnosis remains uncertain, but for the purposes of this study, the patient has been classified as a false-positive result.

Assessing patients 11, 19 and 21 as false-positive localization by SACST indicates a correct overall positive detection rate of 16/19 patients (84%). This compares with correct localization rates in other reports of 63% (Druce *et al.*),[12] 93% (Placzkowski *et al.*[13]) and 84% (Guettier *et al*).[3] A false-positive localization rate of 3/19 (16%) compares with rates of 4% in the NIH series,[3] and 15% in the report from St Bartholomew's Hospital.[12]

The rates of correct localization in the present study appear to be superior to those obtained with other modalities of localization using noninvasive scanning. In those with tumours localized by surgery, CT scan (in 15 patients) correctly identified the tumour site in just two subjects, with one false positive and 12 false negatives. The corresponding figures for transabdominal ultrasound were one true positive, two false negatives and two false positives, and for MRI scan three true positives, two false positives and one false negative. Photon emission tomography and octreoscan were performed in one subject each with one false positive (octreoscan) and one true positive (PET). Localization by conventional imaging agreed with SACST findings in seven patients. However, SACST provided correct localization in seven subjects in whom conventional imaging was either negative (n = 5) or not done (n = 2), and in two cases where conventional imaging gave incorrect results. In the other major series,[3,12,13] CT scan had a sensitivity of 32–57%, MRI scan 25–66% and transabdominal ultrasound 14–61% for correct

© 2013 John Wiley & Sons Ltd

tumour localization. Endoscopic ultrasound was only obtained in two patients in the current series, both with false-negative results. However, in experienced hands this technique has been reported to provide localization in up to 90% of patients with tumours in the head of the pancreas.[14] The results of intraoperative ultrasound were only recorded in a minority of patients in the current series but can be helpful in those undergoing surgery.[15]

No hypoglycaemic episodes were recorded with either calcium dose. This side effect is extremely rare in reports of the use of SACST, mainly because intravenous glucose and frequent glucose monitoring is used to maintain euglycaemia. However, severe hypoglycaemia was noted in one patient by O'Shea *et al.*[10] using the standard calcium dose described by Doppman,[9] which led to them adopting a lower calcium concentration of 0·0065 mEq/kg as used in Protocol B in the present study. The severity of flushing and tingling in the current report appeared less using the lowest calcium dose (Protocol A).

Use of lower doses of calcium than in the standard procedure required evaluation of what constitutes a normal response from uninvolved pancreatic tissue compared with a positive (exaggerated) response from a region of the pancreas containing an insulinoma. The Doppman protocol,[9] adopted in other reports,[3,12,13] defines a twofold increase in insulin as indicating a positive response. The reliability of this criterion does not seem to have been rigorously evaluated but may have been drawn from the results of studies using transhepatic portal venous sampling. In the present study, the mean increment in insulin release from the insulinoma tissue using protocol A was a fourfold increase (range 1·3–17·5) and for the higher dosage (protocol B) was an 11-fold increment (range 2·4-62). Using a twofold increase in hepatic venous insulin concentration as indicating a positive response would not change the results in group B but would have altered the results in group A by reclassifying three individuals (patients 7, 9 and 10) as having negative tests. By assessing increments from injection into arteries supplying noninvolved regions of the pancreas remote from the site of maximum insulin release, we were able to define an appropriate cut-off for significant insulin release. In protocol A, the appropriate detection of insulinomas at surgery using this criterion (a 1·3-fold increase or greater being abnormal) in patients six, eight and nine supports this definition. However, if a twofold increase in insulin concentration is adopted as defining a positive response for all patients, the sensitivity of the procedure in group A would fall to 7 of 11 (64%) with three false negatives and one false-positive result.

The present study was performed in a limited number of patients, so the results should be interpreted with caution. Nonetheless, the study indicates that successful localization of insulinomas can be achieved by SACST using lower concentrations of calcium than previously described, and this reduction in calcium dose per artery may be associated with a lower rate of side effects. Although both calcium doses provided satisfactory localization rates, protocol B is probably preferable for routine use given published experience with this calcium dosage.[10] However, correct localization of insulinomas remains a challenge, with a low rate of correct localization using conventional noninvasive scans and a small but significant rate of false-positive localization with SACST in this and other reports. Overall, SACST provides the best current means for localization of small insulinomas.

Conflict of interest

Nothing to declare.

References

1 Abboud, B. & Boujaoude, J. (2008) Occult sporadic insulinoma: Localization and surgical strategy. *World Journal of Gastroenterology*, **14**, 657–665.

2 Pasieka, J.L., McLeod, M.K., Thompson, N.W. *et al.* (1992) Surgical approach to insulinomas. Assessing the need for preoperative localization. *Archives of Surgery*, **127**, 442–447.

3 Guettier, J.M., Kam, A., Chang, R. *et al.* (2009) Localization of insulinomas to regions of the pancreas by intra-arterial calcium stimulation: the NIH experience. *Journal of Clinical Endocrinology and Metabolism*, **94**, 1074–1080.

4 Doppman, J.L., Chang, R., Fraker, D.L. *et al.* (1995) Localization of insulinomas to regions of the pancreas by intra-arterial stimulation with calcium. *Annals of Internal Medicine*, **123**, 269–273.

5 Lo, C.Y., Lam, K.Y., Kung, A.W. *et al.* (1997) Pancreatic insulinomas: a 15-year experience. *Archives of Surgery*, **132**, 926–930.

6 Ravi, K. & Britton, B.J. (2007) Surgical approach to insulinomas: are preoperative localization tests necessary. *Annals of the Royal College of Surgeons of England*, **89**, 212–217.

7 Hiramoto, J.S., Feldstein, V.A., LaBerge, J.M. *et al.* (2001) Intraoperative ultrasound and preoperative localization detects all occult insulinomas. *Archives of Surgery*, **136**, 1020–1026.

8 Lo, C.Y., Chan, F.L., Tam, S.C. *et al.* (2000) Value of intra-arterial calcium stimulated venous sampling for regionalization of pancreatic insulinomas. *Surgery*, **128**, 903–909.

9 Doppman, J.L., Miller, D.L., Chang, R. *et al.* (1991) Insulinomas: localization with selective intraarterial injection of calcium. *Radiology*, **178**, 237–241.

10 O'Shea, D., Rohrer-Theurs, A.W., Lynn, J.A. *et al.* (1996) Localization of insulinomas by selective intraarterial calcium injection. *Journal of Clinical Endocrinology and Metabolism*, **81**, 1623–1627.

11 (1993) Erratum. *Radiology*, **187**, 880.

12 Druce, M.R., Muthuppalaniappan, V.M., O'Leary, B. *et al.* (2010) Diagnosis and localisation of insulinoma: the value of modern magnetic resonance imaging in conjunction with calcium stimulation catheterisation. *European Journal of Endocrinology*, **162**, 971–978.

13 Placzkowski, K.A., Vella, A., Thompson, G.B. *et al.* (2009) Secular trends in the presentation and management of functioning insulinoma at the Mayo Clinic, 1987-2007. *Journal of Clinical Endocrinology and Metabolism*, **94**, 1069–1073.

14 Fritscher-Ravens, A. (2004) Endoscopic ultrasound and neuroendocrine tumours of the pancreas. *Journal of the Pancreas*, **5**, 273–281.

15 Zhao, Y.P., Zhan, H.X., Zhang, T.P. *et al.* (2011) surgical management of patients with insulinomas: Result of 292 cases in a single institution. *Journal of Surgical Oncology*, **103**, 169–174.

© 2013 John Wiley & Sons Ltd
