# Supplementary material for: Diagnostic value of ASVS for insulinoma localization: A systematic review and meta-analysis
Source: PLoS One. 2019 Nov 19;14(11):e0224928. doi: 10.1371/journal.pone.0224928 (PMC6863549; doi:10.1371/journal.pone.0224928)
Supplement: S2 File — (ZIP) [file pone.0224928.s002.zip › included studies/Diagnosis and localisation of insulinoma_ the value of modern magnetic resonance imaging in conjunction with calcium stimulation catheterisation.pdf]

CLINICAL STUDY

Diagnosis and localisation of insulinoma: the value of modern magnetic resonance imaging in conjunction with calcium stimulation catheterisation

Maralyn R Druce[1], Vasantha M Muthuppalaniappan[1], Benjamin O'Leary[1], Shern L Chew[1], William M Drake[1], John P Monson[1], Scott A Akker[1], Michael Besser[1], Anju Sahdev[2], Andrea Rockall[2], Soumil Vyas[3], Satya Bhattacharya[3], Matthew Matson[2], Daniel Berney[4] and Ashley B Grossman[1]

Departments of [1]Endocrinology, [2]Radiology, [3]Surgery and [4]Histopathology, Barts and the London Medical School, St Bartholomew's Hospital, London EC1A 7BE, UK

(Correspondence should be addressed to M R Druce; Email: maralyn.druce@bartsandthelondon.nhs.uk)

Abstract

Context: Preoperative localisation of insulinoma improves cure rate and reduces complications, but may be challenging.
Objective: To review diagnostic features and localisation accuracy for insulinomas.
Design: Cross-sectional, retrospective analysis.
Setting: A single tertiary referral centre.
Patients: Patients with insulinoma in the years 1990–2009, including sporadic tumours and those in patients with multiple endocrine neoplasia syndromes.
Interventions: Patients were identified from a database, and case notes and investigation results were reviewed. Tumour localisation by computed tomography (CT), magnetic resonance imaging (MRI), octreotide scanning, endoscopic ultrasound (EUS) and calcium stimulation was evaluated.
Main outcome measure(s): Insulinoma localisation was compared to histologically confirmed location following surgical excision.
Results: Thirty-seven instances of biochemically and/or histologically proven insulinoma were identified in 36 patients, of which seven were managed medically. Of the 30 treated surgically, 25 had CT (83.3%) and 28 had MRI (90.3%), with successful localisation in 16 (64%) by CT and 21 (75%) by MRI respectively. Considered together, such imaging correctly localised 80% of lesions. Radiolabelled octreotide scanning was positive in 10 out of 20 cases (50%); EUS correctly identified 17 lesions in 26 patients (65.4%). Twenty-seven patients had calcium stimulation testing, of which 6 (22%) did not localise, 17 (63%) were correctly localised, and 4 (15%) gave discordant or confusing results.
Conclusions: Preoperative localisation of insulinomas remains challenging. A pragmatic combination of CT and especially MRI predicts tumour localisation with high accuracy. Radionuclide imaging and EUS were less helpful but may be valuable in selected cases. Calcium stimulation currently remains useful in providing an additional functional perspective.

European Journal of Endocrinology **162** 971–978

Introduction

Insulinoma is reported to be the most common cause of hypoglycaemia in patients who are well without systemic illness, once factitious hypoglycaemia has been excluded (1). However, it is a rare tumour, with an estimated incidence of 4 per million population per year (2).

The clinical diagnosis of hypoglycaemia is generally based on Whipple's triad: the occurrence of symptoms consistent with hypoglycaemia, a low plasma glucose concentration at the time of symptoms and the relief of symptoms associated with the correction of the hypoglycaemia. The diagnosis of hyperinsulinaemic hypoglycaemia is established by demonstrating inappropriately high serum insulin concentrations during fasting hypoglycaemia, specifically a prolonged supervised fast (3). The original suggested diagnostic criterion for an insulinoma was an insulin level >6 mU/l in the presence of hypoglycaemia, measured by a double-antibody RIA with a lower limit of detection of 5 mU/l (4, 5). With the introduction of new highly specific insulin assays, lower levels of insulin have been detected in patients with insulinomas, and thus, new lower diagnostic criteria have been proposed by some groups (1, 6–8). A recent consensus set of guidelines for the diagnosis of inappropriate hyperinsulinemia in

© 2010 European Society of Endocrinology

DOI: 10.1530/EJE-10-0056
Online version via www.eje-online.org

Downloaded from Bioscientifica.com at 04/02/2019 12:59:21PM
via free access

the presence of documented hypoglycaemia have proposed a diagnostic threshold of plasma insulin of 3 mU/l (9). The diagnosis of insulinoma rests on the combination of hyperinsulinaemic hypoglycaemia and the exclusion of alternative diagnoses (for example, by measurement of C-peptide to exclude exogenous insulin administration, and measurement of a plasma or urine sulphonylurea screen to exclude the covert use of such medications, although not all oral hypoglycaemic agents, for example repaglinide, can be detected in this way) (9).

At the time of diagnosis, the vast majority of insulinomas are small, intra-pancreatic and curable by surgery. It might be supposed that preoperative localisation improves the chance of cure and reduces the likelihood of complications, although there is a lack of study data to support this contention. Such localisation can prove to be a clinical challenge. Cross-sectional imaging techniques such as computed tomography (CT) scanning and magnetic resonance imaging (MRI) have been extensively used, but published data have not in general shown these to be particularly accurate, with reported sensitivities in the range 17–50% (10–12), although occasional series suggest sensitivities much higher. For example, a CT sensitivity of 94% using multidetector scanning and fine reformats has been demonstrated (13), while MRI sensitivities have been described of 79% for delayed enhanced T1-weighted images (14) or with the use of combined sequences up to 85% (15). Endoscopic ultrasound (EUS) is evolving and improving, and may provide an opportunity for histological diagnosis, but this semi-invasive technique is highly dependent on operator experience (16, 17). Selective intra-arterial injection of the pancreatic arteries with calcium and hepatic venous sampling for insulin, first introduced 17 years ago (18), correlates anatomy with function, and in many series, it appears to be the most sensitive method for regionalisation of insulinoma. In a previous series of 25 surgically proven sporadic insulinomas, calcium stimulation correctly regionalised 88% of tumours, compared to MRI in 43% and CT in only 17% (19), and this high diagnostic accuracy was maintained in a recent update on a further 45 patients (20).

The relative rarity of insulinomas means that the majority are diagnosed and managed in tertiary referral centres. The organisation of randomised controlled trials in such a small patient pool is notoriously difficult. It therefore remains instructive to review the information collected by centres with the experience of managing these rare neuroendocrine tumours. Recent case series have been published examining both secular trends in the presentation and diagnosis of insulinoma (21), and have also reported on the relative accuracy of several methods of localisation (20). We have previously reported that MRI appears to be increasingly accurate in demonstrating small pancreatic tumours (22). These single-centre series provide useful information, and we thought it useful to review data for diagnosis and localisation of insulinoma from our centre, and to learn lessons from any important similarities and differences.

Subjects and methods

We carried out a retrospective review as a single-centre study (audit number 08/85 Barts and the London NHS Trust).

Subjects

For evaluation of diagnostic data, inclusion criteria were all patients with a biochemical diagnosis felt to be consistent with insulinoma (or with sufficiently persuasive data accompanied by a very suspicious clinical history). For analysis of localisation of insulinoma, the patient group was restricted to those who had been treated surgically and in whom subsequent histological analysis confirmed an insulinoma. We reviewed data from all eligible patients presenting to our centre over a 19-year period (1990–2009). We included patients with tumour-prone syndromes such as multiple endocrine neoplasia (MEN-1 and MEN-2) syndromes, in whom the relevant data were available, but as the natural history and more intensive surveillance protocols in these patients affect the diagnostic and treatment pathways, many did not fulfil the eligibility criteria. Patients included were those with a biochemical diagnosis of insulinoma, in whom localisation studies were carried out in order to direct the surgical approach.

Biochemical diagnosis

Case notes were reviewed for demographic characteristics, clinical history and relevant biochemical investigations. Symptomatic hypoglycaemia ($\leq$2.2 mmol/l) together with elevated plasma insulin and elevated C-peptide levels was confirmed by means of a prolonged supervised fast and/or a glucose tolerance test (this has more recently been replaced by a mixed-meal test). Other causes of hypoglycaemia were excluded by the usual methods.

Anatomical localisation

Non-invasive localisation investigations included CT and MRI. CT imaging was performed using an eight-slice multidetector CT scanner (Lightspeed ultra, GE Healthcare, Chalfont St Giles, Buckinghamshire, UK) but several earlier studies were performed on a single-slice CT scanner (HiSpeed ZXi GE Healthcare). The imaging protocols were not standardised. Institutional protocols were revised over the period of data collection, and

Downloaded from Bioscientifica.com at 04/02/2019 12:59:21PM
via free access

external studies sent into the hospital were imaged with variable protocols. Where possible, i.v. contrast had been administered and a triple-phase CT scan of the pancreas was obtained. At our institution, MRI was performed using a 1.5 Tesla scanner (early studies on GE and later studies on Phillips scanners). The routine MR sequences including axial T1-weighted, axial T2-weighted and axial T1-weighted images with fat saturation before and after i.v. administration of gadolinium were obtained. The choice of imaging modalities was pragmatic and dependent on the quality of available studies, but the majority of patients had both investigations. The images were routinely reviewed in a joint radiology/endocrinology clinical meeting. Octreotide imaging was performed in the Nuclear Medicine Department using ^{111}Indium-octreotide (Octreoscan), and the results were reviewed in a multidisciplinary team meeting including a Nuclear Medicine specialist. The use of the technique was physician dependent but not limited to patients with non-diagnostic cross-sectional imaging. Non-functional semi-invasive studies were provided by EUS, carried out in the majority of patients regardless of results from cross-sectional imaging, due to the surgeon's preference for additional anatomical information.

Functional localisation

Selective arteriography and calcium arterial stimulation with hepatic venous sampling for insulinoma localisation used a technique adapted from the original description (19). This technique was used in as many patients as feasible, regardless of the results of cross-sectional imaging, to provide a corroborative functional perspective. After an overnight fast, non-ionic contrast was injected into the main branches of arterial supply to the pancreas, namely the common hepatic artery (CHA), gastroduodenal artery (GDA), superior mesenteric artery (SMA) and splenic artery. Intravenous 5% dextrose was used if required to maintain blood glucose above the hypoglycaemic range, but use was kept to a minimum to ensure the lowest possible background stimulation of insulin secretion. After each selective arteriogram, 10% calcium gluconate diluted to a volume of 10 ml with normal saline was injected into the artery at a dose of 0.00125 mmol Ca^{++} per kg body weight (0.025 mEq/kg). Based on CT/MRI, the artery most likely to supply the lesion seen on imaging was injected last. Samples were obtained before and at intervals after calcium injection from the hepatic vein and kept on ice until they could be centrifuged, and the resulting plasma was stored at -20 °C.

To interpret the calcium stimulation test, an increase in insulin concentration from baseline to peak of twofold or greater constituted a positive response (19). In the absence of anatomic variants, a positive response following injection into the CHA, GDA or SMA predicts a lesion in the head of the pancreas, while a positive response following splenic artery injection predicts a lesion in the body or tail of the pancreas. When a positive response was seen in more than one artery, by convention, the territory with the greatest insulin rise was used to predict regionalisation (20).

Surgical localisation and pathological examination

Surgery was planned and carried out on the basis of the results of all the investigations in combination. In cases where there was doubt, full pancreatic exploration was carried out at surgery including the use of intra-operative ultrasound. Localisation of the lesion at the time of surgery was noted. The diagnosis was made on routine haematoxylin and eosin staining and confirmed immunohistochemically with neuroendocrine markers including CD56 and chromogranin. Pancreatic hormonal expression was always tested, but the presence or absence of insulin was not considered to be a diagnostic feature. Features such as tumour size and number of mitoses (as a surrogate for malignant potential) were evaluated by a departmental pathologist. The anatomical and functional localisation techniques were compared to the intra-operative findings.

Data analysis

The basic data were analysed using descriptive statistics and are presented as means $\pm$ S.D.

Results

Overall demographics and patient flow

Thirty-seven instances of biochemically or histologically likely insulinoma were identified in 36 different patients. Of these, 32 cases were thought to be sporadic (one insulinoma being a recurrence following biochemical cure), while a further five were detected in patients with MEN-1, either diagnosed prior to the insulinoma or concurrent with the diagnosis (Table 1). Data obtained in our institution were included in the analysis, as were any investigation results re-evaluated at our centre (for example, imaging performed elsewhere but reviewed at our centre).

Diagnostic data Data were available on a total of 34 prolonged supervised fasts, while a further three were diagnosed serendipitously. Of the 23 patients with data available regarding the length of the prolonged fast, one patient had a negative prolonged supervised fast, and was diagnosed on the basis of reactive hypoglycaemia with inappropriately elevated insulin: 12 fasts (52.2%) were terminated within 24 h, 10 (43.5%) between 24 and 48 h, and none between 48 and 72 h. All patients achieved a blood glucose <2.2 mmol/l in order to fulfil

Downloaded from Bioscientifica.com at 04/02/2019 12:59:21PM
via free access

Table 1 Demographic, prolonged fast and insulinoma data for cohort.

	Number	Percentage of cases in category
Demographic data (n=37)		
Males	14	37.8
Females	23	62.2
Sporadic	32	86.5
MEN-1	5	13.5
Medically managed	7	18.9
Surgically managed	30	81.1
Metastatic at diagnosis	2	5.4
Data from prolonged supervised fast (n=34)		
Glucose ≤2.2 mmol/l	33 (one negative fast)	97.1
Time to hypo (n=23)		
Time to hypo <24 h	12	52.2
Time to hypo 24–48 h	10	43.5
Time to hypo >48 h	0 (one negative fast)	0
C-peptide >220 pmol/l (n=24)	23	95.8
Surgical localisation data (n=30)		
Head/uncinate process	15	50.0
Body/tail	14 (plus one at junction of neck/body)	46.7
Surgical size (n=26)		
<1 cm	3	11.5
1–2 cm	18	69.2
>2 cm	5	19.2

the diagnostic criterion (Table 1). The mean glucose at the termination of the fast was 1.7±0.3 mmol/l. Thirty-two of the patients had a diagnostic level of insulin >3.0 IU/l, with one further borderline result and one in whom hypoglycaemia was never achieved during the prolonged fast (and who was diagnosed on the biochemical data from a meal test). The mean C-peptide at the time of hypoglycaemia was 1044.6 pmol/l with a range of 297–3215 pmol/l. Chromogranin A was available for 12 of the cases and was elevated above the upper limit of the normal range on at least one occasion in nine of the subjects (75%).

Demographics

Of the 37 cases of insulinoma, seven were managed medically. These cases were excluded from the analysis of the accuracy of localisation techniques as they lack surgical and histological corroboration. One of these was a patient with MEN-1 who presented with widespread metastases. Of the sporadic cases, one was excluded because presentation with widespread metastases prompted a non-surgical approach, while one was managed with diazoxide when open laparotomy, even with the aid of intra-operative ultrasound, failed to localise the lesion (the exclusion being based on the absence of surgical localisation against which to measure the accuracy of the investigations); the remaining four were managed medically because of a combination of uncertainties in localisation and patient preference for, and tolerance of, conservative treatment.

Of the 37 cases, 36 were first presentations, while one was a recurrence after surgery: 23 were in females and 14 were in males (including the recurrence), and the mean age at the time of biochemical diagnosis was 46.0±13.7 years, with a range of 20.9–77.3 years.

Tumour information

Thirty cases were treated with surgical resection at open operation. Overall, 13 patients underwent enucleation of the tumour, nine patients underwent distal pancreatectomy, six had pylorus-sparing pancreaticoduodenectomy, and two had other or combined procedures: 20 of the patients had intra-operative ultrasound at the time of surgery. Data on tumour size were available for 26 of these tumours. Three of the tumours were <1 cm in diameter (11.5%), 18 were 1–2 cm (69.2%) and 5 were >2 cm (19.2%). Fifteen were found in the head or uncinate process (50%), while 14 were found in the body or tail of the pancreas (43.3%) and one was found at the junction of the neck and body (Table 1). Immunohistochemistry was available for 28 of the cases, with the stains applied varying slightly over time. Ninety-six percent of these were positive or weakly positive for insulin (Table 2), and there was variable weak positivity for other pancreatic hormones such as glucagon and pancreatic polypeptide (PP). The degree of malignant potential as expressed by the Ki-67 score was determined in 16 of the cases: it was expressed as <1% in eight cases, <2% in four cases, and in two cases with each of Ki-67 <5 and <20%.

Localisation data

Cross-sectional imaging Cross-sectional imaging was available in all 30 cases, either performed prior to referral and reviewed at our centre, or carried out during the process of investigation. Twenty-five patients had CT imaging (83.3%), while 28 had MRI (93.3%), and therefore, 24 (80%) patients underwent both types

Table 2 Immunohistochemistry for the histological samples after operation to surgically remove an insulinoma (n=28) and the number of samples with the listed finding.

Marker	Positive	Weak positive	Negative	No record
MNF116	10	0	0	18
Synaptophysin	11	0	0	17
CD56	12	1	1	14
Chromogranin	19	0	0	9
Insulin	19	8	1	0

Downloaded from Bioscientifica.com at 04/02/2019 12:59:21PM
via free access

of imaging. A pragmatic approach was adopted depending on quality and localisation information that could be obtained from prior imaging. Of the CT scans carried out, 16 correctly localised the lesion (64%), while eight CT scans (32%) failed to localise the lesion; one scan gave a confusing result with two lesions identified. Of the MRI scans carried out, 21 correctly localised the lesion (75%) (example shown in Fig. 1). Four MRI scans (14.2%) failed to localise the lesion. However, three scans localised a lesion incorrectly. The pragmatic use of the combined approach ensured that 24 of the lesions were localised correctly by imaging prior to surgery (80%). Five lesions were correctly localised on MRI but were not visible on CT scanning, while two lesions were visible on CT scanning but not detected by MRI (although in one of these cases, MRI localisation was hampered by marked gut-movement artefact).

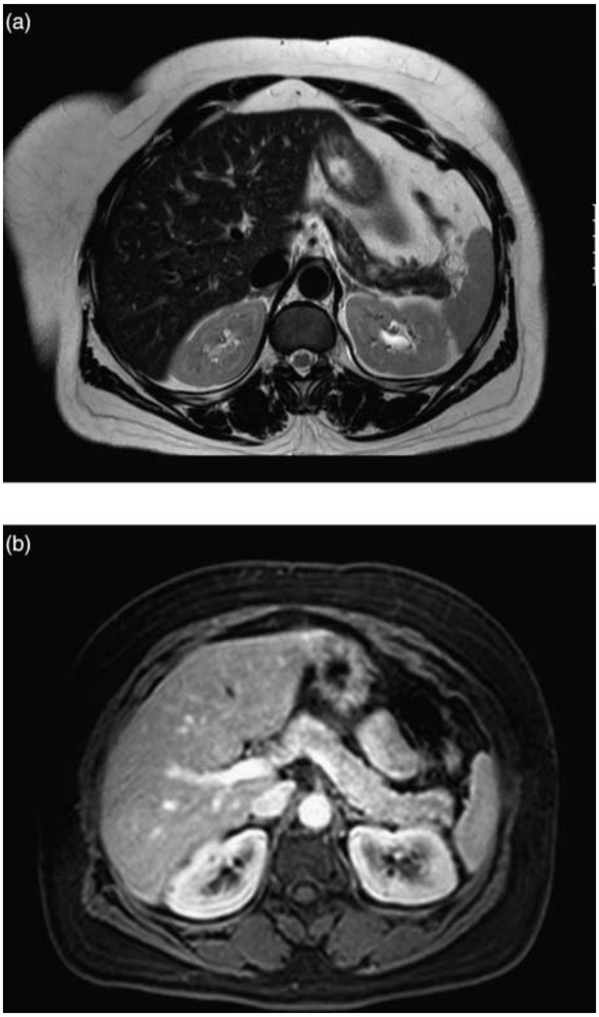

Figure 1 Example of the use of MRI in the localisation of insulinoma. (a) T2-weighted image demonstrating a lesion in the tail of the pancreas, less well seen in (b) T1-weighted MRI demonstrating the pancreas in the same patient.

Re-analysis of the data, to include cases which were biochemically diagnosed but not histologically proven, also encompasses cases that were managed medically. The rationale for this is that the absence of localisation is likely to have played a part in the withholding of surgery. Excluding from this the patients with widespread metastases at the time of diagnosis (for whom localisation was evident), the data demonstrated for CT a corrected localisation accuracy of 16 cases out of 28 (57%) and for MRI a corrected detection rate of 21 cases out of 31 patients (66%).

Nuclear medicine Twenty patients had radiolabelled octreotide scans performed. Of these, ten scans (50%) were negative (50%) and ten scans (50%) were positive. Five of the positive scans localised a lesion concordant with the final surgical location, while five were not useful predictors of the final surgical location. Octreotide scanning had been carried out in three of the four cases in which cross-sectional imaging (CT and MRI) was unable to localise the lesion, and in none of these cases did the nuclear medicine imaging add further diagnostic information.

Endoscopic ultrasound Data were available on a total of 26 EUS scans performed to localise the insulinoma. Where cross-sectional imaging was also available, the results would have been available to the endoscopist prior to the procedure. Seventeen of these procedures correctly localised a lesion (65.3%) but in five cases no lesion was seen (19.2%); in addition, there were a further four cases in which a lesion was correctly identified but in which a further lesion was also noted, not subsequently confirmed at operation (false positives), resulting in some confusion (15.3%). EUS had been carried out in three of the four cases in which cross-sectional imaging (CT and MRI) was unable to localise the lesion. In one of these cases, EUS was also negative; in one case, the EUS demonstrated a lesion concordant with the final surgical localisation, and in one case, the EUS demonstrated a lesion that was in a pancreatic region discordant with the final surgical localisation.

Calcium stimulation catheter Twenty-seven patients had calcium stimulation testing, although two of these were incomplete as it was not technically possible to cannulate one of the arteries. Of the studies, six gave a non-regionalising response to calcium injection (22.2%); 17 of the findings were concordant with the final histological localisation (62.9%). However, four studies (14.8%) gave discordant results with the final regionalisation of the lesion being different to the territory of insulin rise or unhelpful. No obvious technical difficulty with these particular studies was noted.

For the six patients in whom imaging failed to confirm localisation of the insulinoma, in three cases calcium stimulation provided additional helpful

Downloaded from Bioscientifica.com at 04/02/2019 12:59:21PM
via free access

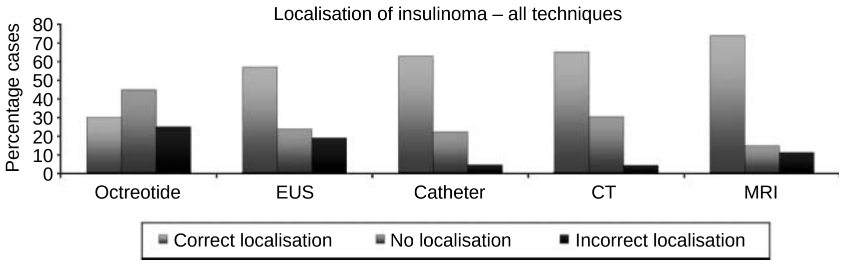

Figure 2 Summary of localisation accuracy for insulinoma in our study population (*n*=30 treated surgically).

information for regionalisation. In three further cases, the calcium stimulation test did not provide additional value, or indeed confused the situation further. Localisation information is summarised in Fig. 2. No significant adverse events were recorded as a consequence of calcium stimulation testing.

Discussion

The classical diagnostic test for insulinoma has been provocation of hypoglycaemia by a supervised fast which may last up to 72 h. However, there is increasing economic pressure to demonstrate Whipple's triad without a 3-day admission, and reports from some centres have suggested that up to 83% of patients with subsequent insulinoma diagnoses are hypoglycaemic within the first 22 h of the fast, enabling the procedure to be carried out under out-patient supervision (21). The majority of our patient cohort required a fast >24 h; this may reflect in part the less stringent criteria for termination of the fast applied in other cohorts, such as 3.3 and 2.75 mmol/l (21). For the cohort of patients, increasingly recognised, with strong post-prandial symptoms but a negative fast (around 5% of patients), a mixed-meal test does appear to be the most physiological investigation (9).

Once the biochemical diagnosis of a probable insulinoma has been established, localisation investigations can greatly aid management decisions. Non-invasive methods such as abdominal ultrasound, CT scanning and MRI have the advantage of being simple and quick to perform with few potential complications. However, non-functional pancreatic nodules may occur incidentally, and these methods do not corroborate structural abnormality with hormone secretion (23). Several series have reviewed the accuracy of these methods for localising the lesion; for example, in a recent series of 237 patients, the accuracy of CT was 55% and of MRI was 42% in tumour localisation (21). In another series of 39 sporadic cases of insulinoma, the accuracy of CT scanning was 35% with a false negative rate of 49% and a false positive localisation rate of 16%. For MRI, the accuracy was 30% with a false negative rate of 57% and a false positive rate of 13% (20). While some small radiological series have suggested greater sensitivity for both CT and MRI (13, 15), in general, the low accuracy of cross-sectional imaging has in part driven the development of more invasive methods. The differences in the series may relate to the relatively small size of many insulinomas at the time of diagnosis. Indeed, in our series, nearly 80% of histologically proven tumours were <2 cm in diameter, broadly compatible with other series. Interestingly, however, in our group of patients, the accuracy of cross-sectional imaging for localisation was higher, with 65% accuracy for CT and 74% for MRI. This is comparable to another recent small UK series of 20 patients (10), in which the sensitivity of MRI was noted to be 71%. However, in this study, when the accuracy of localisation by this method was evaluated, the accuracy was only 56%. In a further UK series of 28 patients, the tumour detection rate for MRI was also 71% (12). In our series, when both CT and MR modalities were used together as part of a pragmatic approach in which almost all patients with a sporadic tumour were investigated, 80% of patients with a confirmed histological diagnosis had their insulinoma correctly localised.

EUS was introduced as a more invasive procedure which, while also being an anatomical rather than a functional technique, with the advent of biopsy via the endoscope can in some cases provide histological information. The accuracy in one large series of insulinoma patients was calculated to be 75% (21). However, this modality is highly operator dependent. In our series, 80.7% of the tumours were localised. This included 13.3% of the total who had confusing results in that in addition to the lesion correctly identified, a further lesion was incorrectly noted elsewhere in the pancreas. In addition, there were 24% false negative localisations. In the case of radiolabelled octreotide scanning, the octreotide uptake prevalence of 50% is less operator dependent, but instead the low localisation rate is contingent upon the majority of tumours lacking a high density of somatostatin receptors. Our findings are concordant with most of the published literature (24). The relative frequency of confusing results could suggest that this is a tool best kept in reserve for cases in which localisation is proving difficult, and requires the maximum possible weight of evidence, but even in such cases in our series, octreotide scanning added little in terms of localisation. More recent literature suggests

Downloaded from Bioscientifica.com at 04/02/2019 12:59:21PM
via free access

additional sensitivity with the use of ^{18}F-DOPA PET scanning (25), while preliminary evidence suggests a role for scanning with a radiolabelled GLP-1 analogue exendin (26, 27).

Selective pancreatic intra-arterial calcium injections to localise islet cell tumours were introduced in the 1980s (18). The success of the investigation relies on several assumptions, including that the tumour will have a dominant arterial supply, that calcium evokes a characteristic response of coordinated discharge of vesicles from the entire syncytium of tumour cells, and that the remainder of the β-cells will be suppressed from the chronic hormone hypersecretion. As well as the theoretical advantage of corroboration of structure and function, accuracy rates are reported to be very high, with figures of 93% (21) and 89% (20) being quoted. However, it should be pointed out that to be accurate this is regionalisation rather than true localisation. A number of series have reported the additional value of this test in localisation, particularly in cases where non-invasive methods have been unsuccessful (12) Our series provides some cautionary evidence that accuracy is not always 100%, and given that the procedure is not without risk, there may be merit in reserving this investigation for cases where non-invasive localisation results have been contentious. Given the relatively high accuracy for MRI in our series, prospective studies evaluating radiological confidence in the findings against the final outcome may allow prediction of which patients require invasive investigations in addition. This may in turn reduce the radiation exposure to the patients and also the overall cost of the diagnostic episode (10).

The results from the MEN patients are difficult to compare due to the small numbers within the cohort. In general, many such patients present with incidental findings of pancreatic nodules and undergo one or more prior pancreatic resections, making further preoperative localisation strategies challenging. For this group of patients, the conclusions regarding insulinoma localisation in sporadic cases are unlikely to be suitable for extrapolation.

In summary, while the diagnosis of an insulinoma has become increasingly consensual, and diagnostic algorithms are well established, preoperative localisation remains difficult. Individual institutions have developed particular expertise in specific techniques, such as EUS, but these may not readily transfer to other centres where such techniques are less commonly used. We and others have increasingly come to rely on the calcium stimulation catheter technique as the final arbiter, and indeed, it is one of the most useful means of functional regionalisation. However, our present series demonstrates that it too is not always accurate, and we have been impressed at the increasing usefulness of cross-sectional imaging, especially recent trends in MRI. We suggest that increasing confidence with MRI, novel sequences including diffusion-weighted imaging and novel software programs, will lead to this technique being used as an anatomical localisation technique complementary to functional imaging with calcium stimulation.

Declaration of interest

The authors declare that there is no conflict of interest that could be perceived as prejudicing the impartiality of the research reported.

Funding

This research did not receive any specific grant from any funding agency in the public, commercial or not-for-profit sector.

References

1 Service FJ. Diagnostic approach to adults with hypoglycemic disorders. *Endocrinology and Metabolism Clinics of North America* 1999 **28** 519–532, vi.
2 Service FJ, McMahon MM, O'Brien PC & Ballard DJ. Functioning insulinoma – incidence, recurrence, and long-term survival of patients: a 60-year study. *Mayo Clinic Proceedings* 1991 **66** 711–719.
3 Service FJ & Natt N. The prolonged fast. *Journal of Clinical Endocrinology and Metabolism* 2000 **85** 3973–3974.
4 Service FJ, Dale AJ, Elveback LR & Jiang NS. Insulinoma: clinical and diagnostic features of 60 consecutive cases. *Mayo Clinic Proceedings* 1976 **51** 417–429.
5 Service FJ. Hypoglycemic disorders. *New England Journal of Medicine* 1995 **332** 1144–1152.
6 Vezzosi D, Bennet A, Fauvel J, Boulanger C, Tazi O, Louvet JP & Caron P. Insulin levels measured with an insulin-specific assay in patients with fasting hypoglycaemia related to endogenous hyperinsulinism. *European Journal of Endocrinology* 2003 **149** 413–419.
7 Chia CW & Saudek CD. The diagnosis of fasting hypoglycemia due to an islet-cell tumour obscured by a highly specific insulin assay. *Journal of Clinical Endocrinology and Metabolism* 2003 **88** 1464–1467.
8 Vezzosi D, Bennet A, Fauvel J & Caron P. Insulin, C-peptide and proinsulin for the biochemical diagnosis of hypoglycaemia related to endogenous hyperinsulinism. *European Journal of Endocrinology* 2007 **157** 75–83.
9 Cryer PE, Axelrod L, Grossman AB, Heller SR, Montori VM, Seaquist ER, Service FJ & Endocrine Society. Evaluation and management of adult hypoglycemic disorders: an Endocrine Society Clinical Practice Guideline. *Journal of Clinical Endocrinology and Metabolism* 2008 **94** 709–728.
10 Ravi K & Britton BJ. Surgical approach to insulinomas: are preoperative localisation tests necessary? *Annals of the Royal College of Surgeons of England* 2007 **89** 212–217.
11 Pasieka JL, McLeod MK, Thompson NW & Burney RE. Surgical approach to insulinomas. Assessing the need for preoperative localisation. *Archives of Surgery* 1992 **127** 442–447.
12 Morganstein DL, Lewis DH, Jackson J, Isla A, Lynn J, Devendra D, Meeran K & Todd JF. The role of arterial stimulation and simultaneous venous sampling in addition to cross-sectional imaging for localisation of biochemically proven insulinoma. *European Journal of Radiology* 2009 **19** 2467–2473.
13 Gouya H, Vignaux O, Augui J, Dousset B, Palazzo L, Louvel A, Chaussade S & Legmann P. CT, endoscopic sonography, and a combined protocol for preoperative evaluation of pancreatic insulinomas. *AJR. American Journal of Roentgenology* 2003 **181** 987–992.

Downloaded from Bioscientifica.com at 04/02/2019 12:59:21PM
via free access

14 Ichikawa T, Peterson MS, Federle MP, Baron RL, Haradome H, Kawamori Y, Nawano S & Araki T. Islet cell tumour of the pancreas: biphasic CT versus MR imaging in tumour detection. *Radiology* 2000 **216** 163–171.
15 Thoeni RF, Mueller-Lisse UG, Chan R, Do NK & Shyn PB. Detection of small, functional islet cell tumours in the pancreas: selection of MR imaging sequences for optimal sensitivity. *Radiology* 2000 **214** 483–490.
16 McLean AM & Fairclough PD. Endoscopic ultrasound in the localisation of pancreatic islet cell tumours. *Best Practice & Research. Clinical Endocrinology & Metabolism* 2005 **19** 177–193.
17 Chang F, Chandra A, Culora G, Mahadeva U, Meenan J & Herbert A. Cytologic diagnosis of pancreatic endocrine tumours by endoscopic ultrasound-guided fine-needle aspiration: a review. *Diagnostic Cytopathology* 2006 **34** 649–658.
18 Doppman JL, Miller DL, Chang R, Shawker TH, Gorden P & Norton JA. Insulinomas: localisation with selective intraarterial injection of calcium. *Radiology* 1991 **178** 237–241.
19 Doppman JL, Chang R, Fraker DL, Norton JA, Alexander HR, Miller DL, Collier E, Skarulis MC & Gorden P. Localisation of insulinomas to regions of the pancreas by intra-arterial stimulation with calcium. *Annals of Internal Medicine* 1995 **123** 269–273.
20 Guettier JM, Kam A, Chang R, Skarulis MC, Cochran C, Alexander HR, Libutti SK, Pingpank JF & Gorden P. Localisation of insulinomas to regions of the pancreas by intraarterial calcium stimulation: the NIH experience. *Journal of Clinical Endocrinology and Metabolism* 2009 **94** 1074–1080.
21 Placzkowski KA, Vella A, Thompson GB, Grant CS, Reading CC, Charboneau JW, Andrews JC, Lloyd RV & Service FJ. Secular trends in the presentation and management of functioning insulinoma at the Mayo Clinic, 1987–2007. *Journal of Clinical Endocrinology and Metabolism* 2009 **94** 1069–1073.
22 Owen NJ, Sohaib SA, Peppercorn PD, Monson JP, Grossman AB, Besser GM & Reznek RH. MRI of pancreatic neuroendocrine tumours. *British Journal of Radiology* 2001 **74** 968–973.
23 Service FJ. Classification of hypoglycemic disorders. *Endocrinology and Metabolism Clinics of North America* 1999 **28** 501–517, vi.
24 Virgolini I, Traub-Weidinger T & Decristoforo C. Nuclear medicine in the detection and management of pancreatic islet-cell tumours. *Best Practice & Research. Clinical Endocrinology & Metabolism* 2005 **19** 213–227.
25 Kauhanen S, Seppanen M, Minn H, Gullichsen R, Salonen A, Alanen K, Parkkola R, Solin O, Bergman J, Sane T, Salmi J, Valimaki M & Nuutila P. Fluorine-18-L-dihydroxyphenylalanine (^{18}F-DOPA) positron emission tomography as a tool to localize an insulinoma or beta-cell hyperplasia in adult patients. *Journal of Clinical Endocrinology and Metabolism* 2007 **92** 1237–1244.
26 Wild D, Macke H, Christ E, Gloor B & Reubi JC. Glucagon-like peptide 1-receptor scans to localize occult insulinomas. *New England Journal of Medicine* 2008 **359** 766–768.
27 Christ E, Wild D, Forrer F, Brändle M, Sahli R, Clerici T, Gloor B, Martius F, Maecke H & Reubi JC. Glucagon-like peptide-1 receptor imaging for localisation of insulinomas. *Journal of Clinical Endocrinology and Metabolism* 2009 **94** 4398–4405.

Received 24 February 2010
Accepted 4 March 2010

Downloaded from Bioscientifica.com at 04/02/2019 12:59:21PM
via free access
