# Supplementary material for: Diagnostic value of ASVS for insulinoma localization: A systematic review and meta-analysis
Source: PLoS One. 2019 Nov 19;14(11):e0224928. doi: 10.1371/journal.pone.0224928 (PMC6863549; doi:10.1371/journal.pone.0224928)
Supplement: S2 File — (ZIP) [file pone.0224928.s002.zip › included studies/Insulin determination by specific and unspecific immunoassays in patients with insulinoma evaluated by the arterial stimulation and venous sampling test.pdf]

European Journal of Endocrinology (2004) **151** 123–126 ISSN 0804-4643

CLINICAL STUDY

Insulin determination by specific and unspecific immunoassays in patients with insulinoma evaluated by the arterial stimulation and venous sampling test

Peter Wiesli, Michael Brändle, Thomas Pfammatter[1], Jürgen Zapf, Giatgen A Spinas and Christoph Schmid

Department of Internal Medicine, Division of Endocrinology and Diabetes and [1] Department of Radiology, Institute for Diagnostic Radiology, University Hospital of Zurich, CH-8091 Zurich, Switzerland

(Correspondence should be addressed to Peter Wiesli; Email: peter.wiesli@DIM.usz.ch)

Abstract

Objective: The aim of this study was to compare insulin concentrations measured by a traditional radioimmunoassay (RIA) and a more specific enzyme-linked immunosorbent assay (ELISA) in blood samples obtained during the arterial stimulation and venous sampling (ASVS) test in patients with insulinoma.

Design: Prospective case series.

Methods: In 14 patients with an insulinoma undergoing an ASVS test, blood samples were obtained from the hepatic vein at baseline and 60 s after calcium injection into an artery supplying the tumour and a control artery (supplying pancreatic tissue without tumour). A selective arterial calcium stimulation was performed in five additional patients without evidence for an insulinoma. We measured insulin by a traditional RIA and a specific immunoassay.

Results: In patients with insulinoma, insulin concentrations increased between 2.3- and 24.2-fold (median 8.2-fold) when measured by RIA and between 7.3- and 59.4-fold (median 16) when measured by ELISA following calcium injection into the artery supplying the tumour. Following calcium injection into the control artery, insulin concentrations were 0.6 to 1.3 times (median 1.0) the baseline values by RIA and 0.5 to 2.5 times (median 1.1) the baseline values by ELISA. In patients without insulinoma, insulin concentrations increased following calcium stimulation between 0.7- and 2.1-fold (median 1.3-fold) when measured by RIA and between 0.6- and 4.7-fold (median 1.3-fold) when measured by ELISA.

Conclusions: When insulin is measured by specific immunoassays, a higher cut-off (i.e. five- to sixfold increase) rather than the traditional criterion of a twofold increase should be used to localise an insulinoma during the ASVS test. The increase in insulin concentrations following calcium stimulation is significantly higher when insulin is measured by a specific assay compared with results obtained with traditional RIAs.

European Journal of Endocrinology **151** 123–126

Introduction

The diagnosis of an insulinoma is established by demonstrating endogenous hyperinsulinaemic hypoglycaemia (1). Since islet-cell tumours may secrete high concentrations of proinsulin in the fasting state, insulin concentrations measured by specific immunoassays may be markedly lower than insulin levels obtained by traditional radioimmunoassays (RIAs) which exhibit considerable cross-reactivity with proinsulin (2).

Selective arterial calcium stimulation and hepatic venous sampling (ASVS) is a powerful method for the localisation of insulin-secreting tumours (3, 4). Since calcium administration elicits a significant increase in insulin secretion by insulinomas but not by normal β-cells, a significant (more than twofold) rise in insulin levels in the hepatic vein after the injection of calcium into the pancreatic arteries indicates the presence of an insulin-secreting tumour in the vascular territory of the artery stimulated. The traditional criterion of a greater than twofold increase in insulin concentration has been established by Doppman and co-workers (3). However, the methodology of insulin measurement by specific or traditional RIAs has never been addressed or considered as a critical issue for the accurate localisation of insulinoma. In most original data concerning ASVS, the methodology of the insulin determination has not even been mentioned (3, 5–9). Since islet-cell tumours may secrete considerable amounts of proinsulin in the fasting state, a marked release of proinsulin by

© 2004 Society of the European Journal of Endocrinology Online version via http://www.eje.org

Downloaded from Bioscientifica.com at 05/19/2019 03:47:35AM
via free access

calcium stimulation could also occur and thereby affect the results of ASVS, depending on the methodology used for the determination of insulin. To the best of our knowledge, the release of proinsulin by local intra-arterial calcium administration has not been investigated systematically. Only Shimizu and co-workers described the release of proinsulin during ASVS in two patients with an insulinoma (2).

The aim of this study was to assess and compare insulin concentrations measured by a traditional RIA and a more specific enzyme-linked immunosorbent assay (ELISA) as well as proinsulin concentrations in blood samples obtained during ASVS.

Subjects and methods

Patients

Fourteen patients with documented fasting hypoglycaemia who underwent ASVS between June 1999 and March 2003 and with a histologically confirmed diagnosis of an insulinoma were included in this study. All patients had a single insulinoma and were cured by surgery. Selective arterial calcium stimulation of pancreatic arteries was performed in five additional patients without evidence for an insulinoma undergoing celiac angiography. Two patients underwent the angiography for the evaluation of an inactive neuroendocrine tumour (histologically verified; immunohistochemistry for insulin negative) and one patient because of suspected pancreatic lesion in the computed tomography scan (which was not confirmed by magnetic resonance imaging and clinical follow-up). One patient underwent an ASVS test following successful pancreatic resection of an insulin-secreting tumour because a micrometastasis was found in the peripancreatic adipose tissue. In these four patients, absence of insulin-secreting tumour cells was demonstrated by a negative 72-h fast. One woman without clinical evidence for hypoglycaemic episodes underwent the selective arterial calcium stimulation test to localise a gastrinoma. Written informed consent for ASVS was obtained from all patients.

Selective arterial calcium stimulation with hepatic venous sampling (ASVS)

The procedure was performed as previously described (10). Glucose 5% was infused through a peripheral vein during the test to prevent hypoglycaemia. A sampling catheter was placed transfemorally in the right hepatic vein close to its junction with the inferior vena cava. After a standard angiography, the proper hepatic, the gastroduodenal, the splenic, and the superior mesenteric arteries were catheterised. Each artery was stimulated with calcium gluconate (0.025 milliequivalents Ca^{++} per kg body weight). Blood was sampled from the right hepatic vein before (= 0) and 30, 60 to 120 s after the intra-arterial calcium injection. At least 5 min were left between each calcium injection. Insulin (measured by RIA) was determined in all samples. A more than twofold rise in insulin within 30–120 s after the injection of calcium indicated an insulin-secreting tumour in the vascular territory of the artery stimulated (in contrast to no response from normal β-cells). The additional determinations of insulin by a specific immunoassay (ELISA) and proinsulin were performed in the samples obtained following calcium stimulation in the artery supplying the tumour (indicated by the maximum increase of insulin concentrations) and a control artery (no increase in insulin concentrations). From each artery, the baseline sample and the sample with the highest insulin concentration following the calcium injection were selected for the additional determinations.

Laboratory investigations

Insulin was measured by RIA and a specific immunoassay (ELISA). As traditional assay, we used a solid-phase RIA (CIS Bio international, Oris Industries, Gif-Sur-Yvette, France) with a cross-reactivity of 14% for proinsulin and 0.1% for C-peptide; the normal overnight fasting range provided by the manufacturer was 30 to 138 pmol/l, the lower limit of detection was 14 pmol/l. As specific insulin assay, we used a two-site enzyme-linked immunosorbent assay (DAKO Ltd, Cambridgshire, UK) with a cross-reactivity of 0.3% for proinsulin and no cross-reactivity for C-peptide. Reference interval (95% confidence interval) in healthy individuals provided by the manufacturer was 11 to 86 pmol/l, the lower limit of detection was 3 pmol/l. Proinsulin was measured with a two-site enzyme-linked immunosorbent assay (DAKO Ltd) without cross-reactivity for insulin. The reference interval provided by the manufacturer in healthy individuals was 2 to 6 pmol/l.

Results

Patients with an insulinoma (n = 14)

In the 14 patients (11 women, 3 men) with an insulinoma, the median age was 55 (range 33–77) years and the body mass index was 25 (range 20–34) kg/m^2. ASVS was performed in all patients because of documented fasting hyperinsulinaemic hypoglycaemia. Localisation of insulinoma with ASVS was accurate in all patients included in this study, i.e. insulin concentrations (by RIA) in the hepatic vein increased twofold or more of the baseline value following calcium injection into the artery supplying the tumour. The diagnosis of an insulinoma was confirmed histologically in all patients. The gradients of insulin following calcium stimulation are presented as multiples of the baseline values; all gradients are shown in Fig. 1. Blood glucose

Downloaded from Bioscientifica.com at 05/19/2019 03:47:35AM
via free access

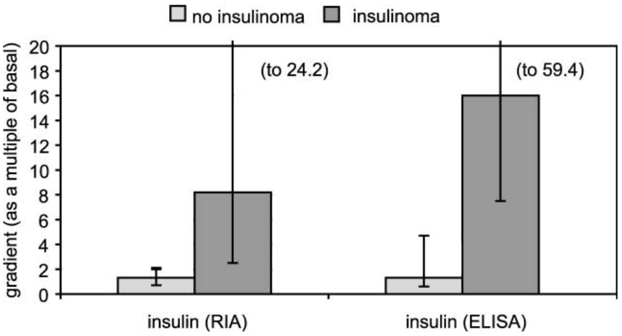

Figure 1 Insulin gradients during ASVS. Median increase (and range) in insulin concentrations in the hepatic vein following calcium injection into the artery supplying the tumour in 14 patients with an insulinoma and following calcium injection into pancreas-supplying arteries in five patients without evidence for an insulinoma. Gradients are shown as multiples of the baseline concentrations. A twofold increase in insulin concentrations is diagnostic for an insulinoma when insulin is measured by an unspecific RIA. A higher cut-off should be requested when insulin is determined by a specific immunoassay.

Table 1 Patients without insulinoma. Insulin concentrations measured by an unspecific radioimmunoassay (RIA) and a specific immunoassay (ELISA) in the hepatic vein at baseline and after calcium injection into arteries supplying pancreatic tissue. Gradients represent multiples of the baseline concentrations. In patient 1, three arteries were stimulated, in patients 2, 3 and 4 two arteries, in patient 5 one artery.

	**Insulin by RIA** (pmol/l)			**Insulin by ELISA** (pmol/l)		
**Patient**	Basal	Stimulated	Gradient	Basal	Stimulated	Gradient
1	57	84	1.5	4.2	13.3	3.2
	49	75	1.5	2.3	11	4.7
	85	65	0.8	7.9	8.5	1.1
2	130	93	0.7	35.7	37.3	1
	184	141	0.8	87	47.9	0.6
3	213	228	1.1	28.7	82.2	2.9
	95	177	1.9	92	87.5	1
4	184	300	1.6	136.4	202.4	1.5
	461	434	0.9	216.9	235.9	1.1
5	34	70	2.1	6	18.4	3.1
Median	113	117	1.3	32	43	1.3

concentrations during the ASVS test were 4.7 (range 4–7.2) mmol/l.

Median insulin gradients by RIA following calcium injection into the control artery ranged from 0.6- to 1.3-fold (median 1), indicating no insulin-secreting tumour in that part of the pancreas. Following calcium injection into the artery supplying the tumour, insulin concentrations in the hepatic vein measured by RIA increased from 222 (range 102–893) pmol/l at baseline to 2899 (range 411–6691) pmol/l. Insulin gradients ranged from 2.3- to 24.2-fold (median 8.2), indicating the presence of an insulin-secreting tumour, i.e. all patients had an increase in insulin concentrations of more than twofold (Fig. 1).

Insulin concentrations in the hepatic vein measured by ELISA increased from 86 (range 14–582) pmol/l at baseline to 1650 (range 509–4468) pmol/l following calcium stimulation in the artery supplying the tumour. The increase in insulin concentrations measured by the specific ELISA was from 7.3- to 59.4-fold (median 16), i.e. significantly higher compared with the gradients obtained by RIA ($P = 0.006$, paired Student's t-test). Insulin gradients following calcium stimulation in the control artery were between 0.5- and 2.5-fold (median 1.1) when insulin was measured with the specific ELISA. In one patient, a 2.5-fold increase in insulin concentrations was found after calcium injection into a control artery.

Following calcium injection into the control artery proinsulin concentrations were 48 (range 5–323) pmol/l at baseline and 53 (range 6–303) pmol/l after the calcium injection. Following stimulation of the artery supplying the tumour, proinsulin concentrations increased from 45 (range 8–213) pmol/l at baseline to 93 (range 32–3524) pmol/l, i.e. a 1.3- to 16.5-fold increase (median 2.2).

Patients without an insulinoma ($n = 5$)

Data from each individual patient are shown in Table 1 and the gradients are shown in Fig. 1. One to three arteries per patient were stimulated. Insulin concentrations by RIA were 113 (range 34–461) pmol/l at baseline and 117 (range 65–434) pmol/l following calcium injection into the pancreatic artery; gradients ranged from 0.7- to 2.1-fold (median 1.3). Insulin concentrations in the hepatic vein measured by ELISA increased from 32 (range 2–217) pmol/l at baseline to 43 (range 9–236) pmol/l following calcium stimulation; the increase ranged between 0.6- and 4.7-fold (median 1.3).

Discussion

The present data show that the methodology of insulin determination is a critical issue in the evaluation of patients with fasting hyperinsulinaemic hypoglycaemic disorders. As previously described, insulin concentrations measured by specific insulin assays are markedly lower in insulinoma patients than the levels obtained by traditional RIAs (2, 11). During the prolonged fast, the assessment of hyperinsulinaemia in insulinoma patients may be obscured by the use of specific immunoassays without cross-reactivity for proinsulin when islet-cell tumours secrete predominantly proinsulin (12). Whether proinsulin secretion of insulinomas may be stimulated by local calcium stimulation during ASVS has never been assessed systematically. In the present study we demonstrate that the release of proinsulin by insulinoma cells following calcium stimulation varies considerably. However, proinsulin concentrations following calcium

Downloaded from Bioscientifica.com at 05/19/2019 03:47:35AM
via free access

stimulation in the artery supplying the insulinoma were significantly higher than baseline levels and increased in each individual patient. The large range of the proinsulin gradients among insulinoma patients might be explained by the well-recognised variability of insulinomas regarding the content of secretory granules for both proinsulin and insulin (13). Since the proinsulin gradients varied greatly and showed an overlap between gradients obtained after stimulation in the control artery and gradients obtained after stimulation in the artery supplying the insulinoma, measurement of proinsulin during ASVS does not improve the sensitivity and specificity in correctly localising an insulinoma.

Insulin determination by a specific insulin assay yielded significantly higher insulin gradients during ASVS than obtained by a traditional RIA, indicating that calcium stimulation preferentially released correctly processed insulin. Higher insulin gradients may facilitate the localisation of an insulin-secreting tumour. In our series, all insulinoma patients elicited a more than sevenfold increase in insulin concentrations after calcium injection into the artery supplying the tumour when insulin was measured by a specific immunoassay, in contrast to the more than twofold increase in insulin concentrations measured by RIA. The twofold increase indicating the localisation of an insulinoma by traditional RIA is in agreement with the literature and the recommendation of Doppman and co-workers (3). When this recommendation is applied to measurement with specific insulin assays, false positive ASVS may occur resulting in a lower specificity. In our study, a 2.5-fold increase in insulin concentration measured by the specific assay following calcium injection into a control artery was observed in one patient. This may be explained by stimulation of the insulinoma by recirculation or collateral vessels. However, in our experience with five patients undergoing the calcium stimulation test during celiac angiography of the pancreas without evidence of an insulin-secreting tumour, three patients elicited an increase in insulin concentrations measured by ELISA of more than twofold (from 2.8- to 4.7-fold). The maximum increase in insulin by RIA in these patients was 2.1-fold. Thus, the traditional criterion of a more than twofold increase in insulin concentrations to localise an insulinoma should not be applied when insulin is measured by specific insulin immunoassays. When insulin is measured by a more specific assay, a five- to sixfold increase in insulin concentrations is necessary to localise an insulinoma.

In conclusion, the release of proinsulin by insulinoma cells in response to calcium stimulation during ASVS varies greatly and determination of proinsulin during ASVS does not improve diagnostic accuracy. To prevent false positive ASVS, the traditional criterion of a two- to threefold increase in insulin concentrations to localise an insulinoma should not be applied when insulin is measured by specific immunoassays. In our experience, an increase in insulin concentrations of more than five- to sixfold when insulin is determined by specific immunoassays allows an insulinoma to be localised correctly.

Acknowledgements

We thank Heidi Seiler and Cornelia Zwimpfer for the determination of the β-cells peptides and Claudia Ghirlanda for technical assistance.

References

1 Service FJ. Hypoglycemic disorders. *New England Journal of Medicine* 1995 **332** 1144–1152.

2 Shimizu T, Sasakuma F, Ishikawa O, Matsumiya K, Hasegawa K & Sasaki A. Assessment of immunoassays for insulin in diagnostic tests for insulinoma. *Diabetes Research and Clinical Practice* 1994 **26** 149–154.

3 Doppman JL, Miller DL, Chang R, Shawker TH, Gorden P & Norton JA. Insulinomas: localization with selective intra-arterial injection of calcium. *Radiology* 1991 **178** 237–241.

4 Doppman JL, Chang R, Fraker DL, Norton JA, Alexander HR, Miller DL *et al.* Localization of insulinomas to regions of the pancreas by intra-arterial stimulation with calcium. *Annals of Internal Medicine* 1995 **123** 269–273.

5 O'Shea D, Rohrer-Theurs AW, Lynn JA, Jackson JE & Bloom SR. Localization of insulinomas by selective intra-arterial calcium injection. *Journal of Clinical Endocrinology and Metabolism* 1996 **81** 1623–1627.

6 Tsagarakis S, Kaskarelis J, Malagari C, Platis O, Trivizas P, Vrachliotis G *et al.* Regionalization of occult pancreatic insulinomas with the arterial stimulation venous sampling (ASVS) technique. *Clinical Endocrinology* 1997 **47** 753–757.

7 Kuzin NM, Egorov AV, Kondrashin SA, Lotov AN, Kuznetzov NS & Majorova JB. Preoperative and intraoperative topographic diagnosis of insulinomas. *World Journal of Surgery* 1998 **22** 593–597.

8 Pereira PL, Roche AJ, Maier GW, Huppert PE, Dammann F, Farnsworth CT *et al.* Insulinoma and islet cell hyperplasia: value of the calcium intra-arterial stimulation test when findings of other preoperative studies are negative. *Radiology* 1998 **206** 703–709.

9 Norton JA, Shawker TH, Doppman JL, Miller DL, Fraker DL, Cromack DT *et al.* Localization and surgical treatment of occult insulinomas. *Annals of Surgery* 1990 **212** 615–620.

10 Brandle M, Pfammatter T, Spinas GA, Lehmann R & Schmid C. Assessment of selective arterial calcium stimulation and hepatic venous sampling to localize insulin-secreting tumours. *Clinical Endocrinology* 2001 **55** 357–362.

11 Service FJ. Diagnostic approach to adults with hypoglycemic disorders. *Endocrinology and Metabolism Clinics of North America* 1999 **28** 519–532.

12 Chia CW & Saudek CD. The diagnosis of fasting hypoglycemia due to an islet-cell tumor obscured by a highly specific insulin assay. *Journal of Clinical Endocrinology and Metabolism* 2003 **88** 1464–1467.

13 Roth J, Komminoth P & Heitz PU. Topographic abnormalities of proinsulin to insulin conversion in functioning human insulinomas. Comparison of immunoelectron microscopic and clinical data. *American Journal of Pathology* 1995 **147** 489–502.

Received 15 September 2003
Accepted 1 April 2004

Downloaded from Bioscientifica.com at 05/19/2019 03:47:35AM
via free access
