# Supplementary material for: Diagnostic value of ASVS for insulinoma localization: A systematic review and meta-analysis
Source: PLoS One. 2019 Nov 19;14(11):e0224928. doi: 10.1371/journal.pone.0224928 (PMC6863549; doi:10.1371/journal.pone.0224928)
Supplement: S2 File — (ZIP) [file pone.0224928.s002.zip › included studies/Localization of insulinomas by selective intraarterial calcium.pdf]

0021-972X/96/$03.00/0
Journal of Clinical Endocrinology and Metabolism
Copyright © 1996 by The Endocrine Society
Vol. 81, No. 4
Printed in U.S.A.

Localization of Insulinomas by Selective Intraarterial Calcium Injection

D. O'SHEA, A. W. ROHRER-THEUS, J. A. LYNN, J. E. JACKSON, AND S. R. BLOOM

Departments of Medicine (D.O., A.W.R.-T., S.R.B.), Surgery (J.A.L.), and Radiology (J.E.J.), Royal Postgraduate Medical School, Hammersmith Hospital London, United Kingdom W12 OHS

ABSTRACT

This study examines the role of selective intraarterial calcium injection and hepatic venous sampling in the localization of insulinomas. Seven patients were studied. In all cases, ultrasound and computerized tomography scans were either negative or equivocal. Calcium gluconate was injected directly into the arteries supplying the pancreas after standard selective angiography. Insulin levels were measured in samples taken from the right hepatic vein before and 30, 60, 90, 120, and 180 s after each injection. Two doses were used, 0.025 milliequivalents Ca/kg (1 mg/kg) for the first two subjects and 0.00625 milliequivalents Ca/kg (0.25 mg/kg) for the remaining five subjects. Serum insulin levels rose at least 2-fold, the proposed diagnostic rise, from basal in six subjects; one test was negative.

Of the six positive studies, a diagnostic rise was seen only in one artery in five cases. One patient did not undergo surgery. In the remaining five patients, surgery confirmed the position and histology of the tumor. The one patient with a diagnostic rise in more than one artery, however, had residual disease after surgery. The seventh subject referred specifically for localization had a negative calcium stimulation study and a subsequent diagnosis of intermittent sulfonylurea abuse was made after a positive screen.

The present study shows that preoperatively, selective intraarterial calcium injection with hepatic venous sampling is a powerful technique for the localization of insulinomas. Smaller doses of calcium than previously reported can be used and may reduce the risk of hypoglycemia during the procedure. (*J Clin Endocrinol Metab* **81:** 1623–1627, 1996)

INSULINOMAS ARE rare endocrine tumors with an estimated incidence of 1 per million. The optimal therapy for islet cell tumors of the pancreas is curative surgical resection. Many researchers agree that this is more likely to be successful if the tumors have been localized preoperatively (1).

Computerized tomography (CT) and transabdominal ultrasound (US) will detect up to 60% (2, 3) of biochemically proven insulinomas. At present, relatively little has been reported about magnetic resonance imaging (MRI) in the diagnosis of islet cell tumors, and its exact role is uncertain (4). The sensitivity of angiography has been reported to be up to 64% (5, 6) for the diagnosis of insulinomas, whereas portal venous sampling localizes 81% (7) to 100% (8) of these tumors. The latter is, however, invasive and expensive and has a significant associated morbidity.

Over the last decade, a higher prevalence (>50%) of occult insulinomas has been reported (9, 10) (*i.e.* those with negative or equivocal imaging studies, including arteriography, US, CT, and MRI).

Imamura *et al.* (11) used secretin for selective intraarterial stimulation and hepatic venous sampling to localize gastrinomas in 1987. Doppman *et al.* in 1991 (12) reported four cases of successful localization of insulinomas using 0.01–0.025 milliequivalents/kg calcium gluconate as the secretagogue. In 1993, authors from the same institution reported successful localization of an additional six cases (13). We have modified this procedure using the selective intraarterial injection of a lower dose of calcium and right hepatic venous sampling only. We have now performed this in seven consecutive cases.

Subjects and Methods

Patients

Seven consecutive patients were studied, four women and three men. Their average age was 47.8 yr, with a range of 26–73 yr. All patients gave written informed consent. In six patients, the biochemical criteria for the diagnosis of an insulinoma were met during a prolonged fast. That is, they had symptomatic hypoglycemia (blood glucose, <40 mg/dL) in the face of inappropriately normal or elevated insulin and C peptide levels. Sulfonylurea screens were performed and were negative in six subjects. In the seventh subject, the sulfonylurea screen was positive for glibenclamide on reassessment during a prolonged fast after a negative intraarterial calcium stimulation test. A sulfonylurea screen at the referring center had been negative, but had only assessed tolbutamide and chlorpropamide. In all cases, CT and US had not identified a tumor, and the patients went on to have angiography followed by selective intraarterial calcium injection. Radiolabeled somatostatin analog scanning and intraoperative ultrasonography were not used in the patients described.

Procedure

A catheter was placed in the right hepatic vein close to its junction with the inferior vena cava via a femoral venous puncture before standard selective visceral arteriography. The right hepatic vein only was chosen, because in the original cases described, despite catheterization of both right and left hepatic veins, no isolated left sided gradients were seen. It was suggested that catheterization of the left hepatic vein, which is technically more difficult, may be unnecessary. In addition to reducing the technical difficulty of the procedure, the amount of sampling required is reduced.

After full standard angiography, each artery (usually proximal gastroduodenal, proximal splenic, inferior pancreaticoduodenal artery, proper hepatic, and superior mesenteric) was recatheterized in turn. If a vascular lesion consistent with an insulinoma had been seen on angiography, the vessel supplying this region was studied last. This was to reduce the possibility of hypoglycemia occurring during the proce-

Received April 19, 1995. Revision received October 5, 1995. Accepted November 10, 1995.

Address all correspondence and requests for reprints to: Dr. S. R. Bloom, Department of Medicine, Royal Postgraduate Medical School, London W12 OHS, United Kingdom.

Downloaded from https://academic.oup.com/jcem/article-abstract/81/4/1623/2875432
by University of Colorado systems user
on 16 December 2017

dure, as occurred in one patient (patient 3). The hepatic artery supplies the liver, the splenic artery supplies primarily the body and tail of the pancreas, and the gastroduodenal and superior mesenteric arteries (via the pancreaticoduodenal arcade) supply the pancreatic head and uncinate process. We defined a more than 2-fold rise in insulin levels within 30–120 s after the injection of calcium as indicating the presence of a tumor in that portion of the pancreas supplied by the artery studied.

After two basal hepatic venous samples (standard in our unit), calcium gluconate, 10% diluted to 5 mL normal saline, was injected as a bolus into the artery at time zero. At least 5 min were left between each calcium injection. The dose of calcium used was 1 mg/kg·injection for the first three studies. This was reduced to 0.25 mg/kg for the subsequent studies because of an episode of symptomatic hypoglycemia in our third subject after calcium injection. She subsequently underwent a repeat study using the lower dose of calcium without complication.

Blood was sampled from the right hepatic vein before and 30, 60, 90, 120, and 180 s after each injection. The samples for insulin assay only were separated within 30 min and assayed over the following 3 days.

Results

Subject 1

A 28-yr-old woman had episodes of loss of awareness and bizarre behavior over 4 yr. All attacks were abolished by eating. The diagnosis of an insulinoma was made after the finding of an inappropriately elevated serum insulin level during a hypoglycemic episode; a sulfonylurea screen was negative. A CT scan of the abdomen was normal, and angiography indicated a tumor blush in the head of the pancreas. At surgery, no tumor was found despite extensive mobilization of the pancreas. Intraoperative ultrasound was not performed. The patient continued to be symptomatic, with frequent hypoglycemia. An angiogram was repeated, which again demonstrated a tumor blush within the pancreatic head. Selective intraarterial calcium injection (60 mg) caused a 10-fold increase in insulin release when injected into the inferior pancreatico-duodenal artery (Fig. 1). A pancreatico-duodenectomy was performed, and subsequent histology revealed a 3-mm islet cell tumor in the head of the pancreas. The patient has had no more symptoms of hypoglycemia during a follow-up period of 24 months.

Subject 2

A 73-yr-old man with chronic renal impairment on chronic ambulatory peritoneal dialysis presented with episodes of loss of speech and weakness over 18 months. Up to this time he had been extremely well and active despite his renal failure. A decision was taken to manage him surgically if possible. Biochemical investigations revealed hypoglycemia, elevated insulin levels, and a negative sulfonylurea screen. A

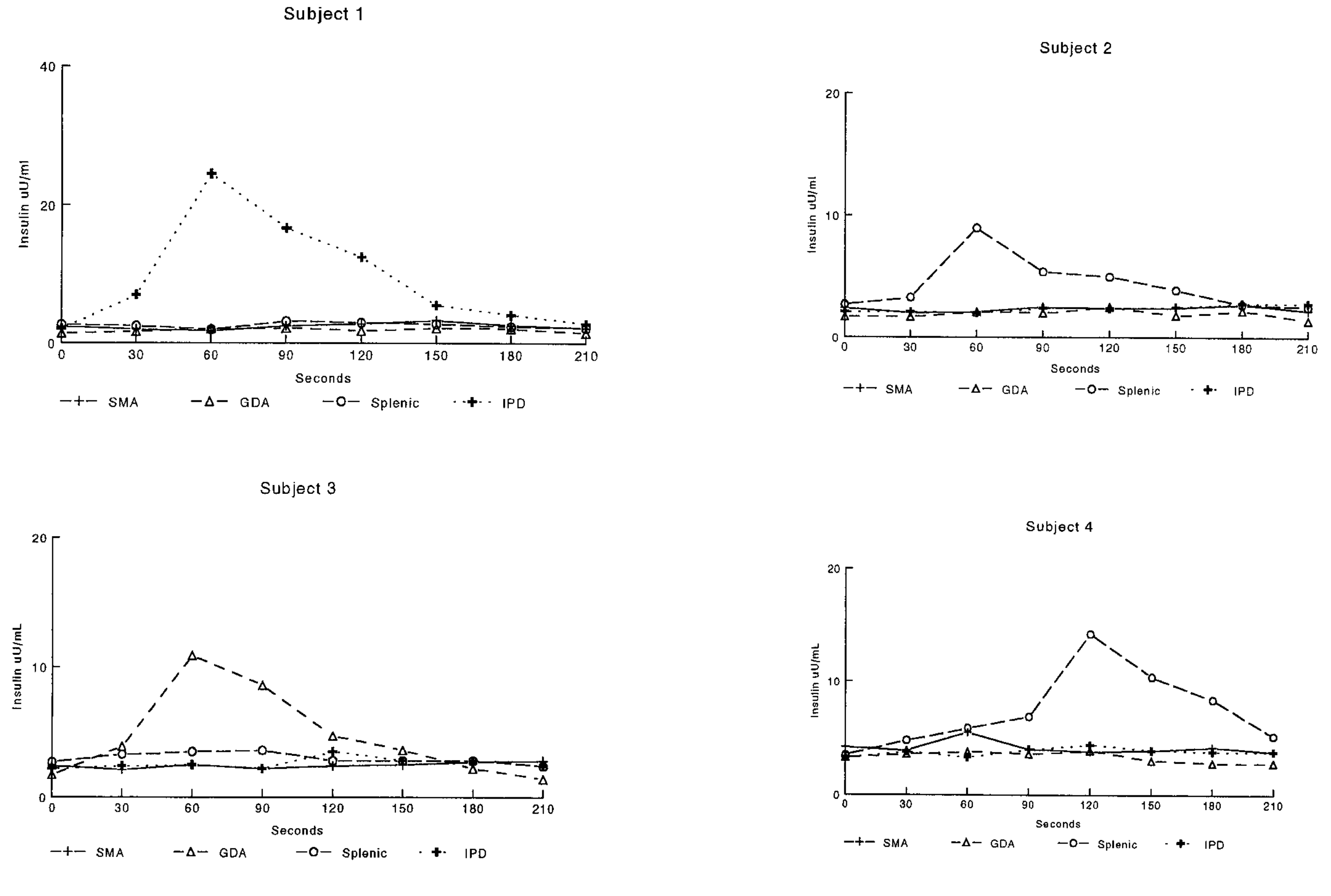

SMA=Superior mesenteric artery. GDA=Gastroduodenal artery. IPD=Inferior pancreaticoduodenal artery.

FIG. 1. Insulin release determined after selective intraarterial calcium injection in subjects 1–4.

Downloaded from https://academic.oup.com/jcem/article-abstract/81/4/1623/2875432
by University of Colorado systems user
on 16 December 2017

CT scan of the abdomen showed a possible lesion in the tail of the pancreas. Angiography confirmed a poorly defined vascular blush within the pancreatic tail and raised the possibility of hepatic metastases. Selective intraarterial calcium injection (74 mg) showed a 3-fold rise of insulin in the splenic artery only (Fig. 1). In view of a subsequent unexpected deterioration in his general condition, he was treated medically with diazoxide and had good symptom control until his death 12 months later. No post mortem examination was performed.

Subject 3

A 62-yr-old woman had a biochemically proven insulinoma that had not been localized on US, CT, or initial angiogram. A sulfonylurea screen was negative. She was treated with diazoxide and had good symptom control for 4 yr. After relapse and further negative CT and US scans, angiography with selective intraarterial calcium injection (65 mg) was performed. A 15-mm diameter vascular tumor blush was demonstrated within the pancreatic head, but the stimulation study was complicated by hypoglycemia after the first bolus of calcium gluconate into the gastroduodenal artery. Five minutes after the injection, the patient became very agitated, complained of severe headache, and began to sweat. Her blood glucose fell to 30 mg/dL. She responded to iv glucose within 3 min, but remained agitated, and the investigation was abandoned. The insulin samples were not measured. One month later, a second selective intraarterial calcium stimulation using 25% of the standard dose of calcium (16 mg) demonstrated a 6-fold rise in insulin after injection into the gastroduodenal artery consistent with a tumor in the pancreatic head (Fig. 1). At surgery, an islet cell tumor of 15 mm in diameter was enucleated from the head of the pancreas. The patient currently has no evidence of hypoglycemia during a follow-up of 20 months.

Subject 4

A 45-yr-old man had two episodes of proven hypoglycemia with elevated insulin and C peptide levels and a negative sulfonylurea screen. CT of the abdomen demonstrated no pancreatic lesion, but at visceral angiography, a 15-mm vascular blush was seen in the pancreatic tail. Selective intraarterial calcium injection (20 mg) into the splenic artery demonstrated a 4-fold rise in insulin concentration, indicating the presence of the insulinoma in this area (Fig. 1). At subsequent surgery, a solitary insulinoma of 15 mm in diameter was removed from the tail of the pancreas. The patient remains well at 12 months follow-up.

Subject 5

A 40-yr-old man presented with symptoms of weakness and double vision on minimal exertion. He had proven hypoglycemia, elevated insulin levels, and a negative sulfonylurea screen. CT and US scans failed to localize a tumor. Angiography was negative, and selective intraarterial calcium injection (18 mg) revealed a 7-fold increase in insulin release in the distribution of the splenic artery (Fig. 2). At surgery, an isolated 8-mm diameter insulinoma was enucleated from the tail of the pancreas. The patient has had no further episodes of hypoglycemia at 8 months follow-up.

Subject 6

A 50-yr-old woman presented with a 9-yr history of symptoms suggestive of an insulinoma and had a proven episode of hypoglycemia with raised insulin and C peptide levels. A sulfonylurea screen was negative. Two CT scans suggested bulkiness in the tail of the pancreas. Visceral angiography demonstrated a classical 10-mm blush in the tail of the pancreas (splenic artery) and a second possible lesion in the pancreatic head (common hepatic artery). The arterial stimulation study (14 mg calcium) revealed an increased distribution in both of these arteries (Fig. 2). This indicated the presence of a tumor in the tail and either the head of the pancreas or hepatic metastases. At surgery, an islet cell tumor 10 mm in diameter was removed from the tail of the pancreas. Despite mobilization and palpation, no other lesions were found. Histology indicated this to be an invasive tumor. The patient remained asymptomatic for 2 months after surgery, but then developed recurrent hypoglycemia. A repeat arteriogram again demonstrated a vascular blush within the pancreatic head, and a calcium stimulation study produced a 4-fold increase in insulin release in the distribution of the common hepatic artery (Fig. 2). These findings suggested the presence of a second tumor in the pancreatic head or hepatic metastases. There was no evidence of hepatic metastases on CT scan or angiography. In view of her recent pancreatic resection, she is currently being treated medically. Her symptoms are well controlled with diazoxide at 7 months follow-up.

Subject 7

A 26-yr-old woman presented with an 8-month history of episodic lightheadedness. Her previous medical history included noninsulin-dependent diabetes that had been managed with glibenclamide for 1 yr and subsequently by diet alone. She had episodes of proven hypoglycemia, with raised insulin and C peptide levels. A sulfonylurea screen at her referring center was negative. Other investigations performed at the referring hospital (US scan, CT scan, MRI, octreotide scan, and visceral angiography) were also negative. She was then referred to this unit for repeat angiography with selective intraarterial calcium injection. The investigation (16 mg calcium) was negative (Fig. 2). During a further episode of hypoglycemia as an in-patient she had a strongly positive screen for glibenclamide, indicating factitious hypoglycemia.

Discussion

Surgical resection of insulin-secreting tumors of the pancreas is likely to be curative, but failure to resect a tumor because of an inability to localize it preoperatively or at surgery occurs in up to 10% of patients with these lesions (14). In addition to improving surgical outcome, accurate preoperative localization of the insulinoma greatly improves patient confidence and allows informed discussion about the nature and extent of the likely surgery.

The choice of an agent to stimulate insulin release from a

Downloaded from https://academic.oup.com/jcem/article-abstract/81/4/1623/2875432
by University of Colorado systems user
on 16 December 2017

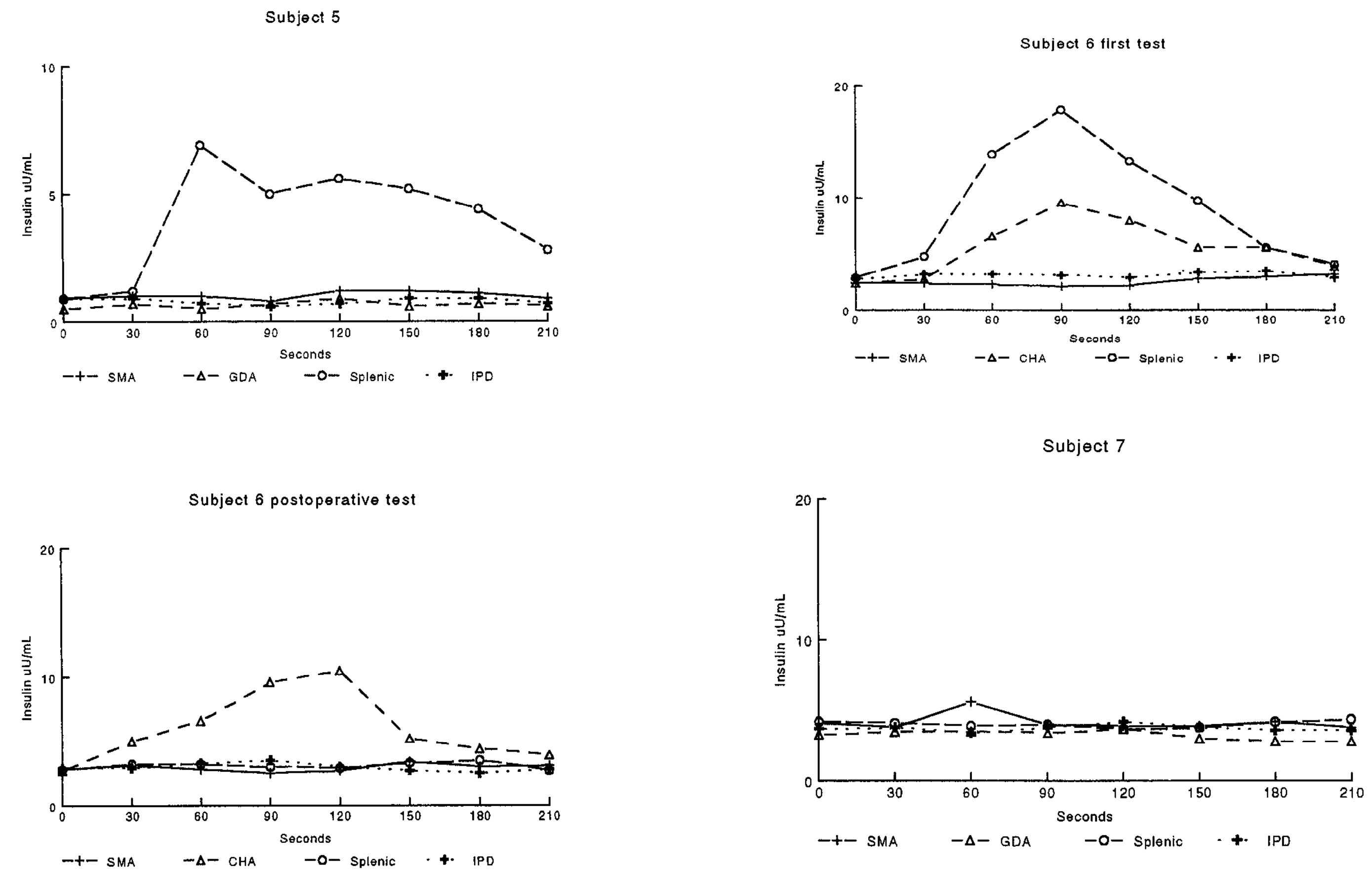

SMA=Superior Mesenteric artery. GDA=Gastroduodenal artery. IPD=Inferiorpancreaticoduodenal artery. CHA = Common hepatic artery

FIG. 2. Insulin release determined after selective intraarterial calcium injections in subjects 5–7.

β-cell tumor of the pancreatic islet is controversial. Some researchers have proposed glucagon (15), tolbutamide (16), or leucine (16) as secretagogues, but these agents are not universally accepted. Calcium was reported to cause an elevation of peripheral insulin levels within 1 min after rapid iv injection (0.4 mg calcium/kg) in 11 patients with insulin-secreting tumors of the pancreas. Sixteen healthy control subjects only showed a minimal response (17).

In 1991, four cases of successful localization of insulinomas using intraarterial calcium gluconate (1–2.5 mg calcium/kg) as the secretagogue with hepatic venous sampling were described (12). This technique is reported to facilitate the operative localization of insulin-producing islet cell tumors (13).

We have now carried out a modification of this procedure in seven consecutive cases with suspected insulin-producing tumors. We collected venous blood from the right hepatic vein only, as opposed to both the left and right hepatic veins, and the sensitivity of this procedure appears as good as that in studies in which samples from both hepatic veins were collected. In keeping with other researchers, we defined a 2-fold or more increase in insulin from baseline as being indicative of a tumor in the vascular territory of the artery studied. This rise may occur between 30–120 s after injection, rather than up to 60 s thereafter as previously proposed (based on our findings in subject 4).

Finally, in five of our seven patients, we obtained diagnostic studies using a calcium dose of 25% of that previously reported. In five of our six patients with tumors, the insulinomas were accurately localized using this technique, and this was confirmed by surgery in four subjects. The exception (subject 6) on initial investigation demonstrated a 2-fold rise in insulin release in the distribution of two arteries (splenic and common hepatic artery). Surgery has failed to resect all of her disease, and a postoperative calcium stimulation test suggests the presence of either a second tumor in the pancreatic head or perhaps hepatic metastases, although none was seen on selective hepatic arteriography or CT scan. This is the only patient in whom we found a diagnostic rise in more than one artery. At repeat angiography and calcium stimulation, a selective injection of the proper hepatic artery and gastroduodenal artery would have been helpful in differentiating the possible lesion in the head of the pancreas from hepatic metastases. We recommend caution in the management of cases in which a rise occurs in more than one artery and suggest that this finding should alert one to the likelihood of multiple tumors or metastatic disease. Detailed

Downloaded from https://academic.oup.com/jcem/article-abstract/81/4/1623/2875432
by University of Colorado systems user
on 16 December 2017

study of the initial stimulation test and the aims of the second study should be defined before undertaking a repeat investigation.

The negative test in subject 7 emphasizes the absolute need to confirm the diagnosis before attempting localization. It also highlights the caution with which an apparently negative sulfonylurea screen should be treated, as many screens check for a limited number of the sulfonylureas.

There are a number of techniques that have been reported to be highly sensitive for the intraoperative detection of islet cell tumors. Intraoperative US combined with palpation is reported to localize up to 100% of tumors (17). The more recently described technique in which a hand-held γ-camera is used after the injection of radiolabeled octreotide for intraoperative localization of neuroendocrine tumors (18) may also facilitate the management of insulinomas; this procedure requires further evaluation.

Preoperatively, angiography and portal venous sampling have been reported to be the most sensitive investigations for the detection of these tumors, but portal venous sampling is associated with significant morbidity. Angiography, however, requires meticulous technique, superselective catheterization, and an experienced operator. In expert hands an insulinoma will be detected in over 90% of patients (19). It has been suggested, however, that occult tumors are becoming more common, although this is not the experience at our institution (20, 21), where all arteriograms are performed by the same individual (J.E.J.). Despite the high sensitivity of angiography for the detection of insulinomas in this series, the arterial calcium stimulation test with hepatic venous sampling proved to be extremely useful in several of our patients. For example, as in subject 1, in whom a definite vascular tumor was seen but no tumor was palpable in this region at surgery, a positive calcium stimulation test allowed the surgeon to be more confident about proceeding with a Whipple's pancreatic resection. Furthermore, in subject 5, in whom no tumor was seen at angiography, a positive calcium stimulation test provided strong evidence that the tumor was located within the distal pancreas, as was indeed found to be the case at subsequent surgery. This allowed the surgeon to inform the patient that a distal pancreatectomy would probably be required rather than a more difficult pancreatic head resection. Finally, the arteriographic findings are occasionally subtle, and in such cases it may not be possible to confidently localize the tumor. The presence of a positive calcium stimulation test in the same arterial territory as a vascular abnormality helps to confirm this as being associated with a tumor.

The calcium stimulation test with hepatic venous sampling may also be useful in the assessment of patients with multiple endocrine neoplasia type 1 who have a biochemically proven insulinoma. Such patients will frequently have multiple pancreatic tumors, some of which may be nonfunctioning. These tumors may have similar angiographic appearances, whereas the calcium stimulation study will allow more accurate localization of the functioning neoplasm(s). This indication may become more important because of the ability to detect affected individuals by genetic screening.

In subject 4, the diagnostic rise was seen at 90 s, and the position of the tumor was confirmed at surgery. We recommend that the time frame for a diagnostic rise should be up to 120 s after injection, and not just the 30 and 60 s previously proposed.

In subject 3, symptomatic hypoglycemia followed injection of the calcium bolus. Although hypoglycemia is an obvious potential complication of this test, it has not previously been reported. In this subject, when the test was repeated with 25% of the original dose, she did not develop hypoglycemia, and the tumor was successfully localized. We used this dose of 0.00625 milliequivalents/kg calcium gluconate (0.25 mg/kg calcium) successfully in our subsequent tests. Thus, smaller doses of calcium than previously reported may be used successfully to localize the tumors, and it is possible that this may reduce the incidence of hypoglycemia during the procedure.

The present study shows that preoperatively, selective intraarterial injection of calcium gluconate with hepatic venous sampling is a powerful technique for the localization of insulinomas.

References

1. **Debas HT, Mulvihill SJ.** 1994 Neuroendocrine gut neoplasms. Important lessons from uncommon tumors. Arch Surg. 129:965–971.
2. **Proye C, Boissel P.** 1988 Preoperative imaging *versus* intra-operative localization of tumors in adult surgical patients with hyperinsulinaemia: a multicenter study of 338 patients. World J Surg. 12:685–690.
3. **Grant CS, van Heerden J, Charboneau JW, James EM, Reeding CC.** 1988 Insulinoma: the value of intraoperative ultrasonography. Arch Surg. 123:843–848.
4. **King CMP, Reznek RH, Dacie JE, Wass JAH.** 1994 Review: imaging islet cell tumours. Clin Radiol. 49:295–303.
5. **Roche A, Raisonnier A, Gillon-Savouret MC.** 1982 Pancreatic venous sampling and arteriography in localizing insulinomas and gastrinomas: procedure and results in 55 cases. Radiology. 145:621–627.
6. **Bottger TC, Weber W, Beyer J, Junginger T.** 1990 Value of tumor localization in patients with insulinoma. World J Surg. 14:107–112.
7. **Vinik AI, Delbridge L, Moattari R, Cho K, Thompson N.** 1991 Transhepatic portal vein catheterization for localization of insulinomas: a ten year experience. Surgery. 109:1–11.
8. **Fraker DL, Norton JA.** 1988 Localization and resection of islet cell tumors of the pancreas. JAMA. 259:3601–3605.
9. **Doppman JL, Shawker TH, Miller DL.** 1989 Localization of islet cell tumors. Gastrointest Clin North Am. 18:793–804.
10. **Norton JA, Shawker TH, Doppman JL, et al.** 1990 Localization and surgical treatment of occult Insulinomas. Ann Surg. 212:615–620.
11. **Imamura M, Takahashi K, Adachi H, et al.** 1987 Usefulness of selective arterial secretin test for localization of gastrinoma in the Zollinger-Ellison syndrome. Ann Surg. 205:230–239.
12. **Doppman JL, Miller DL, Chang R, Shawker TH, Gordon P, Norton JA.** 1991 Insulinomas: localization with selective intraarterial injection of calcium. Radiology. 178:237–241.
13. **Doppman JL, Miller DL, Chang R, Gordon P, Eastman RC, Norton JA.** 1993 Intraarterial calcium stimulation test for detection of insulinomas. World J Surg. 17:439–443.
14. **Fedorak IJ, Ko TC, Gordon D, Flisak M, Prinz RA.** 1993 Localization of islet cell tumors of the pancreas: a review of current techniques. Surgery. 113:242–249.
15. **Kumar D, Mehtalia SD, Miller LV.** 1974 Diagnostic use of glucagon- induced insulin response. Ann Intern Med. 80:697–701.
16. **Floyd Jr JC, Fajans SS, Knopf RF, Conn JW.** 1964 Plasma insulin in organic hyperinsulinism: comparative effects of tolbutamide, leucine and glucose. J Clin Endocrinol Metab. 24:747–760.
17. **Brunt LM, Veldhuis JD, Dilley WG, et al.** 1986 Stimulation of insulin secretion by a rapid intravenous calcium infusion in patients with β-cell neoplasms of the pancreas. J Clin Endocrinol Metab. 62:210–216.
18. **Galiber AK, Reading CC, Charboneau JW, et al.** 1988 Localization of pancreatic insulinoma: comparison of pre- and intraoperative US with CT and angiography. Radiology. 166:405–408.
19. **Schirmer WJ, O'Dorisio TM, Schirmer TP, Mojzisik CM, Hinkle GH, Martin EW.** 1993 Intraoperative localization of neuroendocrine tumors with ^{125}I-TYR(3)-octreotide and a hand-held gamma-detecting probe. Surgery. 114:745–752.
20. **Hammond PJ, Jackson JE, Bloom SR.** 1994 Localization of pancreatic endocrine tumours. Clin Endocrinol (Oxf). 40:3–14.
21. **Geoghegan JG, Jackson JE, Lewis MPN, et al.** 1994 Localization and surgical management of insulinoma. Br J Surg. 81:1025–1028.

Downloaded from https://academic.oup.com/jcem/article-abstract/81/4/1623/2875432
by University of Colorado systems user
on 16 December 2017
