# Supplementary material for: Diagnostic value of ASVS for insulinoma localization: A systematic review and meta-analysis
Source: PLoS One. 2019 Nov 19;14(11):e0224928. doi: 10.1371/journal.pone.0224928 (PMC6863549; doi:10.1371/journal.pone.0224928)
Supplement: S2 File — (ZIP) [file pone.0224928.s002.zip › included studies/localization of insulinomas of region NIH experience.pdf]

Localization of Insulinomas to Regions of the Pancreas by Intraarterial Calcium Stimulation: The NIH Experience

Jean-Marc Guettier, Anthony Kam, Richard Chang, Monica C. Skarulis, Craig Cochran, H. Richard Alexander, Steven K. Libutti, James F. Pingpank, and Phillip Gorden†

National Institute of Diabetes and Digestive and Kidney Disease (J.-M.G., M.C.S., C.C., P.G.), National Institutes of Health, Bethesda, Maryland, 20892; Radiology Department (A.K., R.C.), Warren Grant Magnusson Clinical Center, National Institutes of Health, Bethesda, Maryland 20892; Surgical Metabolism Section (H.R.A., S.K.L., J.F.P.), Surgery Branch, National Cancer Institute, National Institutes of Health, Bethesda, Maryland, 20892; Department of Radiology (A.K.), Johns Hopkins Bayview Medical Center, Baltimore, Maryland 21224; and Department of Surgery (H.R.A.), University of Maryland Medical Center, Baltimore, Maryland 21201

Downloaded from https://academic.oup.com/jcem/article-abstract/94/4/1074/2596210 by MIT Libraries user on 17 March 2019

Context: Selective intraarterial calcium injection of the major pancreatic arteries with hepatic venous sampling [calcium arterial stimulation (CaStim)] has been used as a localizing tool for insulinomas at the National Institutes of Health (NIH) since 1989. The accuracy of this technique for localizing insulinomas was reported for all cases until 1996.

Objectives: The aim of the study was to assess the accuracy and track record of the CaStim over time and in the context of evolving technology and to review issues related to result interpretation and procedure complications. CaStim was the only invasive preoperative localization modality used at our center. Endoscopic ultrasound (US) was not studied.

Design and Setting: We conducted a retrospective case review at a referral center.

Patients: Twenty-nine women and 16 men (mean age, 47 yr; range, 13–78) were diagnosed with an insulinoma from 1996–2008.

Intervention: A supervised fast was conducted to confirm the diagnosis of insulinoma. US, computed tomography (CT), magnetic resonance imaging (MRI), and CaStim were used as preoperative localization studies. Localization predicted by each preoperative test was compared to surgical localization for accuracy.

Main Outcome: We measured the accuracy of US, CT, MRI, and CaStim for localization of insulinomas preoperatively.

Results: All 45 patients had surgically proven insulinomas. Thirty-eight of 45 (84%) localized to the correct anatomical region by CaStim. In five of 45 (11%) patients, the CaStim was falsely negative. Two of 45 (4%) had false-positive localizations.

Conclusion: The CaStim has remained vastly superior to abdominal US, CT, or MRI over time as a preoperative localizing tool for insulinomas. The utility of the CaStim for this purpose and in this setting is thus validated. (*J Clin Endocrinol Metab* **94: 1074–1080, 2009)**

ISSN Print 0021-972X ISSN Online 1945-7197
Printed in U.S.A.
Copyright © 2009 by The Endocrine Society
doi: 10.1210/jc.2008-1986 Received September 9, 2008. Accepted January 8, 2009.
First Published Online February 3, 2009

† This manuscript is dedicated to the memory of Dr. John. L. Doppman, a brilliant pioneer of interventional radiology, specifically as related to endocrine disease.

Abbreviations: CaStim, Calcium arterial stimulation; CT, computed tomography; EUS, endoscopic US; GDA, gastroduodenal artery; MEN-1, multiple endocrine neoplasia type 1; MRI, magnetic resonance imaging; ProxSplenic, proximal splenic; SMA, superior mesenteric artery; US, ultrasound.

Insulinomas are the most common islet cell tumors (1, 2). The diagnosis rests on establishing evidence of symptomatic hypoglycemia accompanied by inappropriate insulin and or proinsulin levels after a period of prolonged fasting (3, 4). When these tumors come to clinical attention, they are usually small, solitary, and intrapancreatic. Surgical resection of the tumor is usually curative (5). The clinical challenge in insulinomas lies in their localization. Blind distal pancreatectomies are not recommended because of their low likelihood of cure and high complication rates (6). Preoperative localization, in the work-up of insulinoma, increases the chance for successful surgical resection, minimizes operating room time, and obviates the need for repeat operations associated with high morbidity (7–10). Despite advances in imaging techniques and the development of new localization procedures, tumors less than 2.0 cm in size remain difficult to localize by conventional means. Selective intraarterial calcium injection of the major pancreatic arteries [calcium arterial stimulation (CaStim)] with hepatic venous sampling for insulin was developed by Doppman *et al.* (11–14) in 1989 as a way to localize discrete insulin-secreting islet cell tumors to regions of the pancreas. This technique is based on the premise that tumor cells differ from normal β-cells in their insulin response to an intraarterial calcium injection (15–18). In a case series of 25 surgically proven sporadic insulinomas (13), CaStim had the highest accuracy (88%) for localizing insulinomas to regions of the pancreas compared with magnetic resonance imaging (MRI) (43%), arteriography (36%), computed tomography (CT) (17%), and ultrasonography (9%). Since our last published report (14), an additional 45 patients have undergone the procedure at the National Institutes of Health (NIH). This report evaluates the usefulness of the CaStim for localizing discrete insulin-producing tumors over time and in the context of evolving imaging and surgical techniques. In addition, caveats related to result interpretation are discussed, and a case illustrating a rare complication of the procedure is presented.

Patients and Methods

Patients referred to our center with a diagnosis of fasting hypoglycemia and negative outside localization studies were enrolled in the NIH hypoglycemic disorders protocol. Symptomatic hypoglycemia (≤45 mg/ml) together with elevated plasma insulin, proinsulin, and C-peptide levels were confirmed by means of a supervised fast. Other causes of hypoglycemia were excluded by usual means. Noninvasive localization studies included abdominal CT, MRI, and transabdominal ultrasound (US). CaStim was recommended in patients lacking localization on at least two noninvasive imaging studies. Endoscopic US (EUS) is not routinely used at our center. CT was performed using multislice scanners [4-slice Light Speed QX/i (General Electric Healthcare Technologies, Waukesha, WI), 8-slice Light Speed Ultra (General Electric Healthcare Technologies), or 16-slice Mx8000 IDT 16 (Philips Medical Systems, Best, The Netherlands)] in three vascular phases. After precontrast images reconstructed to 5-mm thickness were obtained, arterial phase images reconstructed to 2.5- or 2.0-mm thickness and venous phase images reconstructed to 5-mm thickness were obtained during the administration of 130 ml of iopamidol (Isovue 300; Bracco Diagnostics Inc., Princeton, NJ) at 4 ml/sec. MRI was performed with 1.5-Tesla (T) (Signa 1.5 T, General Electric Healthcare Technologies) or 3.0-T (Intera 3.0 T, Philips Medical Systems) scanners using the following sequences: axial fat-suppressed T2-weighted fast spin echo (6-mm thick), three-dimensional fat-suppressed dynamic gadolinium-enhanced T1-weighted spoiled gradient echo [precontrast, arterial, venous, and delayed venous phases; 0.2 ml/kg of gadopentetate dimeglumine (Magnevist; Berlex Laboratories, Wayne, NJ) given at 2 ml/sec] reconstructed axially to a 5-mm thickness, and axial and coronal delayed fat-suppressed T1-weighted spoiled gradient echo (8-mm thick). Transabdominal US was performed with a HDI 5000 (Philips Medical Systems), iU22 (Philips Medical Systems), or Acuson Sequoia 512 (Siemens Medical Solutions, Malvern, PA). The curved array C5–2 transducer was used with the HDI 5000 and iU22; the 4V1 transducer was used with the Acuson Sequoia.

The technique of visceral arteriography and calcium arterial stimulation with hepatic venous sampling for insulinoma localization has changed slightly since the description of Doppman *et al.* (13) in 1995. Visceral arteriography was performed with selective injections of nonionic contrast (Isovue 300) into the superior mesenteric, proximal, and midsplenic, gastroduodenal, and proper hepatic arteries. The midsplenic artery was defined as just distal to the origin of the pancreatic magna artery. Because the major pancreatic arteries were typically perfused in these arteriograms, selective arteriography of the dorsal pancreatic and pancreatic magna arteries was rarely necessary. If dorsal pancreatic and pancreatic magna arteriograms were done, calcium was not infused into these arteries. After each selective arteriogram, 10% calcium gluconate (American Pharmaceutical Partners, Inc., Schaumburg, IL) diluted to a volume of 5 ml with normal saline was bolused into the selected artery at a dose of 0.0125 mmol Ca^{2+}/kg (0.025 mEq/kg) body weight. In obese patients, the dose was adjusted to 0.005 mmol Ca^{2+}/kg. Five-milliliter blood samples from the right and left hepatic veins were obtained before and 20, 40, and 60 sec after calcium injection. The samples were kept on ice until they could be centrifuged, and the resulting plasma was stored at −20 C. Insulin levels were measured by RIA or immunochemiluminometric assay. Because this procedure is performed after an overnight fast, two peripheral antecubital infusions of 5% dextrose are running for the length of the procedure.

Interpretation of the CaStim response

A 2-fold or greater step-up in right hepatic vein insulin concentration from baseline at times 20, 40, and/or 60 sec after arterial calcium injection constitutes a positive response (13). In the absence of anatomical variants, a positive response when the gastroduodenal artery (GDA) or superior mesenteric artery (SMA) is injected predicts a head/neck lesion. A positive response after proximal splenic (ProxSplenic) artery or midsplenic (MidSplenic) artery injection predicts regionalization to the body and tail region. A positive response after a proper hepatic artery injection represents liver metastases. Localization based on CaStim was compared with surgical tumor location. Analysis of localization sensitivities for response thresholds other than 2-fold is provided in Supplemental Fig. 1 (published as supplemental data on The Endocrine Society's Journals Online web site at http://jcem.endojournals.org).

Interpretation of the results is based on the minimum criterion for a positive response, defined as a 2-fold step-up in baseline hepatic insulin after arterial calcium injection. However, a 2-fold step-up may be seen in more than one artery. When a positive response was elicited at more than one injection site, the dominant site was used to predict tumor localization. Pancreatic arterial anatomy predicts that some of these multiple responses represent overlap in the tumors arterial supply (*e.g.* GDA and SMA for head/neck and ProxSplenic and MidSplenic for body/tail lesions). False-positive, false-negative, and inconsistent results were reviewed by interventional radiology to gain better understanding of the caveats associated with this technique.

Results

Demographic, biochemical, and diagnostic data

Forty-five patients (29 women, 16 men) with a mean age of 47 yr (range, 13–78) were referred for fasting hypoglycemia, a pos-

Downloaded from https://academic.oup.com/jcem/article-abstract/94/4/1074/2596210 by MIT Libraries user on 17 March 2019

itive supervised fast, and negative localization studies. A supervised fast was repeated to confirm the diagnosis of insulinoma. The mean fast time was 17.0 h (SD, ±12.0). The mean glucose at the end of the fast was 35.7 mg/dl (SD, ±6.5). Thirty-nine patients had sporadic insulinomas, three had multiple endocrine neoplasia type 1 (MEN-1), and three had metastatic recurrent disease. The data from the supervised fast and surgery are summarized in Table 1.

Accuracy of selective pancreatic intraarterial calcium injection for the localization of surgically proven insulinomas

Definite localization was based on resolution of the hypoglycemia after tumor removal for each of the 45 cases. Mean tumor size was 1.8 cm (SD, ±0.9). At surgery, 23 of 45 insulinomas were found in the head, three were found in the neck, 10 were found in the body, eight were found in the tail, and one patient had multiple liver lesions.

The response at the dominant artery correctly predicted tumor location for 38 of 45 (84%) cases. The localization sensitivity of the test was similar for head/neck [22 of 27 (82%)] and body tail lesions [15 of 17 (88%)]. One case with liver metastasis correctly localized to the proper hepatic artery. The median fold increase in stimulated hepatic insulin concentration above baseline at the dominant artery was 7.9-fold (range, 1.5–421.0). Figure 1, *top* and *bottom*, illustrates the variability in the hepatic vein insulin response to calcium injection at the dominant artery for individual cases according to stimulated and unstimulated insulin concentration and fold increase, respectively. This variability may reflect differences in catheter positioning, tumor size, or behavior and/or differences in peripheral insulin sensitivity among cases.

False-negative results

In five patients, calcium injection did not elicit a 2-fold increase in hepatic insulin above baseline. Four tumors localized to the head/neck and one to the body on intraoperative imaging. Technical flaws or anatomical variants could explain two of five cases. In one case, angiography data revealed a technical error of GDA catheterization; the catheter tip was positioned distal to the first branch of the GDA. Injection of calcium at this location would have missed a portion of the pancreatic head. In the second case, angiographic imaging revealed reverse flow back into the common hepatic artery and celiac trunk when the GDA was injected with contrast. This abnormality was suggestive of celiac stenosis and could have led to erroneous results on GDA calcium injection. In theses two cases, the tumor was surgically localized to the pancreatic region supplied by the GDA (case 1 head, overlying the portal vein; case 2 head, to the right of the portal vein).

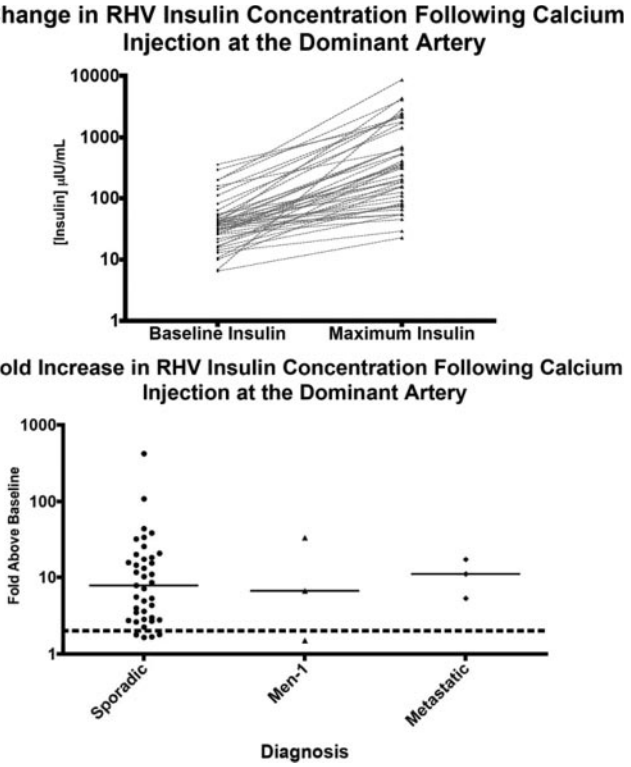

FIG. 1. *Top*, Right hepatic vein (RHV) insulin concentration before and after intraarterial calcium injection at the dominant artery for 45 patients who underwent CaStim at NIH from 1996–2008. *Bottom*, Fold increase in right hepatic vein insulin concentration after calcium injection of dominant artery according to diagnosis. *Dotted line* denotes 2-fold response. *Continuous line* denotes median response for each group.

TABLE 1. Fast, diagnostic, and surgical data from patients undergoing CaStim at NIH from 1996–2008

Supervised fast data (n = 49)	
Fast time, mean (range)	16.5 (0.5–47.5) h
End glucose, mean ± SD	35.7 ± 6.7 mg/dl
End insulin, mean (range)	23.5 (2.3–141.0) µIU/ml
End proinsulin, mean (range)	227.6 (27–810.0) pmol/liter
End C-peptide, mean (range)	4.2 (1.1–16.5) ng/ml
Diagnosis	
Sporadic insulinoma	39/45 (87)
MEN-1 associated insulinoma	3/45 (7)
Metastatic insulinoma	3/45 (7)
Method of resection	
Laparotomy	40/45 (89)
Laparoscopy	5/45 (11)
Type of resection	
Enucleation	35/45 (78)
Distal pancreatectomy	9/45 (20)
Whipple's	1/45 (2)
Surgical localization	
Head and neck	25/45 (56)
Body and tail	18/45 (40)
Liver	1/45 (2)
Not localized	1/45 (2)
Surgical size, mean (range)	1.8 (0.5–5.0) cm

Data are expressed as number of patients/total patients (percentage), unless indicated differently.

False-positive results

Two of 45 surgically proven insulinomas regionalized to the wrong pancreatic region by CaStim. In one, the lesion was found in the head/neck but was predicted to be in the body based on a positive ProxSplenic insulin response to calcium injection. Central tumor necrosis, observed both as calcification on preoperative CT imaging and on review of the surgical specimen, may have affected the response of the tumor to calcium injection. In the second case, the lesion was not in the pancreas proper but was contiguous with the inferior border of the tail. Based on a positive SMA response, it would have been predicted to regionalize to the head/neck region. No technical error or anatomical variant was apparent on review of the angiogram. False-positive localiza-

Downloaded from https://academic.oup.com/jcem/article-abstract/94/4/1074/2596210 by MIT Libraries user on 17 March 2019

tions are particularly problematic because they mislead surgical exploration.

Caveats associated with test interpretation

Interpretation of the results is based on the minimum criterion for a positive response, defined as a 2-fold rise in baseline hepatic insulin after arterial calcium injection. A 2-fold step-up may be seen in more than one artery. In our series, a positive response was observed in a single vessel in 16 of 45 cases (36%) and in two or more vessels in 24 of 45 cases (53%). The reasons for this are unclear and may relate to overlap in arterial territory, tumor behavior, or problems related to the specificity of the test. When multiple arterial injections elicit a positive response, the artery that elicits the dominant response is used to predict tumor localization. The basis for this strategy lies in the fact that most insulinomas are solitary and that the magnitude of the other responses are small in comparison. A graphic representation of a CaStim illustrating this is shown in Fig. 2, *top*.

Other factors besides multiple positive responses that affect result interpretation include presence of anatomical variants and technical errors of catheterization. In these cases, angiography data need to be considered for correct test result interpretation. Unless metastatic disease is strongly suspected, a positive response in the proper hepatic artery should be interpreted with caution. Indeed, inadvertent catheterization of the common, instead of the proper, hepatic artery can lead to a false-positive hepatic regionalization for tumors located in the area supplied by the GDA.

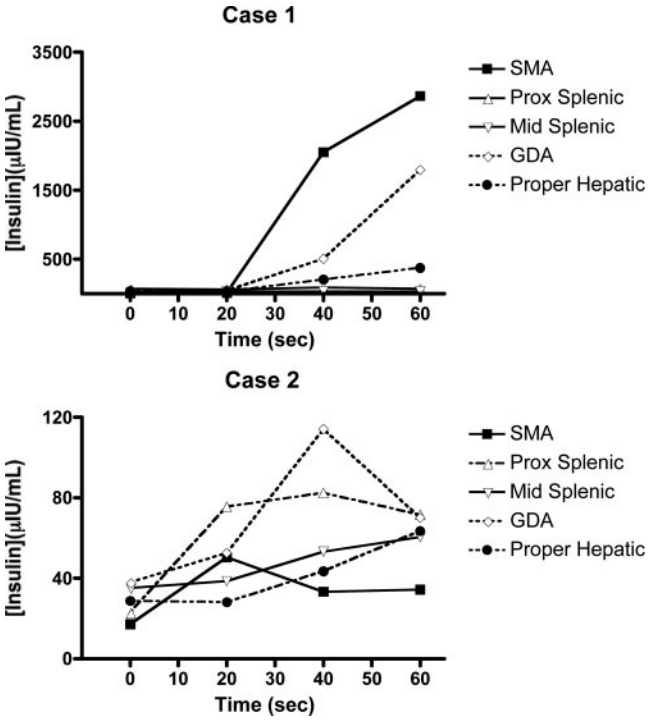

FIG. 2. CaStims with multiple positive responses. A 2-fold step-up may be seen in more than one arterial injection. In case 1, a 421-fold increase in baseline insulin is seen when the SMA is injected, and a 72-fold step-up is seen on injection of the GDA. Both responses regionalize to the head. If a positive response, defined as a greater than 2-fold step-up in baseline insulin, is seen in more than one injected artery, the dominant response is taken as the site of tumor localization. Case 2 illustrates a CaStim with multiple positive responses that fails to regionalize. Positive responses are seen in ProxSplenic (4-fold), SMA (3-fold), and GDA (3-fold). Responses are seen in multiple pancreatic regions (*e.g.* SMA/GDA = head/neck, and ProxSplenic = body/tail), but no clearly dominant response is seen. This CaStim was performed in the work-up of a patient who was later found to be abusing repaglinide surreptitiously (41).

An unusual complication of the procedure

A patient included in the 45 cases was diagnosed with an insulinoma based on documentation of fasting hypoglycemia and coincident hyperinsulinemia during a supervised fast (Table 2). Noninvasive preoperative localization studies were negative. The CaStim regionalized the lesion to the head of the pancreas (15.8-fold increase in right hepatic vein insulin concentration with SMA calcium injection). After the procedure, the patient reported disappearance of hypoglycemic symptoms. A repeat fast showed resolution of both symptomatic hypoglycemia and hyperinsulinemia/proinsulinemia. Amelioration in the patient's symptoms was attributed to a procedural complication hypothesized to have resulted in tumor infarction. After remaining asymptomatic for 2.5 yr, the patient noted symptomatic recurrence. Hypoglycemia, concurrent hyperinsulinemia, and hyperproinsulinemia were again documented on a supervised fast. Fasting data are summarized in Table 2. Noninvasive imaging studies were unrevealing, and the patient was recommended to undergo an exploratory laparotomy. Intraoperative US and bimanual palpation of the pancreas failed to localize the lesion, and surgery was terminated. The patient was managed medically for 2 yr. Despite medical therapy, hypoglycemic episodes persisted and increased in frequency. US, CT, MRI, and octreoscan obtained on a follow-up visit did not identify the lesion. The patient underwent a second CaStim that confirmed the initial regionalization to the head of the pancreas. A second exploratory laparotomy resulted in identification of a 1.5-cm insulinoma in the pancreatic head. Enucleation of the lesion was curative, and the patient has had no symptomatic recurrence. No hypoglycemic, hypercalcemic, allergic, bleeding, thrombotic, renal, or other complications were noted for the other cases.

Accuracy of noninvasive imaging for the localization of insulinomas

US correctly localized six of 43 (14%) sporadic insulinoma cases. US failed to localize the MEN-1 and two metastatic insulinoma cases. CT and MRI correctly localized 14 of 44 (32%) and 11 of 44 (25%) of the sporadic insulinoma cases, respectively, but failed to localize the MEN-1 and two out of three metastatic insulinoma cases. These results are summarized in Table 3.

TABLE 2. Summary of three supervised fast results for unusual case

	Initial	After CaStim	After symptomatic recurrence
Duration (h)	5	48	8
Glucose (mg/dl)	39	71	39
Insulin (μIU/ml)	5.6	<2.0	5.4
C-peptide (ng/ml)	3.2	1.6	3.1
Proinsulin (pmol/liter)	45	9.8	60
Sulfonylurea screen	Negative	Negative	Negative

Downloaded from https://academic.oup.com/jcem/article-abstract/94/4/1074/2596210 by MIT Libraries user on 17 March 2019

TABLE 3. Accuracy of localization studies for surgically proven insulinoma at NIH 1996–2008 according to diagnosis

	Sporadic	MEN-1	Metastatic	Total (%)
CaStim				
Correct localization	33/39 (84)	2/3 (67)	3/3 (100)	84
False-negative localization	4/39 (11)	1/3 (33)	0/3	11
False-positive localization	2/39 (5)	0/3	0/3	4
US				
Correct localization	6/38 (16)	0/3 (0)	0/2 (0)	14
False-negative localization	27/38 (71)	2/3 (67)	2/2 (100)	72
False-positive localization	5/38 (13)	1/3 (33)	0/2 (0)	14
CT				
Correct localization	13/38 (35)	0/3 (0)	1/3 (33)	32
False-negative localization	19/38 (49)	1/3 (33)	2/3 (67)	50
False-positive localization	6/38 (16)	2/3 (67)	0/3 (0)	18
MRI				
Correct localization	11/38 (30)	0/3 (0)	0/3 (0)	25
False-negative localization	22/38 (57)	2/3 (67)	3/3 (100)	61
False-positive localization	5/38 (13)	1/3 (33)	0/3 (0)	14

Data are expressed as number of patients/total patients (percentage), unless indicated differently.

Two of the three MEN-1 patients studied had multiple pancreatic lesions seen on cross-sectional imaging. Results from these could not differentiate insulinoma from noninsulinoma lesions and were therefore considered nonlocalizing. Despite advances in cross-sectional imaging techniques, noninvasive imaging remains an insensitive localizing tool (13, 14, 19).

Discussion

Selective pancreatic intraarterial calcium injection, to localize insulin-producing islet cell tumors, was developed and has been used at NIH since 1989. Outside of the insulinoma setting, the test has also been used to localize insulin-secreting lesions associated with congenital hyperinsulinism (20–22). The specificity of this test in the setting of insulinoma is based on the assumptions that: the tumor will have a dominant arterial supply, calcium elicits a unique response on tumor cells, and normal β-cell function is suppressed relative to tumor cells. In the last 19 yr, 81 patients referred to NIH with nonlocalizing disease were studied with this technique. A review of the first 36 cases estimated this technique to be 88–94% sensitive for predicting tumor location (14, 15). Since then, an additional 45 cases have been studied, and we report a localization sensitivity of 84%. Our findings are consistent with other published reports (23–26). Despite advances in CT and MR technology, CaStim has remained vastly superior to these noninvasive imaging modalities as a preoperative localizing tool for insulin-producing islet cell tumors. The success of nuclear imaging techniques using radiolabeled dihydroxyphenylalanine (27) or the glucagon-like peptide-1 analog exendin-4 (28) is promising and will need to be confirmed in larger series.

Invasive tests should be reserved for cases that do not localize by conventional noninvasive means, and the choice of test should be guided by local availability and expertise. In many centers, EUS is the preferred preoperative test for cases that fail to localize by conventional imaging. Shortcomings of EUS are similar to CaStim and include: invasiveness, requirement for sedation, operator and center dependence, and lack of universal availability. The published localization sensitivity of EUS for insulinomas is similar to CaStim with an overall sensitivity of 80% that ranges from 57–94% (29–35). In contrast to CaStim, reported sensitivity of EUS for pancreatic tail lesions is lower and ranges from 37–50% (29, 33). EUS does not provide functional information, and pancreatic nodularities mistaken for insulinomas on EUS have been reported to affect the specificity of this test (36). Because our data were derived from a referred patient population, a bias toward an underestimation of the sensitivity of noninvasive localization procedures is likely. The preoperative localization sensitivity for each of the different modalities used at NIH from 1989–2008 is summarized in Fig. 3.

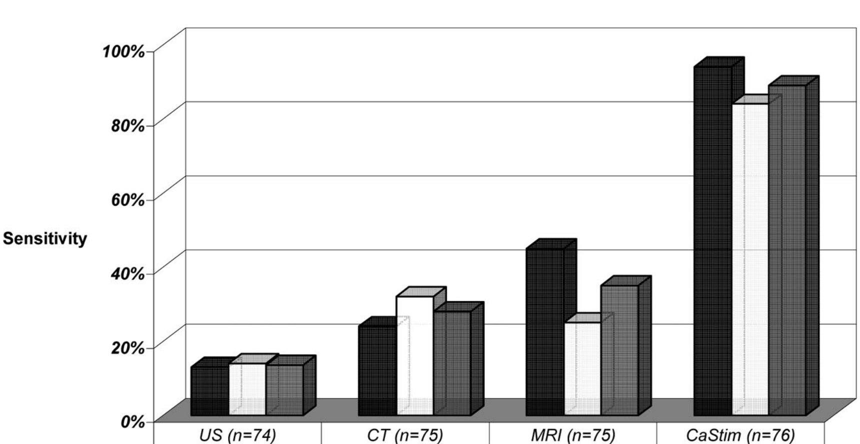

	US (n=74)	CT (n=75)	MRI (n=75)	CaStim (n=76)
1989-1996	13%	24%	45%	94%
1996-2008	14%	32%	25%	84%
Combined	14%	28%	35%	89%

Modality

FIG. 3. Localization sensitivity of preoperative tests.

Downloaded from https://academic.oup.com/jcem/article-abstract/94/4/1074/2596210 by MIT Libraries user on 17 March 2019

With the advent of highly sensitive operative localization techniques, debate over the value of preoperative localization in the setting of insulinoma exists. As our five false-negative cases illustrate, negative preoperative localization does not change the treatment course, and in these cases operative localization remains the ultimate form of localization. In our patient population, however, cases localized by CaStim but not by operative imaging have been reported (14, 19). The accuracy of intraoperative US and laparoscopic US were 86% (14) and 88% (19), respectively, at localizing insulinomas. The information gained from CaStim and operative imaging studies differ, making these tests complementary. In the former, localization is based on the functional property of the tumor, and in the latter on its direct visualization. With the use of these two localization modalities, the majority of cases are localized, the chance for a successful initial surgical resection is maximized, and the likelihood of reoperation with its associated morbidity is minimized.

Preoperative localization may become more important in the era of laparoscopic surgery. Use of laparoscopic surgery for the treatment of insulinomas has become widely available (19, 37–40). In the time period covered by this report, removal by laparoscopy was attempted in 21 of the 45 patients who underwent surgery. Only five (24%) insulinomas were removed by laparoscopy, and these were all in the pancreatic body/tail region. Conversion to open laparotomy was used for the remainder of the cases because problems related to either tumor access or tumor proximity to specific pancreatic structures precluded laparoscopic resection (19). As the procedure is refined and technical expertise improves, the success rate of laparoscopic removal will increase. For some lesions, however, laparotomy will remain the preferred procedure. In this setting, preoperative tools such as the CaStim may play a prominent role to define lesions amenable to laparoscopic removal *vs.* those that are not.

Several caveats make interpreting the accuracy of the CaStim in the setting of MEN-1 difficult. In contrast to sporadic cases, MEN-1 cases usually have multiple adenomas, and delineation of the insulinoma among these multiple tumors is difficult. The surgery usually recommended for these cases is a distal pancreatectomy. If there is preoperative localization to the head, however, a careful intraoperative exploration of this region to identify and enucleate the functional adenoma is indicated. Intraoperative needle aspiration of these lesions for insulin may be used to facilitate this process.

Over the last 17 yr, our large experience reveals the CaStim to be a reliable and sensitive means to localize insulinomas in adults. In this setting, resolution of symptoms after removal of a solitary lesion allows for unequivocal confirmation of the functional tumor's localization. This "gold standard" is used to evaluate the accuracy of preoperative localization and would be lacking if the disease were multifocal or diffuse. In adults, the utility of the test outside of insulinoma and for purposes other than localization is presently unknown. In contemplating the use of this test, it is important to recognize that the diagnostic value of the test remains uncharacterized. Indeed, most of the published experience with the CaStim in adults is limited to patients with a preestablished diagnosis of insulinoma. More importantly, the normative response and factors that influence this response have not been established. The assumption that a greater than 2-fold rise in hepatic insulin after intraarterial calcium injection represents a pathological β-cell process needs to be tested before attributing any diagnostic value to the test. The diffusely positive CaStim response (Fig. 2, case 2) in our previously published repaglinide case (41) and in another published case of hypoglycemia caused by surreptitious sulfonylurea administration (42) calls the diagnostic specificity of this test into question. Until the normative response is established, caution should be exercised if one is to rely on the diagnostic value of the test to guide therapeutic recommendations.

The growth of interventional radiology has made the CaStim widely available, and it is important for the endocrinologist to appreciate both the utility and the caveats associated with this procedure.

Acknowledgments

We are grateful to the nursing staff and physicians of the clinical center who cared for the patients presented in this report.

Address all correspondence and requests for reprints to: Jean-Marc Guettier, National Institutes of Health, Building 10, CRC 6-5940, Bethesda, Maryland 20892. E-mail: guettierj@mail.nih.gov.

Disclosure Summary: J.-M.G., A.K., R.C., M.C.S., C.C., H.R.A., S.K.L., J.F.P., and P.G. have nothing to disclose.

References

1. **Service FJ, McMahon MM, O'Brien PC, Ballard DJ** 1991 Functioning insulinoma—incidence, recurrence, and long-term survival of patients: a 60-year study. Mayo Clin Proc 66:711–719
2. **Oberg K, Eriksson B** 2005 Endocrine tumours of the pancreas. Best Pract Res Clin Gastroenterol 19:753–781
3. **Service FJ, Dale AJ, Elveback LR, Jiang NS** 1976 Insulinoma: clinical and diagnostic features of 60 consecutive cases. Mayo Clin Proc 51:417–429
4. **Hirshberg B, Livi A, Bartlett DL, Libutti SK, Alexander HR, Doppman JL, Skarulis MC, Gorden P** 2000 Forty-eight-hour fast: the diagnostic test for insulinoma. J Clin Endocrinol Metab 85:3222–3226
5. **Grama D, Eriksson B, Martensson H, Cedermark B, Ahren B, Kristoffersson A, Rastad J, Oberg K, Akerstrom G** 1992 Clinical characteristics, treatment and survival in patients with pancreatic tumors causing hormonal syndromes. World J Surg 16:632–639
6. **Hirshberg B, Libutti SK, Alexander HR, Bartlett DL, Cochran C, Livi A, Chang R, Shawker T, Skarulis MC, Gorden P** 2002 Blind distal pancreatectomy for occult insulinoma, an inadvisable procedure. J Am Coll Surg 194:761–764
7. **Norton JA, Shawker TH, Doppman JL, Miller DL, Fraker DL, Cromack DT, Gorden P, Jensen RT** 1990 Localization and surgical treatment of occult insulinomas. Ann Surg 212:615–620
8. **Doherty GM, Doppman JL, Shawker TH, Miller DL, Eastman RC, Gorden P, Norton JA** 1991 Results of a prospective strategy to diagnose, localize, and resect insulinomas. Surgery 110:989–996; discussion, 996–987
9. **Pasieka JL, McLeod MK, Thompson NW, Burney RE** 1992 Surgical approach to insulinomas. Assessing the need for preoperative localization. Arch Surg 127:442–447
10. **Thompson GB, Service FJ, van Heerden JA, Carney JA, Charboneau JW, O'Brien PC, Grant CS** 1993 Reoperative insulinomas, 1927 to 1992: an institutional experience. Surgery 114:1196–1204; discussion, 1205–1196
11. **Doppman JL, Miller DL, Chang R, Shawker TH, Gorden P, Norton JA** 1991 Insulinomas: localization with selective intraarterial injection of calcium. Radiology 178:237–241
12. **Doppman JL, Miller DL, Chang R, Gorden P, Eastman RC, Norton JA** 1993 Intraarterial calcium stimulation test for detection of insulinomas. World J Surg 17:439–443
13. **Doppman JL, Chang R, Fraker DL, Norton JA, Alexander HR, Miller DL, Collier E, Skarulis MC, Gorden P** 1995 Localization of insulinomas to regions

Downloaded from https://academic.oup.com/jcem/article-abstract/94/4/1074/2596210 by MIT Libraries user on 17 March 2019

of the pancreas by intra-arterial stimulation with calcium. Ann Intern Med 123:269–273
14. **Brown CK, Bartlett DL, Doppman JL, Gorden P, Libutti SK, Fraker DL, Shawker TH, Skarulis MC, Alexander HR** 1997 Intraarterial calcium stimulation and intraoperative ultrasonography in the localization and resection of insulinomas. Surgery 122:1189–1193; discussion, 1193–1194
15. **Gaeke RF, Kaplan EL, Rubenstein A, Starr J, Burke G** 1975 Insulin and proinsulin release during calcium infusion in a patient with islet-cell tumor. Metabolism 24:1029–1034
16. **Kaplan EL, Rubenstein AH, Evans R, Lee CH, Klementschitsch P** 1979 Calcium infusion: a new provocative test for insulinomas. Ann Surg 190:501–507
17. **Brunt LM, Veldhuis JD, Dilley WG, Farndon JR, Santen RJ, Leight GS, Wells Jr SA** 1986 Stimulation of insulin secretion by a rapid intravenous calcium infusion in patients with β-cell neoplasms of the pancreas. J Clin Endocrinol Metab 62:210–216
18. **Kato M, Doi R, Imamura M, Okada N, Shimada Y, Hosotani R, Miyazaki JI** 1998 Response of human insulinoma cells to extracellular calcium is different from normal B cells. Dig Dis Sci 43:2429–2438
19. **Grover AC, Skarulis M, Alexander HR, Pingpank JF, Javor ED, Chang R, Shawker T, Gorden P, Cochran C, Libutti SK** 2005 A prospective evaluation of laparoscopic exploration with intraoperative ultrasound as a technique for localizing sporadic insulinomas. Surgery 138:1003–1008; discussion, 1008
20. **Ferry Jr RJ, Kelly A, Grimberg A, Koo-McCoy S, Shapiro MJ, Fellows KE, Glaser B, Aguilar-Bryan L, Stafford DE, Stanley CA** 2000 Calcium-stimulated insulin secretion in diffuse and focal forms of congenital hyperinsulinism. J Pediatr 137:239–246
21. **De Leon DD, Stanley CA** 2007 Mechanisms of disease: advances in diagnosis and treatment of hyperinsulinism in neonates. Nat Clin Pract Endocrinol Metab 3:57–68
22. **Lovvorn 3rd HN, Nance ML, Ferry Jr RJ, Stolte L, Baker L, O'Neill Jr JA, Schnaufer L, Stanley CA, Adzick NS** 1999 Congenital hyperinsulinism and the surgeon: lessons learned over 35 years. J Pediatr Surg 34:786–792; discussion, 792–793
23. **O'Shea D, Rohrer-Theurs AW, Lynn JA, Jackson JE, Bloom SR** 1996 Localization of insulinomas by selective intraarterial calcium injection. J Clin Endocrinol Metab 81:1623–1627
24. **Brandle M, Pfammatter T, Spinas GA, Lehmann R, Schmid C** 2001 Assessment of selective arterial calcium stimulation and hepatic venous sampling to localize insulin-secreting tumours. Clin Endocrinol (Oxf) 55:357–362
25. **Wiesli P, Brandle M, Schmid C, Krahenbuhl L, Furrer J, Keller U, Spinas GA, Pfammatter T** 2004 Selective arterial calcium stimulation and hepatic venous sampling in the evaluation of hyperinsulinemic hypoglycemia: potential and limitations. J Vasc Interv Radiol 15:1251–1256
26. **Won JG, Tseng HS, Yang AH, Tang KT, Jap TS, Kwok CF, Lee CH, Lin HD** 2003 Intra-arterial calcium stimulation test for detection of insulinomas: detection rate, responses of pancreatic peptides, and its relationship to differentiation of tumor cells. Metabolism 52:1320–1329
27. **Kauhanen S, Seppanen M, Minn H, Gullichsen R, Salonen A, Alanen K, Parkkola R, Solin O, Bergman J, Sane T, Salmi J, Valimaki M, Nuutila P** 2007 Fluorine-18-L-dihydroxyphenylalanine (18F-DOPA) positron emission tomography as a tool to localize an insulinoma or β-cell hyperplasia in adult patients. J Clin Endocrinol Metab 92:1237–1244
28. **Wild D, Macke H, Christ E, Gloor B, Reubi JC** 2008 Glucagon-like peptide 1-receptor scans to localize occult insulinomas. N Engl J Med 359:766–768
29. **Ardengh JC, Rosenbaum P, Ganc AJ, Goldenberg A, Lobo EJ, Malheiros CA, Rahal F, Ferrari AP** 2000 Role of EUS in the preoperative localization of insulinomas compared with spiral CT. Gastrointest Endosc 51:552–555
30. **Glover JR, Shorvon PJ, Lees WR** 1992 Endoscopic ultrasound for localisation of islet cell tumours. Gut 33:108–110
31. **Rosch T, Lightdale CJ, Botet JF, Boyce GA, Sivak Jr MV, Yasuda K, Heyder N, Palazzo L, Dancygier H, Schusdziarra V, et al** 1992 Localization of pancreatic endocrine tumors by endoscopic ultrasonography. N Engl J Med 326:1721–1726
32. **Pitre J, Soubrane O, Palazzo L, Chapuis Y** 1996 Endoscopic ultrasonography for the preoperative localization of insulinomas. Pancreas 13:55–60
33. **Schumacher B, Lubke HJ, Frieling T, Strohmeyer G, Starke AA** 1996 Prospective study on the detection of insulinomas by endoscopic ultrasonography. Endoscopy 28:273–276
34. **Gouya H, Vignaux O, Augui J, Dousset B, Palazzo L, Louvel A, Chaussade S, Legmann P** 2003 CT, endoscopic sonography, and a combined protocol for preoperative evaluation of pancreatic insulinomas. AJR Am J Roentgenol 181:987–992
35. **Zimmer T, Scherubl H, Faiss S, Stolzel U, Riecken EO, Wiedenmann B** 2000 Endoscopic ultrasonography of neuroendocrine tumours. Digestion 62(Suppl 1):45–50
36. **Kann PH, Wirkus B, Keth A, Goitom K** 2003 Pitfalls in endosonographic imaging of suspected insulinomas: pancreatic nodules of unknown dignity. Eur J Endocrinol 148:531–534
37. **Fernandez-Cruz L, Martinez I, Cesar-Borges G, Astudillo E, Orduna D, Halperin I, Sesmilo G, Puig M** 2005 Laparoscopic surgery in patients with sporadic and multiple insulinomas associated with multiple endocrine neoplasia type 1. J Gastrointest Surg 9:381–388
38. **Mabrut JY, Fernandez-Cruz L, Azagra JS, Bassi C, Delvaux G, Weerts J, Fabre JM, Boulez J, Baulieux J, Peix JL, Gigot JF** 2005 Laparoscopic pancreatic resection: results of a multicenter European study of 127 patients. Surgery 137:597–605
39. **Ammori BJ, El-Dhuwaib Y, Ballester P, Augustine T** 2005 Laparoscopic distal pancreatectomy for neuroendocrine tumors of the pancreas. Hepatogastroenterology 52:620–624
40. **Sweet MP, Izumisato Y, Way LW, Clark OH, Masharani U, Duh Q-Y** 2007 Laparoscopic enucleation of insulinomas. Arch Surg 142:1202–1204
41. **Hirshberg B, Skarulis MC, Pucino F, Csako G, Brennan R, Gorden P** 2001 Repaglinide-induced factitious hypoglycemia. J Clin Endocrinol Metab 86:475–477
42. **Manning PJ, Espiner EA, Yoon K, Drury PL, Holdaway IM, Bowers A** 2003 An unusual cause of hyperinsulinaemic hypoglycaemia syndrome. Diabet Med 20:772–776

Downloaded from https://academic.oup.com/jcem/article-abstract/94/4/1074/2596210 by MIT Libraries user on 17 March 2019
