# Supplementary material for: Diagnostic value of ASVS for insulinoma localization: A systematic review and meta-analysis
Source: PLoS One. 2019 Nov 19;14(11):e0224928. doi: 10.1371/journal.pone.0224928 (PMC6863549; doi:10.1371/journal.pone.0224928)
Supplement: S2 File — (ZIP) [file pone.0224928.s002.zip › included studies/localization of insulonoma.pdf]

Localization of Insulinomas to Regions of the Pancreas by Intra-arterial Stimulation with Calcium

John L. Doppman, MD; Richard Chang, MD; Douglas L. Fraker, MD; Jeffrey A. Norton, MD; H. Richard Alexander, MD; Donald L. Miller, MD; Elaine Collier, MD; Monica C. Skarulis, MD; and Phillip Gorden, MD

■ *Objective:* To determine the sensitivity of calcium injected into pancreatic arteries in localizing insulin-secreting tumors to regions of the pancreas.

■ *Design and Patients:* To stimulate the release of insulin, 25 patients with surgically proven insulinomas (average diameter, 15 mm) had calcium gluconate (0.025 mEq Ca^{++}/kg body weight) injected before surgery into the arteries supplying the pancreatic head (gastroduodenal and superior mesenteric arteries) and the body and tail (splenic artery) of the pancreas.

■ *Setting:* Tertiary referral hospital.

■ *Measurements:* Insulin levels were measured in samples taken from the right and left hepatic veins before and 30, 60, and 120 seconds after calcium injection. A twofold increase in insulin level in the sample taken from the right hepatic vein 30 or 60 seconds after injection localized the insulinoma to the segment of the pancreas supplied by the selectively injected artery. Localization done using calcium stimulation was compared with localization done using transcutaneous ultrasonography (n = 22), computed tomography (n = 23), magnetic resonance imaging (n = 21), arteriography (n = 25), and portal venous sampling (n = 9).

■ *Results:* Calcium stimulation localized 22 of 25 insulinomas (sensitivity, 88% [95% CI, 68% to 97%]) to the correct region of the pancreas. The sensitivities of the other imaging methods were 9% for ultrasonography (CI, 1% to 23%), 17% for computed tomography (CI, 5% to 39%), 43% for magnetic resonance imaging (CI, 22% to 66%), 36% for arteriography (CI, 18% to 57%), and 67% for portal venous sampling (CI, 30% to 93%). Calcium stimulation added only a few minutes to the time needed for pancreatic arteriography and caused no morbid conditions.

■ *Conclusion:* Intra-arterial calcium stimulation with right hepatic vein sampling for insulin gradients is the most sensitive preoperative test for localizing insulinomas.

Ann Intern Med. 1995;123:269-273.

From the National Institutes of Health and the National Naval Medical Center, Bethesda, Maryland, and Washington University, St. Louis, Missouri. For current author addresses, see end of text.

Despite the introduction of sophisticated cross-sectional imaging techniques—computed tomography, magnetic resonance imaging, and ultrasonography—the localization of insulinomas smaller than 2 cm remains a problem. In our previous experience (1), these noninvasive methods of localization had sensitivities of 17% (computed tomography), 25% (magnetic resonance imaging), and 26% (ultrasonography). Our results may have been biased because most patients have negative results on noninvasive imaging studies before referral to the National Institutes of Health. Of the invasive localization techniques, pancreatic arteriography visualized 35% of small (<2 cm) insulinomas. The success of portal venous sampling does not depend on tumor size, and this method localized insulinomas in 77% of patients. However, percutaneous portal venous sampling requires special skills and experience and is associated with slight but significant morbidity (2). We have developed a technique with which one can localize insulinomas before surgery by stimulating the release of insulin using selective intra-arterial injections of calcium gluconate as a secretagogue and then measuring insulin levels in the right hepatic vein. The results in our first 9 patients were promising (3, 4), and we have since studied an additional 16 patients with surgically proven insulinomas. We present the results of arterial stimulation and venous sampling in these 25 patients studied over the past 4 years.

Methods

Diagnosis of insulin-secreting islet cell tumor was based on the development of symptomatic hypoglycemia (blood glucose level, <40 mg/100 mL) with inappropriate plasma insulin levels during prolonged fasting. Ten of the patients were men and 15 were women; their average age was 43 years (range, 24 to 72 years). Five patients had had previous unsuccessful explorations of the pancreas, and 3 had had distal pancreatectomy during these explorations. Two patients had multiple endocrine neoplasia type I.1;0

Most of the 25 patients had had computed tomography (n = 23), magnetic resonance imaging (n = 21), and ultrasonography (n = 22) before having arteriography with calcium stimulation. The first 9 patients had portal venous sampling, but this procedure was not done in the other 16 patients because analysis showed that calcium stimulation provided similar information with less morbidity. This decision was supported by the similar sensitivities of portal venous sampling and intra-arterial secretin stimulation in our patients with the Zollinger–Ellison syndrome (5, 6).

Computed tomography (done using a 9800 HiLite, General

For editorial comment, see pp 311–312.

© 1995 American College of Physicians

Downloaded From: http://annals.org/ by a Penn State University Hershey User on 06/26/2015

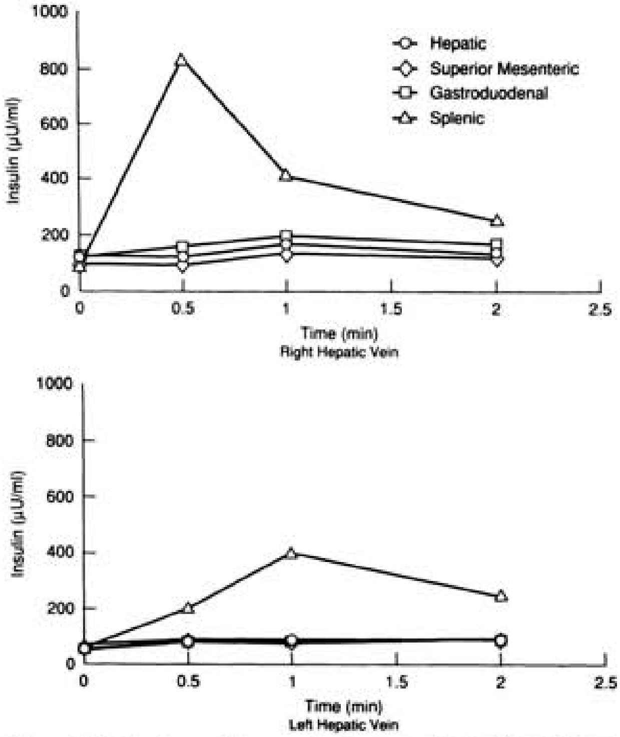

Figure 1. Typical sampling results from a patient with an insulinoma in the pancreatic tail. Note the eightfold gradient at the 30-second sample from the right hepatic vein (*top*) and the rapid decrease in insulin levels at the 60-second sample; this is a frequent, but not invariable, pattern. Lesser gradients are measured in the samples from the left hepatic vein (*bottom*). The conversion factor for insulin levels into pmol/L is 7.175.

Electric, Milwaukee, Wisconsin) was done with 5-mm contiguous sections through the pancreas during the bolus injection of 130 mL of iodinated contrast material (iopamidol [Isovue 300, Bristol-Myers Squibb, Princeton, New Jersey]) at 2 mL per second. Magnetic resonance imaging was done using a 0.5-Tesla scanner (Picker, Highland Heights, Ohio) with 10-mm thick axial T1-weighted (repetition time [TR]/echo time [TE] = 300/10) and short inversion time inversion recovery (STIR) (TR/TI [inversion time]/TE = 1800-2200/100/30) sequences. Gadopentetate dimeglumine (Magnevist, Berlex Lab, Wayne, New Jersey) was not given. Ultrasonography was done using a 3.5- or 5-MHz phased-array sector transducer (Acuson, Mountain View, California).

Pancreatic arteriography was done by selectively injecting nonionic contrast agent (Isovue 300) into the gastroduodenal, splenic, and superior mesenteric arteries. Care was taken to position the catheter at the origin of these vessels so that major pancreatic arteries originating proximally from these vessels, such as the dorsal pancreatic and pancreatic magna arteries, would be perfused. Selective arteriography of the dorsal pancreatic and pancreatic magna arteries was occasionally done, but we did not infuse calcium into these small pancreatic branches because we feared that doing so might increase the risk for pancreatitis. After each selective arteriogram, calcium gluconate 10% (Lyphomed, Rosemont, Illinois), diluted with saline to a volume of 5 mL, was injected into the selectively catheterized artery at a dose of 0.025 mEq Ca^{++}/kg body weight. Blood samples (5 mL) for insulin determination were obtained from the right (n = 25) and left (n = 17) hepatic veins before and 30, 60, and 120 seconds after calcium infusion. Specimens from the hepatic veins were placed on ice, and plasma was separated in a refrigerated centrifuge and stored at −20 °C until insulin levels were measured by radioimmunoassay.

Samples were obtained from the left as well as the right hepatic vein in the first 17 patients because of concern that an insulinoma in the body or tail of the pancreas might be overlooked if splenic venous effluent streamed into the left hepatic lobe. However, it is more difficult to place and maintain a catheter in the left than in the right hepatic vein. To determine whether diagnostic elevations of insulin levels were ever seen only in the left hepatic vein, we compared insulin levels in the right and left hepatic veins in a subset of 10 patients whose insulinomas were in the pancreatic body and tail.

The insulinomas ranged in size from 6 to 25 mm (average, 15 mm). Twelve were located to the right of the superior mesenteric artery (pancreatic head and neck), and 13 were located to the left (pancreatic body and tail). All tumors of the head and neck were enucleated. Tumors of the body and tail were removed by enucleation (n = 5) or distal pancreatectomy (n = 8). Intraoperative ultrasonography (10-MHz transducer, Diasonics, Santa Clara, California) was done in each patient to visualize the tumor, to identify major pancreatic and biliary ducts adjacent to the tumor, and to direct the pancreatic incision for enucleation. All patients were cured.

Data Analysis

The results of sampling from the right (n = 25) and left (n = 17) hepatic veins were plotted for each patient. Graphs were analyzed by selecting the greatest insulin response in a given vessel in the 30- or 60-second sample after injection. Each patient was coded so that, at the time of analysis, the observers were unaware of the results of any other localizing studies or of the location of the tumor at surgery. A response after calcium infusion into the gastroduodenal or superior mesenteric artery localized the adenoma to the head and neck of the pancreas; a response after splenic artery injection localized the adenoma to the body and tail of the pancreas. A response to calcium stimulation usually involved a single artery (Figure 1). When both the gastroduodenal and superior mesenteric arteries showed a response to calcium stimulation, the insulinoma was presumed to

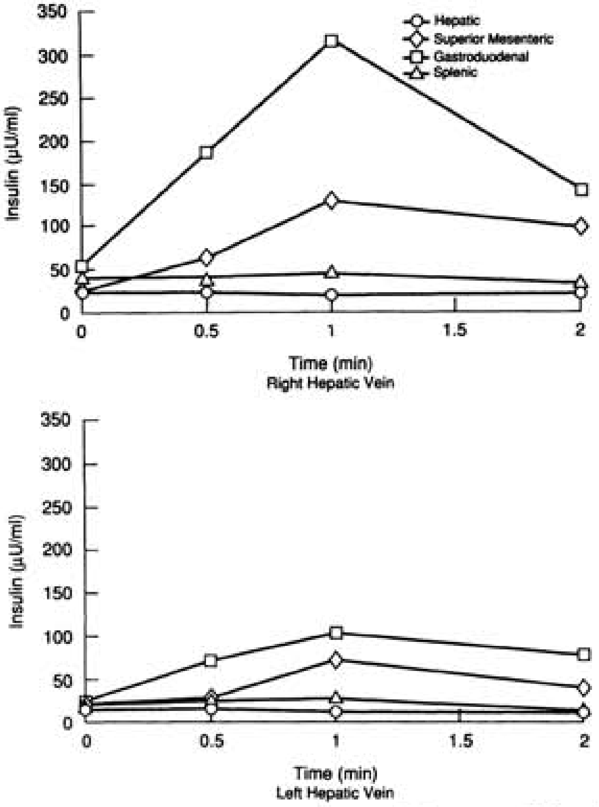

Figure 2. In a patient with an insulinoma of the pancreatic head, greater than twofold gradients were seen after calcium injection into both the gastroduodenal and superior mesenteric arteries, with higher elevations in the gastroduodenal artery (*top*). Although the gradients in the left hepatic vein (*bottom*) are substantial, the elevations of insulin levels in the right hepatic vein are higher (sixfold) than those in the left hepatic vein (fourfold). The conversion factor for insulin levels into pmol/L is 7.175.

Downloaded From: http://annals.org/ by a Penn State University Hershey User on 06/26/2015

be located to the right of the superior mesenteric artery (pancreatic head and neck) (Figure 2). When no vessel was clearly dominant, the response was considered nonlocalizing (Figure 3).

The sensitivity of calcium stimulation in all 25 patients was calculated and compared with the sensitivity of the noninvasive imaging studies (computed tomography, magnetic resonance imaging, and ultrasonography) and arteriography. Specificity was irrelevant because all patients in the series had proven insulinomas. In the 9 patients who had portal venous sampling, the sensitivity of calcium stimulation was compared with the sensitivity of portal venous sampling.

To determine whether it was necessary to sample the left hepatic vein, we compared the maximum insulin levels in the right and the left hepatic veins and the ratio of insulin levels in the hepatic vein with those in the peripheral vein in a subset of 10 patients with insulinomas of the body and tail.

Results

The results of all localization studies are summarized in Table 1. A response to calcium stimulation—that is, a greater than twofold elevation of insulin levels in the right or left hepatic vein on the 30- or 60-second samples—occurred in all 25 patients. Calcium stimulation with venous sampling correctly predicted the site of the insulinoma in 22 of 25 patients (sensitivity, 88% [95% CI, 68% to 97%]). In 2 of the 3 patients with false localizations, responses to gastroduodenal and splenic artery injections occurred in the presence of a tumor in the proximal body of the pancreas (Figure 3); in the third patient, a response to a superior mesenteric artery injection occurred in the presence of a tumor in the proximal body. All patients who had a positive response to splenic artery injection only had insulinomas of the body or tail. Two of the three false localizations occurred in our first 5 patients; only one false localization occurred among our last 20 patients.

In the nine patients who had both portal venous sampling and calcium stimulation, portal venous sampling correctly localized six insulinomas (sensitivity, 67%), and calcium stimulation correctly localized seven insulinomas (sensitivity, 78%).

Among 10 patients with surgically proven insulinomas of the body and tail of the pancreas, the maximum insulin levels in response to calcium stimulation were higher in the right than in the left hepatic vein in 8 patients and were equal in the right and left hepatic veins in 1 patient (103 μU/mL compared with 107 μU/mL [739 pmol/L compared with 768 pmol/L]). Only 1 patient with an insulinoma of the pancreatic body had a higher insulin level in the left than in the right hepatic vein (148 μU/L compared with 108 μU/mL [1062 pmol/L compared with 775 pmol/L]), but the ratio of the insulin level in the hepatic vein to the insulin level in the peripheral vein was diagnostic in both the right (6.7) and the left (4.7) hepatic veins. Nine of the 10 patients had higher ratios in the right hepatic vein samples (Table 2). No insulinoma of the body or tail would have been missed by sampling only the right hepatic vein.

Table 1. Results of Localization Studies in 25 Patients with Surgically Proven Insulinomas

Test	Insulinomas Correctly Localized, *n/n*	Insulinomas Correctly Localized, % (95% CI)
Computed tomography	4/23	17 (5 to 39)
Ultrasonography	2/22	9 (1 to 23)
Magnetic resonance imaging	9/21	43 (22 to 66)
Arteriography	9/25	36 (18 to 57)
Portal venous sampling	6/9	67 (30 to 93)
Calcium stimulation	22/25	88 (68 to 97)

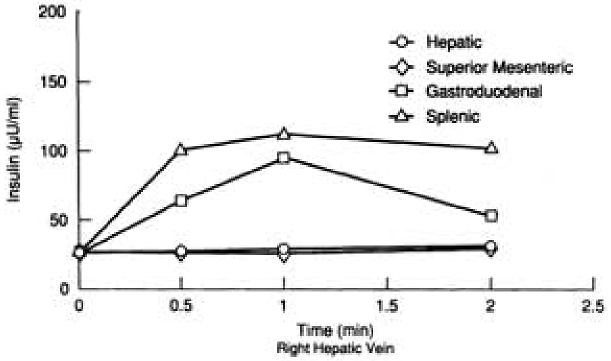

Figure 3. The only nondiagnostic study in the last 20 cases shows elevated insulin levels in the splenic and gastroduodenal arteries. Although the 30-second gradient is higher in the splenic vein, this is considered a nonlocalizing study. The tumor was found in the body of the pancreas to the left of the superior mesenteric artery. The conversion factor for insulin levels into pmol/L is 7.175.

Aside from a mild and transient sensation of warmth in the epigastrium during calcium infusion, no complications occurred. Neither measurable hypercalcemia (in the first 5 patients) nor symptomatic hypoglycemia were encountered.

Calcium stimulation with venous sampling was done in two patients not included in this series. In one patient with multiple endocrine neoplasia type I who had had distal pancreatectomy for an insulinoma of the pancreatic tail, recurrent hypoglycemia with a tumor of the pancreatic head was seen on computed tomography, magnetic resonance imaging, and arteriography. The results of a calcium stimulation test and portal venous sampling were also positive for an insulinoma of the head of the pancreas. The patient elected not to have surgery, and the hypoglycemia was controlled medically. In the other patient, the calcium stimulation test was interrupted by equipment failure after only a single vessel (the gastroduodenal artery) was injected (negative response). The results of subsequent portal venous sampling were positive in the splenic vein samples. Arteriography with calcium stimulation was not repeated. A 17-mm insulinoma was resected from the pancreatic tail.

Discussion

In this series of 25 patients with surgically proven insulinomas, calcium stimulation with hepatic venous sampling was the most sensitive of the preoperative localizing studies (22 of 25 [88%]). Arteriographic demonstration of a tumor was not necessary for a positive response to a selective calcium infusion into the same vessel. In 13 of 16 patients with negative arteriograms, the injection of calcium into the same vessel correctly localized an adenoma by the insulin response in hepatic vein samples. Calcium stimulation with hepatic venous sampling was similar to

Downloaded From: http://annals.org/ by a Penn State University Hershey User on 06/26/2015

Table 2. Insulin Levels in Hepatic and Peripheral Veins*

Measurement	Value
Maximum insulin levels, *μU/mL*	
Right hepatic vein	1073 ± 858
Left hepatic vein	563 ± 465
Maximum ratio of insulin levels in the hepatic vein to those in the peripheral vein	
Right hepatic vein	14.4 ± 14
Left hepatic vein	8.9 ± 5.9

* Values are given ± SD. The conversion factor for insulin levels into pmol/L is 7.175. Levels were measured in 10 patients with proven insulinomas of the pancreatic body and tail.

portal venous sampling, which was thought not to be clinically indicated after the results in our first 9 patients were reviewed (4). This is also true in our larger experience with gastrin-producing islet cell tumors (5, 6); we have abandoned portal vein sampling in patients with the Zollinger–Ellison syndrome because the procedure is more invasive and painful and is no more sensitive than intra-arterial secretin stimulation (7).

Referral of patients to the National Institutes of Health introduces a bias because a selection toward occult tumors takes place (8). All patients had had negative or equivocal noninvasive imaging studies before referral to the National Institutes of Health, and many had had nondiagnostic arteriography. This bias accounts for the low sensitivities of computed tomography, ultrasonography, and magnetic resonance imaging in our study compared with sensitivities in other published series (9, 10). Many patients were referred to our institution specifically because of our extensive experience in portal venous sampling. The calcium stimulation test, done at the same time as standard pancreatic arteriography, provides the same information without requiring special skills or experience.

Samples from the right hepatic vein always contained diagnostic elevations of insulin in the presence of tumors in the body and tail of the pancreas. In fact, maximum insulin levels in the right hepatic vein were higher in 80% and ratios of insulin levels in the right hepatic vein to those in the peripheral vein were higher in 90% of patients with tumors of the body and tail of the pancreas. No preferential streaming of the splenic vein effluent into the left branch of the portal vein was evident. A few observations show that it is not necessary to obtain samples from the left hepatic vein, which is more difficult to catheterize than the right hepatic vein. We recommend that a single catheter be placed in the right hepatic vein for arterial stimulation studies in patients with insulin-producing islet cell tumors.

An intravenous calcium stimulation test has been proposed for use in the diagnosis of insulinoma (11–15), although varying sensitivities have been reported (16–19). Brunt and colleagues (11) showed that calcium is a more effective secretagogue in patients with insulinomas when it is administered as a rapid intravenous bolus (2 mg Ca^{++}/kg over 1 minute) than when it is given as a prolonged infusion (12 mg Ca^{++}/kg over 3 hours). We used one tenth of the "rapid intravenous bolus" dose (0.2 mg Ca^{++}/kg) injected over 3 to 5 seconds directly into the artery perfusing the islet cell tumor, thus providing a much more intense calcium exposure over a short period of time. One of our concerns was that calcium stimulation might cause a massive release of insulin and severe, even life-threatening, hypoglycemia. However, insulin levels in the hepatic vein usually peaked in the samples taken 60 seconds after calcium stimulation and were often returning toward baseline by 120 seconds after stimulation, suggesting that the release of insulin is confined to the short interval during which beta cells are exposed to high serum calcium levels.

This failure of intravenous calcium infusion to stimulate the release of insulin from normal β cells suggests that intra-arterial calcium infusion may provide clues to the diagnosis of factitious hypoglycemia caused by sulfonylurea abuse. One patient not included in this series did not show any insulin gradients in hepatic vein samples after intra-arterial calcium stimulation and was later found to have factitious hypoglycemia caused by sulfonylurea ingestion. Portal venous sampling does not provide specificity; elevations of insulin levels several times above baseline can be found in samples from many sites in the splenic and portal veins of patients without insulinomas (Jensen R and Norton J. Unpublished data).

One patient in this series was receiving diazoxide when the calcium stimulation study was done and showed a prompt elevation of insulin levels after injection of calcium into the gastroduodenal artery. However, we generally prefer that patients not be receiving diazoxide at the time of calcium stimulation. In addition, patients who have substantial cardiac disease or who are receiving cardiac glycoside therapy should be closely monitored during calcium infusion studies. However, the total amounts of calcium administered are insignificant (5.25 mEq in a patient weighing 70 kg over about 30 minutes). None of our first five patients showed any change in serum calcium levels during the study.

Calcium stimulation with venous sampling divides the pancreas into two components, one to the right and one to the left of the superior mesenteric artery. This is similar to the regionalization provided by portal venous sampling. Such a partition has surgical relevance because adenomas to the left of the superior mesenteric artery can be treated by enucleation or by distal pancreatectomy, whereas tumors to the right of the superior mesenteric artery must be localized for enucleation. In these latter tumors, the availability of practitioners experienced in intraoperative ultrasonography is critical because adenomas smaller than 15 mm may be difficult to palpate.

Because of the sensitivity of intraoperative ultrasonography (100% in our study and more than 80% in most reported series [1, 8, 10, 20]), one may ask whether any localization studies are necessary before surgery. At the National Institutes of Health, patients with insulinomas have noninvasive imaging (ultrasonography, computed tomography, magnetic resonance imaging, and octreotide scanning), and, if the results of two or more of these studies are positive at the same site, pancreatic arteriography with calcium stimulation is not done. However, if results of the noninvasive examinations are negative or equivocal, the insulinoma tends to be small and intraoperative localization may be difficult, particularly in hospitals with limited experience in intraoperative ultrasonography. Under such circumstances, we consider intra-arterial stimulation with calcium gluconate—a test that

Downloaded From: http://annals.org/ by a Penn State University Hershey User on 06/26/2015

can be competently done at most community hospitals—to be indicated because of its simplicity and high sensitivity.

Our results indicate that calcium stimulation with hepatic venous sampling is as sensitive as and less invasive than portal venous sampling and, thus, should replace portal venous sampling as a technique to localize occult insulinomas. If positive results on a calcium stimulation test indicate an insulinoma of the pancreatic body or tail that cannot be identified intraoperatively, our data suggest that a blind distal pancreatectomy in that setting would be justified.

Addendum

Since this paper was submitted, we studied three more patients with surgically proven insulinomas. In all three patients, the insulinomas were localized correctly (sensitivity for the entire group of 28 patients, 89% [25/28]; CI, 71% to 98%).

Acknowledgments: The authors thank Carla Hendricks, who did the insulin radioimmunoassays and assisted with analysis of the data; Dr. Samuel Wells of Washington University, St. Louis, Missouri, who provided helpful discussion during our selection of the most appropriate secretagogue; and Dr. Charles Lucas of Wayne State University, Detroit, Michigan, who provided surgical and histologic data for one patient.

Requests for Reprints: John L. Doppman, MD, Diagnostic Radiology Department, Clinical Center, National Institutes of Health, Building 10, Room 1C660, 10 Center Drive, MSC 1182, Bethesda, MD 20892-1182.

Current Author Addresses: Drs. Fraker and Alexander: Surgery Branch, National Cancer Institute, National Institutes of Health, Building 10, Room 2B07, 10 Center Drive, MSC 1502, Bethesda, MD 20892-1502.
Dr. Norton: Department of Surgery, Washington University, 510 Kingshighway, St. Louis, MO 63110.
Dr. Miller: Department of Radiology, National Naval Medical Center, 8901 Wisconsin Avenue, Bethesda, Maryland 20889-5000.
Dr. Collier: Clinical Immunology Branch, Division of Allergy Immunology and Transplantation, National Institute of Allergy and Infectious Diseases, 6003 Executive Boulevard, Solar Building 4A20, Bethesda, MD 20892-7640.
Dr. Skarulis: Division of Intramural Research, National Institute of Diabetes and Digestive and Kidney Diseases, National Institutes of Health, Building 10, Room 8S235, 10 Center Drive, MSC 1770, Bethesda, MD 20892-1770.
Dr. Gorden: National Institute of Diabetes and Digestive and Kidney Diseases, National Institutes of Health, Building 31, Room 9A52, Bethesda, MD 20892-2560.

References

1. **Doherty GM, Doppman JL, Shawker TH, Miller DL, Eastman RC, Gorden P, et al.** Results of a prospective strategy to diagnose, localize, and resect insulinomas. Surgery. 1991;110:989-97.
2. **Miller DL, Doppman JL, Metz DC, Maton PN, Norton JA, Jensen RT.** Zollinger-Ellison syndrome: technique, results, and complications of portal venous sampling. Radiology. 1992;182:235-41.
3. **Doppman JL, Miller DL, Chang R, Shawker TH, Gorden P, Norton JA.** Insulinomas: localization with selective intraarterial injection of calcium. Radiology. 1991;178:237-41.
4. **Doppman JL, Miller DL, Chang R, Gorden P, Eastman RC, Norton JA.** Intraarterial calcium stimulation test for detection of insulinomas. World J Surg. 1993;17:439-43.
5. **Doppman JL, Miller DL, Chang R, Maton PN, London JF, Gardner JD, et al.** Gastrinomas: localization by means of selective intraarterial injection of secretin. Radiology. 1990;174:25-9.
6. **Thom AK, Norton JA, Doppman JL, Miller DL, Chang R, Jensen RT.** Prospective study of the use of intraarterial secretin injection and portal venous sampling to localize duodenal gastrinomas. Surgery. 1992;112:1002-9.
7. **Imamura M, Takahashi K, Adachi H, Minematsu S, Shimada Y, Naito M, et al.** Usefulness of selective arterial secretin injection test for localization of gastrinomas in the Zollinger-Ellison syndrome. Ann Surg. 1987;205:230-9.
8. **Norton JA, Shawker TH, Doppman JL, Miller DL, Fraker DL, Cromack DT, et al.** Localization and surgical treatment of occult insulinomas. Ann Surg. 1990;212:615-20.
9. **King CM, Reznek RH, Dacie JE, Wass JA.** Imaging islet cell tumours. Clin Radiol. 1994;49:295-303.
10. **Galiber AK, Reading CC, Charboneau JW, Sheedy PF 2d, James EM, Gorman B, et al.** Localization of pancreatic insulinoma: comparison of pre- and intraoperative US with CT and angiography. Radiology. 1988;166:405-8.
11. **Brunt LM, Veldhuis JD, Dilley WG, Farndon JR, Santen RJ, Leight GS, et al.** Stimulation of insulin secretion by rapid intravenous calcium infusion in patients with beta-cell neoplasms of the pancreas. J Clin Endocrinol Metab. 1986;62:210-6.
12. **Kaplan EL, Rubenstein AH, Evans R, Lee CH, Klementschitsch P.** Calcium infusion: a new provocative test for insulinomas. Ann Surg. 1979;190:501-7.
13. **Gaeke RF, Kaplan EL, Rubinstein A, Starr J, Burke G.** Insulin and proinsulin release during calcium infusion in a patient with islet-cell tumor. Metabolism. 1975;24:1029-34.
14. **Harrison TS, Santen RJ, Maruca J.** Calcium stimulation test in evaluation of insulin secreting pancreatic islet cell tumors: case reports. Mil Med. 1981;146:103-5.
15. **Roy BK, Abuid J, Wendorff H, Nitiyanant W, DeRobertis FR, Field JB.** Insulin release in response to calcium in a diagnosis of insulinoma. Metabolism. 1979;28:246-52.
16. **De Palo C, Sicolo N, Vettor R, Federspil G.** Lack of effect of calcium infusion on blood glucose and plasma insulin levels in patients with insulinoma. J Clin Endocrinol Metab. 1981;52:804-6.
17. **Kakita K, Horino M, Tenku A, Matsumura S, Matsuki M, Nishida S.** Absence of insulin release in response to calcium in a patient with benign insulinoma. Horm Metab Res. 1981;13:237-8.
18. **Miller JL, Klaff LJ, Abrahamson MJ, Marine N.** Failure of calcium infusion as a provocative test for insulin [Letter]. N Engl J Med. 1981;304:1430.
19. **Pointel JP, Villaume C, Gay G, Drouin P, Debry G.** Absence of effect of calcium perfusion on blood sugar and plasma insulin in a patient with a benign insulinoma. Horm Metab Res. 1978;10:572-3.
20. **Grant CS, van Heerden J, Charboneau JW, James EM, and Reading CC.** Insulinoma. The value of intraoperative ultrasonography. Arch Surg. 1988;123:843-8.

Downloaded From: http://annals.org/ by a Penn State University Hershey User on 06/26/2015
