# Supplementary material for: Diagnostic value of ASVS for insulinoma localization: A systematic review and meta-analysis
Source: PLoS One. 2019 Nov 19;14(11):e0224928. doi: 10.1371/journal.pone.0224928 (PMC6863549; doi:10.1371/journal.pone.0224928)
Supplement: S2 File — (ZIP) [file pone.0224928.s002.zip › included studies/ModifiedIntra-arterialCalciumS.pdf]

Abdom Imaging 23:322–331 (1998)

Abdominal Imaging
© Springer-Verlag New York Inc. 1998

Modified intra-arterial calcium stimulation with venous sampling test for preoperative localization of insulinomas

L. Defreyne,[1] K. König,[2] M. M. Lerch,[3] U. J. Hesse,[4] R. Rottiers,[5] G. Feifel,[6] B. de Hemptinne,[4] B. Kramann,[2] M. Kunnen[1]

[1]Radiologie en Medische Beeldvorming, Vasculaire en Interventionele Radiologie, Universitair Ziekenhuis Gent, De Pintelaan 185, B-9000 Gent, Belgium
[2]Radiologische Klinik, Abteilung für Radiodiagnostik, Universitätskliniken des Saarlandes, Oscar Orth Strasse 1, D-66421 Homburg, Germany
[3]Medizinische Klinik II, Universitätskliniken des Saarlandes, Oscar Orth Strasse 1, D-66421 Homburg, Germany
[4]Heelkundige Kliniek, Universitair Ziekenhuis Gent, De Pintelaan 185, B-9000 Gent, Belgium
[5]Dienst voor Inwendige Ziekten, Endocrinologie, Universitair Ziekenhuis Gent, De Pintelaan 185, B-9000 Gent, Belgium
[6]Klinik für Allgemein-, Abdominal und Gefäßchirurgie, Universitätskliniken des Saarlandes, Oscar Orth Strasse 1, D-66421 Homburg, Germany

Received: 21 January 21 1997/Accepted: 19 March 1997

Abstract

Background: To determine the accuracy and safety of a modified intra-arterial calcium stimulation with the venous sampling test (ASVS) for preoperative localization of insulinomas. Modification included stimulation with a fixed low dose of calcium gluconate, additional stimulation in the distal splenic artery, and no insulin sampling in the left hepatic vein.

Methods: In 10 patients showing biochemical evidence of organic hyperinsulinemia, 0.45 mmol of Ca^{2+} was injected into the gastroduodenal, superior mesenteric, proper hepatic, proximal, and distal splenic arteries during angiography. Insulin levels were measured in samples taken from the right hepatic vein before and 30, 60, 90, 120, 180, and 300 s after Ca^{2+} injection.

Results: Insulin gradients with an increase of more than fourfold indicated direct tumor supply, two- to fourfold correlated with collateral supply, and less than twofold correlated with normal tissue vascularization. ASVS localized all the adenomas of the pancreatic head (n = 3) and body (n = 2) and two of four adenomas of the tail correctly, as confirmed by surgery. Two adenomas of the proximal pancreatic tail were erroneously localized to the body segment, but the fault was rectified by angiography. In one patient with a negative ASVS and without exploration, the diagnosis of an insulinoma was revised.

Conclusion: ASVS with a fixed low dose of calcium gluconate is a highly accurate and safe method for preoperative localization of insulinomas. Sampling in the left hepatic vein can be routinely omitted. Additional stimulation in the distal splenic artery seems helpful in surgical decision making, but additional experience is needed.

Key words: Pancreas angiography—Pancreas neoplasms—Venous blood sampling—Hormones.

Correspondence to: L. Defreyne

Accurate diagnosis of organic hyperinsulinemia, which provokes intermittent hypoglycemia, is possible with functional endocrine tests, particularly with the supervised 72-h fast, which measures serum insulin, glucose, and C-peptide concentrations [1]. In about 90% of cases, organic hyperinsulinemia is caused by a benign, solitary, and usually small β-cell adenoma; in the remaining 10% of cases, multiple adenomas, as part of a multiple endocrine neoplasia (MEN) or a malignant tumor, are found [2–5].

Patients with a benign insulinoma are potential candidates for curative surgery. However, laparatomy outcome is unpredictable because doubt exists as to the number, localization, and histopathology of the insulinoma(s). For this reason, a wide range of imaging modalities has been deployed to visualize these tumors preoperatively. Reports on the use of ultrasonography

(US), computed tomography (CT), and conventional angiography have been published, but these reports present variable and often poor results [6–10]. These inconsistent results have prompted the development of a more sensitive localization method, such as percutaneous portal vein insulin sampling (PVS) [11]. However, this technique requires special skills and experience, which are difficult to achieve because insulinomas are rare, with an incidence of only one to four per 1 million person-years [12–15]. Reports on dynamic CT and magnetic resonance imaging (MRI) have seemed promising [16–18].

Since the study of Dagett et al. in 1981 [19], surgeons have discussed whether any imaging technique before first operation of an insulinoma is necessary [20–22]. Despite this suggestion, many surgical teams do not rely exclusively on bimanual palpation of the pancreas combined with intraoperative ultrasound (IOUS), but they do attribute their high operative success rates to meticulous preoperative localization [23–24]. Moreover, recent single and multicenter studies conducted to evaluate new imaging modalities, such as endoscopic ultrasonography (EUS) and intra-arterial calcium stimulation with hepatic venous insulin sampling (ASVS), point to a continuous multidisciplinary interest in preoperative localization [25–26].

ASVS is a functional extension of pancreatic angiography based on the stimulation of endocrine tumors by secretagogues. In 1975, Gaeke et al. first described an acute rise of serum insulin during intravenous calcium infusion in a patient with an insulinoma [27]. A few years later, Kaplan et al. investigated venous calcium infusions in normal individuals and in patients with organic hyperinsulinism [28]. Finally, Brunt et al. refined the intravenous calcium provocation test by administrating Ca^{2+} as a rapid bolus of 2 mg/kg/min [29].

Only recently has ASVS been successfully applied in localizing gastrinomas [30]. By substituting secretin with calcium gluconate and sampling insulin instead of gastrin in the hepatic veins, Doppman et al. demonstrated the efficacy of ASVS to localize insulinomas in the head or the body–tail region of the pancreas [31–32]. However, the localization precision and the relevance of ASVS have been criticized [33–34]. To improve the localization accuracy, a modification of the original ASVS has been suggested [35], including proximal and distal splenic artery stimulation.

To simplify the procedure and to increase its safety, we also omitted left hepatic vein sampling and stimulated intraarterially with a fixed, reduced dose of calcium gluconate. Results of this modifications of ASVS in localizing insulinomas are presented in this study.

Materials and methods

Ten consecutive patients, referred for suspected organic hyperinsulinemia, were included in this prospective study. Age, sex, and history of the patients are summarized in Table 1. Inclusion criteria required coexisting hypoglycemic symptoms with low fasting serum glucose levels ($<$50 mg/dL) and biochemical evidence of organic hyperinsulinemia confirmed by a fasting test (serum insulin:glucose ratio $\geq$ 0.3, with high C peptid). MEN or other associated endocrine diseases were confirmed with specific hormone testing in two patients (patient 5: hyperthyroidism; patient 6: hyperparathyroidism as part of MEN I).

Fasting tests were performed in nine of 10 patients and were conclusive in seven of nine cases. In the first case, the fasting test was aborted because the patient lost consciousness. The blood samples taken at that time were lost, but earlier samples of glucose, insulin, and C peptide suggested organic hyperinsulinemia. In case 3, several fasting blood glucose levels below 40 mg/dL were measured, but the fasting test result (insulin:glucose ratio = 0.28) was inconclusive. Patient 6 had previously undergone left pancreatectomy for multiple β-cell adenomas as part of MEN I syndrome (primary hyperparathyroidism). Surgical treatment resulted in only short-term improvement. For more than 15 years, conservative therapy with acetazolamide and antidepressive drugs controlled the neuroadrenergic complaints of this patient, but he had deteriorated to a deep depressive state. US showed a large tumor of the pancreatic head, considered to be a β-cell adenoma. Because the patient survived by depending on permanent glucose infusions, we refrained from a fasting test.

Radiological imaging modalities employed for localization of the pancreatic adenoma are summarized in Table 2. Referring hospitals generally performed noninvasive imaging with US, CT, and MRI in two cases. The detection rate was variable, reflecting the wide range of success of preoperative localization by US and CT in the literature. In the few cases assessed by new imaging modalities such as MRI and EUS, success rates seem to be higher. For cost effectiveness, noninvasive imaging was not completed at our hospital.

In all but one case, pancreatic angiography was performed as part of the calcium stimulation test at the university hospital. PVS was not routinely planned but was considered when results of intra-arterial calcium provocation were inconclusive. This diagnostic problem arose only in patient 3, but after consulting the referring colleagues, PVS was postponed.

After obtaining the patients' informed consent, ASVS was basically performed according the protocol proposed by Doppmann et al. [26]. To sample hepatic venous blood, a 5 or 7 French (F) cobra catheter with two side holes (Cook, Denmark) was inserted transfemorally into the right hepatic vein. The contralateral femoral artery was then punctured, and a 5 F precurved (cobra or sidewinder) catheter (Cook, Denmark) was advanced to the celiac trunk and the mesenteric artery. Pancreatic and hepatic angiography were achieved by selective catheterization of the arteries supplying the body and tail (proximal splenic artery), the head (gastroduodenal and superior mesenteric artery), and the liver (proper hepatic artery) whenever possible. Angiographic studies were carried out by conventional cut film technique in the first four cases and with digital subtraction angiography (DSA) in the other cases. Immediately after each angiographic study, calcium gluconate was injected intra-arterially to provoke secretion of insulin from the β-cell adenoma. From this point, we modified the original approach in three ways.

First, we did not sample in the left hepatic vein. Catheterization of the left hepatic vein may be difficult and, as a consequence, complicate the procedure. Selective distribution of splenic and mesenteric venous blood to the left portal vein and the right portal vein, respectively, may mask an adenoma in the body–tail region, but such portal flow dynamics have never been proved.

Second, for technical reasons, surgeons asked us to exclude a tumor in the pancreatic tail and the splenic hilus. Therefore, additional catheterization of the distal splenic artery, beyond the origin of the arteria pancreatica magna, to stimulate the tail region was done with a superselective 3 F straight catheter (Cook, Danemark or Target Therapeutics, USA).

Third, we chose a fixed dose of 0.275 g of calcium gluconate equating 0.448 mmol Ca^{2+} (2 mL of Calcium Sandoz 10%, Labora-

Table 1. Patient data and fasting test results

Patient number/ age (years)/sex	History (months)	Insulin/glucose ratio[a]	Interruption of fasting after *x* h	C peptide (pmol/mL)
1/73/F	4	0.61	2	1.15
2/59/F	36	0.45	29	0.8
3/22/F	12	0.28	52	0.32
4/39/F	2	1.30	32	1.15
5/61/F	2	1.08	28	0.66
6/63/M	192	*	*	*
7/56/F	6	0.95	15	1.26
8/36/M	10	1.20	8	1.1
9/45/F	3	0.76	8	0.73
10/57/F	60	0.30	14	0.36

[a] Insulin (μU/mL):glucose (mg/dL) ratio ≥ 0.3 = pathologic
* No data available

Table 2. Results of preoperative medical imaging[a]

Patient	US	CT	MRI	EUS	Angiography
1	0	++	nd	nd	++
2	0	0	0	nd	+
3	0	0	nd	nd	0
4	0	0	nd	++	+++
5	0	++	+++	nd	+++
6	+++	+++	nd	nd	+++
7	0	0	nd	+++	+++
8	+++	+++	nd	nd	+++
9	0	0	nd	nd	+++
10	0	++	0	nd	+++

[a] nd = not done; 0 = no tumor seen; + = tumor recognized after ASVS; ++ = mass detected but not clearly defined as adenoma; +++ = clear-cut diagnosis of adenoma

toires Sandoz, France) for injection into each artery and abandoned the original formula that takes into account the patient's body weight (normal weighted individuals are given 0.0125 mmol of Ca^{2+}/kg body weight, and obese patients are given 0.005 mmol of Ca^{2+}/kg body weight). We intended to contest the hypothesis that the amount of secretagogue needed for stimulation of the adenoma is based on the patient's body weight [26]. Moreover, if we could find an appropriate fixed and low dose of calcium, calculation mistakes and hypoglycemic side effects might be anticipated, thus making the procedure easier and safer.

The calcium gluconate was then diluted with saline to a 5-mL solution and injected as a bolus. Before and 30, 60, 90, 120, 180, and 300 s after each ASVS, 5 mL of hepatic vein blood were sampled. Each sample was labeled according to time interval and artery of provocation and stored at 4°C. Insulin concentrations were measured with a radioimmunoassay test.

All tests were carried out under local anesthesia and with continuous electrocardiographic and blood pressure monitoring. To avoid iatrogenic hypoglycemia, peripheral venous blood sugar samples were taken after each calcium injection in the first five patients. Blood sugar levels were checked only when the patient's physical reaction indicated hypoglycemia.

Treatment was not part of the protocol. Seven patients underwent surgery at the university hospital. Two patients preferred surgery at their home hospitals, which were major referring hospitals associated with a university medical center. Although six different surgeons were involved, operation reports revealed the use of similar techniques. The pancreas was exposed through the bursa omentalis by a median upper abdominal incision, and dissection was guided by the results of ASVS. Enucleation of the adenoma was preferred, and a left pancreatectomy with or without splenectomy was the alternative. Surgeons routinely applied bimanual palpation and IOUS. Once the adenoma was identified, they reduced the dissection of the pancreas to a minimum.

Results

Table 3 summarizes the visceral vessels catheterized for angiography and calcium provocation. Six arterial catheterizations fulfilled the conditions of the protocol. In one case, the investigator had to advance the microcatheter into the artery supplying the adenoma, which was the dorsal pancreatic artery branching from the proximal common hepatic artery (Fig. 1). This decision was guided by tumor blush on the preceding angiogram. Acute insulin release confirmed the presumptive diagnosis of tumor presence and supply. In two other cases, deviation from the protocol was necessary because stable selective catheterization of the common hepatic artery and gastroduodenal artery had failed. The catheter was positioned in the artery most proximal to it (celiac trunk; common hepatic artery). In patient 6, the splenic

Table 3. Results of ASVS listed per patient and per artery[a]

	Patients									
Arteries	1	2	3	4	5	6	7	8	9	10
Superior mesenteric artery	0	0	0	0	0	0	0	0	0	0
Gastroduodenal artery	—	0	0	0	—	3.21/2.00[b]	0	0	2.66	7.23
Distal splenic artery	10.75	0	0	0	0[c]	—	0	4.01	0	0
Proximal splenic artery	9.37	15.39	0	5.88	4.65	—	12.21	6.17	0	0
Proper hepatic artery	—	0	0	0	0	0	0	0	0	0
Other arteries										
Common hepatic artery	—	—	—	—	0	—	—	—	0[d]	7.90[e]
Celiac trunk	6.99	—	—	—	—	—	—	—	—	—
Dorsal pancreatic artery	—	—	—	—	—	0	—	—	15.96	—

[a] Insulin gradients were measured as the ratios of venous hepatic insulin concentration at 30 or 60 s after ASVS to the insulin concentration before ASVS; 0 = gradient <2.0; — = not catheterized

[b] Two arteries arising from the gastroduodenal artery: gradients after 120 s were 4.4 and 3.1, respectively

[c] Insulin gradient was 1.2 after 60 s and 1.7 after 90 s (see also Fig. 4)

[d] Tip of catheter distal to the origin of the dorsal pancreatic artery; stable proximal catheterization failed

[e] Tip of catheter proximal to origin of the dorsal pancreatic artery

artery had been ligated during a previous left pancreatectomy; therefore, the catheter was positioned in the dorsal pancreatic artery supplying the remnant of the pancreatic body.

In all but two cases, ASVS provoked at least one acute release of insulin within 60 s after calcium injection into the artery supplying the tumor (Fig. 2). The insulin level in the right hepatic vein increased to fourfold of the baseline level. In four cases, even a 10-fold elevation was noted. Mean positive insulin gradient was 7.4 (range = 4.4–15.9), and the average insulin peak within 60 s was 366 μU/mL (range = 277–419). Surgery confirmed adenoma in all cases. In case 6, a malignant insulinoma, the insulin curve showed a fourfold increase but only after 120 s. In the only case with a negative ASVS, follow-up of the patient and the repeated fasting test were negative.

Two- to fourfold increases of insulin level were observed after ASVS in arteries supplying pancreatic tissue neighboring the tumor site in two patients. In patient 9, an insulin gradient of 2.1 was measured after 120 s in the distal splenic artery.

Gradients near to twofold (between 1.9- and 2.0-fold) were observed after 120 s in the patient without insulinoma. Two additional patients with a proven insulinoma showed a similar low insulin increase after injection in an artery close to the tumor segment.

Size, location, and number of adenomas found at surgery are listed in Table 4. Other than patient 6, who suffered from two large head insulinomas, seven of the eight other insulinomas were small (mean = 10.7 mm, range = 10–19 mm), and one was medium sized (33 mm). All of these eight insulinomas were solitary and were located in the tail (n = 4), in the body (n = 2), and in the head (n = 2) of the pancreas. Agreement on location between ASVS and surgery existed in seven of nine patients. In two cases with a tail insulinoma, the correlation was not complete because ASVS in the distal splenic artery was negative (Fig. 3). Sensitivity and specificity of ASVS for head insulinomas and for insulinomas in the body–tail region (''left'' of the mesenteric artery) were 100%. If body and tail of the pancreas are considered separately, sensitivity and specificity, respectively, of ASVS were 100% and 71% for body insulinoma and 50% and 100% for tail insulinomas. Positive and negative predictive values of ASVS were 100% for body insulinomas, but 50% and 71%, respectively, for tail insulinomas.

Five patients were treated by enucleation of the insulinoma, two by tail resection and splenectomy, one by tail resection, and another by residual pancreatectomy. In six of eight cases, the surgeons easily discovered the tumor at the predicted location. One insulinoma was detected only after laborious bimanual palpation and IOUS of the whole pancreas and intensive reinspection of the predicted area. One insulinoma was missed intraoperatively. Relying on the ASVS, the surgeons undertook a blind tail resection and splenectomy, which proved to be correct. In this case, transsection of the specimen revealed a 15-mm adenoma in the tip of the tail, hidden in the hilus of the spleen. In patient 6, the pancreatic head was replaced by a huge tumor and a smaller satellite lesion. Resection was radical and included both tumors, the head, and the remnant of the pancreatic body. Aware of the negative ASVS for the body region, the surgeons initially attempted to save the pancreatic body, but this turned out to be technically impossible. Histopathology confirmed two malignant insulinomas with a tumor-free pancreatic body. No patient had locoregional or hepatic metastatic disease on peroperative palpation or IOUS.

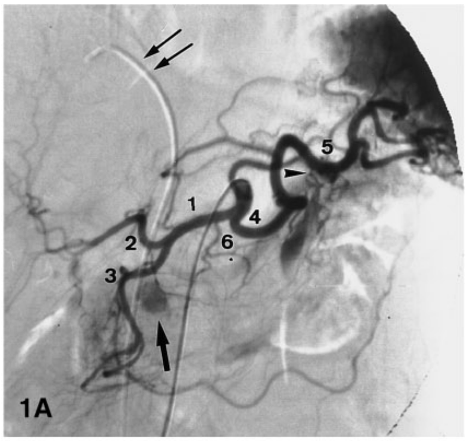

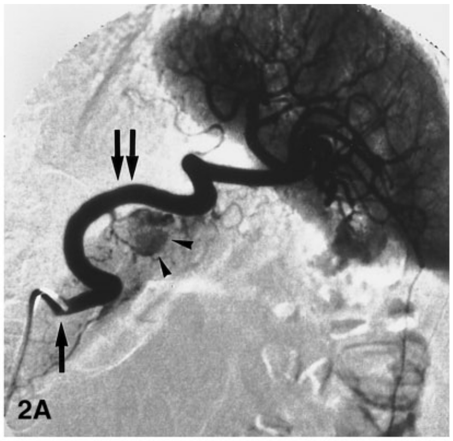

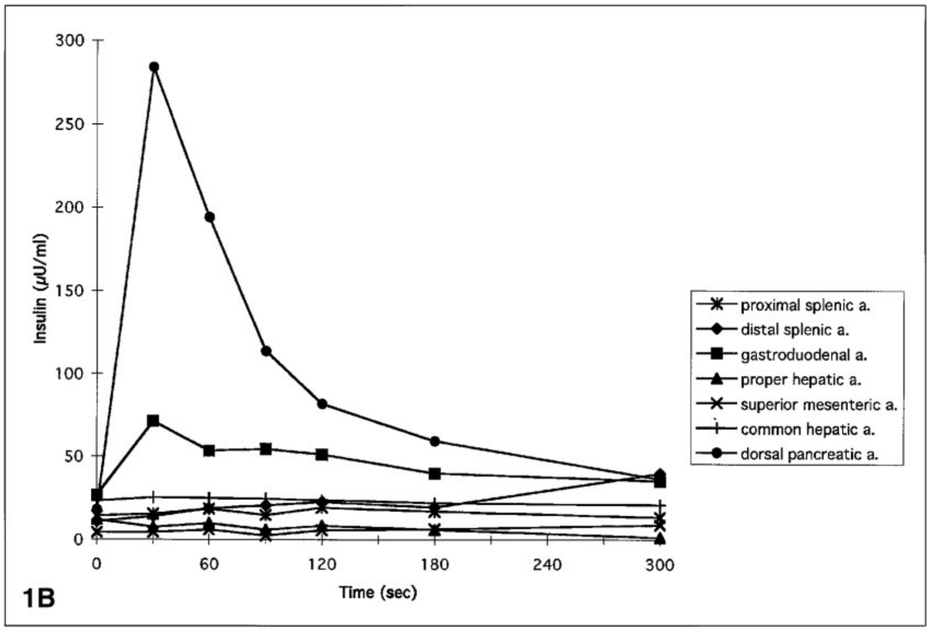

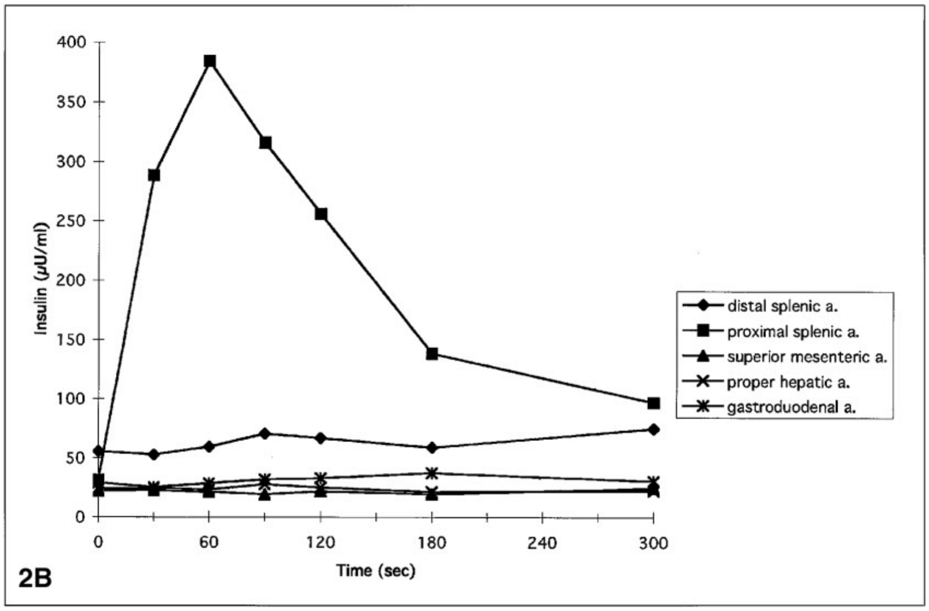

Fig. 1. Angiography and insulin gradients after ASVS in case 9 (pancreatic head insulinoma). **A** Celiac tributaries with stimulation sites: common hepatic artery (1), proper hepatic artery (2), gastroduodenal artery (3), proximal (4) and distal (5) splenic arteries, and dorsal pancreatic artery (6) supplying the adenoma (*arrow*). Arteria pancreatica magna (*arrowhead*) and sampling catheter in the right hepatic vein (*double arrow*) are visible. **B** A 16-fold rise after stimulation in the dorsal pancreatic artery branching from the proximal common hepatic artery and directly supplying the adenoma. Stable proximal catheterization of common hepatic artery was not possible. A 2.7-fold rise after ASVS in the gastroduodenal artery was noted from the collateral or minor supply. Note also 2.1-fold rise 120 s after ASVS in the distal splenic artery.

Fig. 2. Splenic angiography and ASVS in case 7 (pancreatic body insulinoma). **A** Stimulation proximally (catheter tip: *arrow*) and distally beyond the origin of the arteria pancreatica magna (*double arrow*), supplying a hypervascular tumor in the body region (*arrowheads*). **B** Greater than 12-fold rise of insulin after stimulation in the proximal splenic artery indicates a body insulinoma, which was confirmed by surgery.

Table 4. Agreement between site of insulinoma predicted by ASVS and that detected during surgery

Patient	Number of tumors	Size (mm)[a]	Location	Therapy	Detectability	Correlation with ASVS
1	1	15	Tail	Tail resection and splenectomy	Not found	Complete
2	1	11	Body	Enucleation	Easy	Complete
3[b]						
4	1	15	Tail	Enucleation	Very difficult	Good[c]
5	1	19	Tail	Tail resection and splenectomy	Easy	Good[c]
6	2	100 and 30	Head	Pancreatectomy	Easy	Complete
7	1	10	Body	Enucleation	Easy	Complete
8	1	33	Tail	Tail resection	Easy	Complete
9	1	11	Head	Enucleation	Easy	Complete
10	1	13	Head	Enucleation	Easy	Complete

[a] Largest diameter of the tumor
[b] No surgical exploration
[c] Body adenoma was predicted, but a proximal tail adenoma was found (ASVS result corrected by angiography)

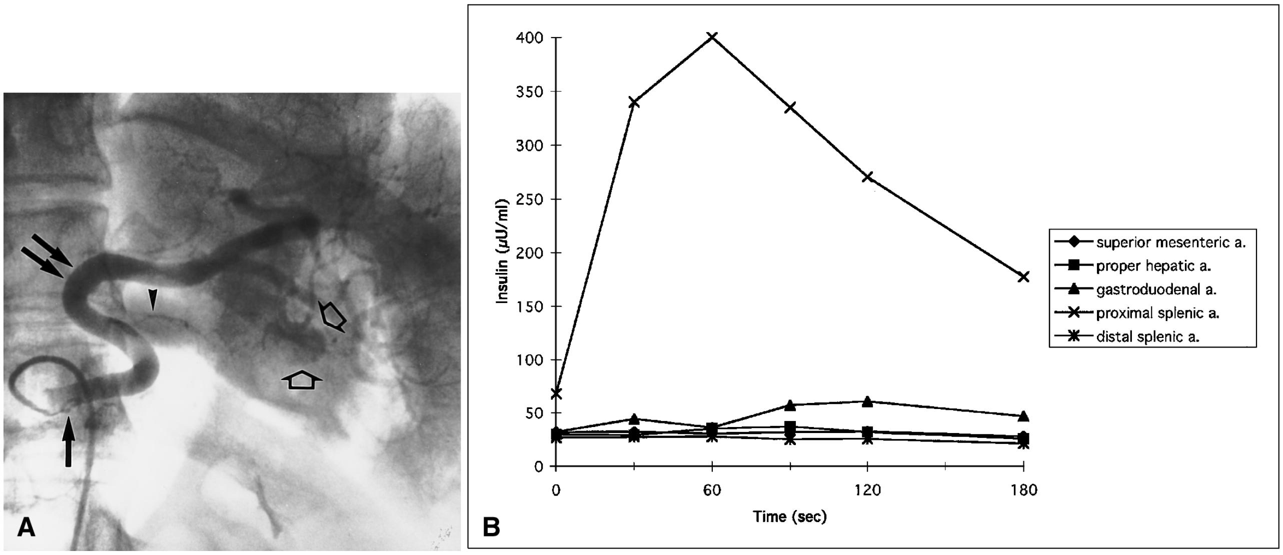

Fig. 3. Splenic angiography and ASVS in case 4 (pancreatic tail adenoma). **A** Sites of calcium injection: proximal (*arrow*) and distal (*double arrow*) splenic arteries. Arteria pancreatica magna (*arrowhead*). Blood supply of the insulinoma (*open arrows*) in the tail region is not very traceable. **B** A sixfold rise of insulin level was noted after injecting calcium gluconate into the proximal splenic artery but no rise after distal stimulation. ASVS erroneously localized this tail adenoma in the body, most probably because of the dominant supply of the tail and tumor by the transversally running branch of the arteria pancreatica magna.

Postoperative morbidity included subphrenic fluid collection in one patient, pancreatic fluid leakage with cutaneous fistula in another, and self-limiting pancreatitis in a third. All complications were treated conservatively. The mean duration of hospitalization was 26 days.

Mean follow-up of all patients was 32 months (range = 5–57). Surgery cured eight of eight patients with benign insulinoma, without any long-term complaints or complications. The patient who underwent total pancreatectomy for malignant insulinomas became an insulin-dependent diabetic but had no more hypoglycaemic attacks. Restaging for locoregional or hepatic metastases remained negative.

Discussion

Preoperative localization studies for insulinomas are time and money consuming if their localization accuracy does not allow rational and safe surgical enucleation or resection [36]. Sensitivity and specificity must compete with the most accurate localization technique of all, which is IOUS combined with bimanual palpa-

tion, failing only in fewer than 5% of cases [19–21]. ASVS, as introduced by Doppman et al. in 1991, challenged this high standard, but as Axelrod recently countered [34], the test has critical limitations. ASVS does not localize the adenoma to an exact area in the pancreas, but to the ''right or left side of the mesenteric artery,'' which would be a questionable surgical advantage [34]. Moreover, it remains controversial if a precise preoperative localization improves the outcome [34]. When starting ASVS in 1991, we modified the original protocol to meet the surgeons' requirements. We will discuss the characteristics of insulin release during ASVS and the accuracy of localization, resulting from a combined analysis of ASVS and angiography. Furthermore, the short- and long-term surgical outcome will be compared with the ASVS results.

Characteristics of insulin release during ASVS

In general, ASVS is considered as positive if a greater than twofold insulin rise in the right hepatic vein is measured after provocation in the artery supplying the adenoma [32, 37]. However, in a recent publication on six patients, Hayashi et al. reported greater than twofold elevations of insulin after ASVS in nontumor supplying arteries [38]. Consequently, they recommended that, if the insulin gradient is greater than only twofold, the maximum insulin concentration should exceed 150 μU/mL. Interestingly, the rapid intravenous calcium provocation test may also provoke a greater than twofold (and <fourfold) serum insulin increase in normal individuals [29].

From our study, we distinguished three levels of insulin gradient after ASVS: less than twofold, between two- and fourfold, and greater than fourfold (Table 3). If the insulin gradient in the right hepatic vein is at least fourfold within 60 s after provocation in the artery supplying the adenoma, ASVS is definitively positive. Two- to fourfold rises were rarely observed after ASVS of normal pancreatic tissue and of tumor-neighboring segments of the pancreas, probably as a result of collateral or minor vascular supply. Quantitative insulin data in our study endorsed the threshold insulin peak recommended by Hayashi et al. However, by considering a fourfold gradient as diagnostic, we do not need to comply with it.

Insulin responses below or nearly twofold were encountered only after ASVS in the normal subject and after stimulation of normal pancreatic tissue in the others. The curve of insulin release, however, was always much flatter. These low insulin gradients are most likely caused by a physiologic reaction of normal pancreatic islet cells and should not be confused with pathologic secretion by an adenoma.

Why did ASVS in our series produce higher insulin gradients than those found by other investigators [32, 37]? First of all, in the paper by O'Shea et al. [37], although five of the six positive insulin curves showed a greater than fourfold rise in insulin concentration, they endorsed a diagnostic twofold insulin gradient, most probably because of one surgically not explored patient who presented a lower, threefold gradient. In this case, a larger dose of calcium (1 mg/kg) was used, as proposed by Doppman et al. [32]. When O'Shea et al. reduced the dose of calcium to 0.25 mg/kg in subsequent patients because of hypoglycemic complications, rise in insulin concentration was always greater than fourfold. Therefore, if a relationship exists between the dose of injected calcium and the insulin gradients, it seems to be an inversely proportional one. There is much in vitro evidence that calcium-mediated insulin release is related to the voltage-dependent and voltage-independent calcium channels on the cell membrane [39]. The voltage-dependent channels open during membrane depolarization, physiologically mediated by nutrient secretagogues (i.e., glucose) or hormones (i.e., glucagon) [40], whereas the voltage-independent channels seem to permit direct calcium influx. However, the physiology of the voltage-independent calcium channels is still unclear [39], but our experience suggests that it would be interesting to investigate whether these channels are involved in a dose-dependent negative feedback on the voltage-dependent channels. Furthermore, counterregulatory mechanisms involving hormones or peptides such as glucagon or epinephrine may play a role in limiting insulin peaks after ASVS [41]. Finally, insulin concentrations may differ because of deviating measure ranges and variances in the used immunoassays in the different papers. In our series, the use of immunoassays from two different manufacturers did not affect insulin gradients.

In all benign insulinomas in this study, insulin release curves were characterized by a precipitous slope, immediate set off, a peak between 30 and 60 s, and a return to baseline level after 3–5 min. In contrast, in the patient with β-cell carcinoma, the initial increase occurred slowly, with a maximum only after 90–120 s and remaining high for more than 5 min, thus building a plateau phase. Maybe tumor size and neovascularization account for this phenomenon because resistance to arterial perfusion and interstitial diffusion has been proved to be proportional to the tumor volume and degree of necrosis [42]. In huge malignant tumors as in our case, it takes longer for calcium gluconate to reach the cell membrane, and the release of insulin may be more gradual. However, structural difference in calcium sensitivity in MEN I adenomas versus single benign adenomas may be important, in comparison with the opposite plasma insulin response to secretin in benign adenomas and in the multiple β-cell adenoma or hyperplasia syndrome [43].

Although the resected specimen in patient 8 revealed a larger adenoma, with histological arguments for malignancy expressed by vessel invasion, the insulin release curve exactly resembled the benign insulinoma curve. However, further follow-up is necessary to confirm malignancy in this case.

Misleading lower insulin gradients may result from a gradual rise in the prestimulation insulin level. We observed this reaction in two of our patients. In the patient with the malignant tumor, venous insulin concentration after positive ASVS decreased more slowly and interfered with the insulin level prior to the next stimulation. In case 5, with a benign insulinoma, baseline insulin levels in the right hepatic vein rose steadily from 33.4 μU/mL to 120.0 μU/mL before the last calcium injection, although positive tumor reaction only occurred after the fourth injection (Fig. 4). The insulin gradient after the fifth and last stimulation (in the distal splenic artery, supplying the tail adenoma) was less than twofold, but greater than fourfold was expected and indeed confirmed when calculated on the basis of the first prestimulation insulin level (Fig. 4). Small and diluted amounts of recirculated calcium gluconate cannot break this barrier and stimulate the insulinoma. As for the intravenous calcium stimulation test, recirculation of calcium with stimulation of normal β cells is most likely responsible for gradually rising baseline insulin levels. It is not clear why this happened only in this particular case, where a sufficient time of delay was respected between the subsequent calcium injections to avoid accumulation of calcium. Nevertheless, we advise a minimum of a 15-min delay between the provocations to avoid false-negative gradients.

Major complications of intraarterial calcium gluconate injection did not occur. Patients mentioned a warm feeling in the upper abdomen or sweating for less than 1 min as minor side effects. Hypoglycemia did not seem to be a problem with the reduced fixed dose of calcium. Significant rises in serum calcium levels are not expected with such low doses of intra-arterially injected calcium gluconate, which in total correspond to two-thirds of the dose injected intravenously as a bolus over 1 min by Brunt et al. [29]. Of course, one should be aware of electrolyte disturbances and interference with inotrope medication, such as digitalis.

Localization precision

Localization accuracy of ASVS is based on arterial anatomy as depicted by the pancreatic angiography and a

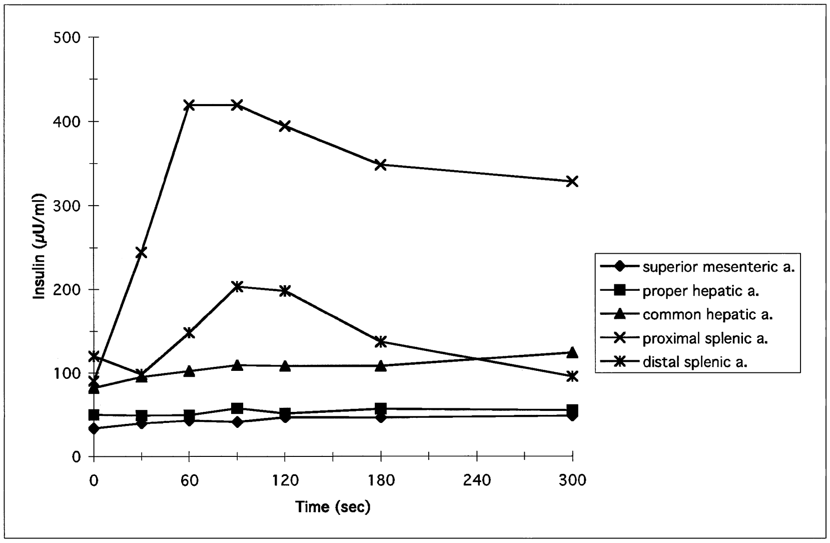

Fig. 4. Insulin gradients after ASVS in case 5 (pancreatic tail insulinoma). There was a gradual rise of prestimulation insulin levels from 33.4 to 120 μU/mL, reducing insulin gradients. Actual insulin gradient within 60 s after ASVS in the distal splenic artery is 1.2-fold, but this gradient based on first prestimulation insulin level: 148/33.4 μU/mL = 4.4. Sequence of ASVS in time order as in legend.

functional test of insulin release as assessed by the method and criteria described in the previous section.

When considering the functional stimulation test independently from the outcome of the angiography, all insulinomas of the pancreatic head and body were detected correctly and localized ''blindly.''

Of four tail adenomas, which we tried to separate from the body tumors by proximal versus distal splenic ASVS, only two were correctly localized. Both other insulinomas were located in the proximal tail, although two body adenomas were predicted by ASVS alone. We believe that this low yield is explained by variations in vascular supply of the distal body and tail region. Anatomically, there is no intrinsic landmark that separates the body from the tail. As the pancreas develops to the left and crosses the anterior superior surface of the left kidney, the gland takes the shape of a tail [44] and receives a major arterial supply from the caudal pancreatic arteries and a minor supply from the left branches of the arteria pancreatica magna, anastomozing with the former artery [45]. With the catheter tip positioned beyond the origin of the arteria pancreatica magna, ASVS will wrongly localize a proximal tail adenoma to the body region if the tumor is supplied by a left branch of this artery. From a surgical point of view, mistaking a proximal tail for a body localization offers no major problems because both segments of the pancreas are well exposed by the division of the gastrocolic ligament and opening of the bursa omentalis. However, preoperative exclusion of an adenoma in the distal tail avoids a difficult mobilization and dissection of the tail and spleen.

Seven of nine (77%) insulinomas were already unequivocally visualized by angiography before ASVS. One tail adenoma in the hilus of the spleen could not be differentiated from an accessory spleen, which is a well-known problem [46–47]. One body adenoma was detected only after subtraction of conventional angiography, after knowledge of the result of ASVS.

Combination of angiography and ASVS resulted in precise localization of all adenomas, not only to the ''right'' or the ''left'' of the mesenteric artery but also to an exact location in the head, body, or tail of the pancreas.

Although the mean size of 15.8 mm of benign insulinomas in our study hardly differs from the 15 mm in Doppman et al.'s series, their low sensitivity of detecting insulinomas with angiography (36%) is surprising [32]. We probably achieved a higher detection rate by using DSA. Although the potential advantage of DSA over conventional angiography have been suggested previously [48], no comparative results have been published until now.

Furthermore, while performing catheterization for ASVS, one should carefully depict all pancreatic arteries and search for variations. Before ASVS, an overview study of the whole celiac trunk and mesenteric artery is mandatory. This need for an overview was well demonstrated in cases 9 (Fig. 1) and 10, in which a head adenoma was supplied by the dorsal pancreatic artery, branching from the common hepatic artery. In this area, with many vascular variations [45], the arteriographic part of the protocol should not be followed blindly but adapted to the specific situation of the individual patient.

The detection of liver metastasis of endocrine pancreatic tumors by ASVS may assist clinical management [49]; however, in case of insulinomas, there is no such experience with ASVS. As a consequence of a simplified sampling procedure restricted to the right hepatic vein [32, 35], metastases in the left liver lobe will be missed. Moreover, because the incidence of liver me-

tastasis of insulinomas is low, we think that the role for ASVS in the proper hepatic artery should be reevaluated.

In two cases, the contribution of ASVS was essential for surgical success. In all other cases, ASVS facilitated the exploration of the pancreas to enucleate the adenoma or to perform a left pancreatic resection. Unlike the results in many recent reports [5, 15, 20, 21, 23], outcome in our study was not influenced by special experience or skills in endocrine pancreatic surgery by one single surgeon or team. Nevertheless, the primary surgical success was complete in all cases and confirmed by a low complication rate and long-term follow-up. Pancreatic and hepatic angiography combined with ASVS can compete with IOUS or the surgeon's searching hands for the best localization test for insulinomas.

Conclusion

This report confirms the high accuracy of the ASVS for preoperative localization of insulinomas. By performing additional ASVS in the distal splenic artery, distal tail insulinomas can be excluded. Separation of body from proximal tail insulinomas seems to necessitate a combined analysis of ASVS and angiography. By omitting insulin sampling in the left hepatic vein and injecting a fixed reduced dose of calcium, ASVS can be simplified, thus increasing its safety and accuracy.

References

1. Kaplan EL, Arganini M, Kang SJ. Diagnosis and treatment of hypoglycemic disorders. *Surg Clin North Am* 1987;67:395–409
2. Galbut DL, Markowitz AM. Insulinoma. Diagnosis, surgical management and long-term follow-up. *Am J Surg* 1980;139:682–690
3. Glickman MH, Hart MJ, White TT. Insulinoma in Seattle: 39 cases in 30 years. *Am J Surg* 1980;140:119–123
4. Stefanini P, Carboni M, Patrassi N, et al. Beta-islet cell tumors of the pancreas: results of a study on 1067 cases. *Surgery* 1974;75:597–609
5. Rothmund M, Angelini L, Brunt LM, et al. Surgery for benign insulinoma: an international review. *World J Surg* 1990;14:393–399
6. Böttger T, Weber W, Beyer J, et al. Wertigkeit der Lokalisationsdiagnostik beim Insulinom. *Med Klin* 1989;84:415–420
7. Dunnick NR, Long JA, Krudy A, et al. Localizing insulinomas with combined radiographic methods. *AJR* 1980;135:747–752
8. Günther R, Kümmerle F, Beyer J, et al. Lokalisationsdiagnostik von Inselzelltumoren durch Sonographie, Computertomographie, Arteriographie und selektive Hormonbestimmung. *Fortschr Röntgenstr* 1981;135:657–662
9. Fulton RE, Sheedy PF, McIlrath DC, et al. Preoperative angiographic localization of insulin producing tumors of the pancreas. *AJR* 1975;123:367–377
10. Galiber AK, Reading CC, Charboneau JW, et al. Localisation of pancreatic insulinoma: comparison of pre- and intraoperative US with CT and angiography. *Radiology* 1988;166:405–408
11. Ingemansson S, Lunderquist A, Lunderquist I, et al. Portal and pancreatic vein catheterization with radioimmunologic determination of insulin. *Surg Gynecol Obstet* 1975;141:705–711
12. Roche A, Raisonnier A, Gillon-Savouret M-C. Pancreatic venous sampling and angiography in localizing insulinomas and gastrinomas. Procedure and results in 55 cases. *Radiology* 1982; 145:621–627
13. Gianello P, Gigot JF, Berthet F, et al. Pre- and intraoperative localization of insulinomas: report of 22 observations. *World J Surg* 1988;12:389–397
14. Vinik AI, Delbridge L, Moattari R, et al. Transhepatic portal vein catheterization for localization of insulinomas: a ten-year experience. *Surgery* 1991;109:1–11
15. Pedrazzoli S, Pasquali C, D'Andrea A. Surgical treatment of insulinoma. *Br J Surg* 1994;81:672–676
16. Semelka RC, Cumming MJ, Shoenut PJ, et al. Islet cell tumors: comparison of dynamic contrast-enhanced CT and MR imaging with dynamic gadolinium enhancement and fat suppression. *Radiology* 1993;186:799–802
17. Kraus BB, Ros PR. Insulinoma: diagnosis with fat-suppressed MR imaging. *AJR* 1994;162:69–70
18. Mori M, Fukuda T, Nagayoshi K, et al. Insulinoma: correlation of short-T1 inversion-recovery (STIR) imaging and histopathologic findings; *Abdom Imaging* 1996;21:337–341
19. Daggett PR, Goodburn EA, Kurtz AB, et al. Is preoperative localisation of insulinomas necessary? *Lancet* 1981;318:483–486
20. van Heerden JA, Grant CS, Czako PF, et al. Occult functioning insulinomas: which localizing studies are indicated? *Surgery* 1992;112:1010–1014
21. Böttger TC, Junginger T. Is preoperative radiographic localization of islet cell tumors in patients with insulinoma necessary? *World J Surg* 1993;17:427–432
22. Rothmund M. Localization of endocrine pancreatic tumours. *Br J Surg* 1994;81:164–166
23. Norton JA, Shawker TH, Doppman JL, et al. Localization and surgical treatment of occult insulinomas. *Ann Surg* 1990; 212:615–620
24. Pasieka JL, McLeod MK, Thompson NW, et al. Surgical approach to insulinomas. Assessing the need for preoperative localization. *Arch Surg* 1992;127:442–447
25. Rösch T, Lightdale CJ, Botet JF, et al. Localization of pancreatic endocrine tumors by endoscopic ultrasonography. *N Engl J Med* 1992;326:1721–1726
26. Doppman JL, Miller DL, Chang R, et al. Insulinomas. Localization with intraarterial injection of calcium. *Radiology* 1991; 178:237–241
27. Gaecke RF, Kaplan EL, Rubenstein A, et al. Insulin and proinsulin release during calcium infusion in a patient with islet-cell tumor. *Metabolism* 1975;24:1029–1034
28. Kaplan EL, Rubenstein A, Evans R, et al. Calcium infusion. A new provocative test for insulinomas. *Ann Surg* 1979;190:501–507
29. Brunt LM, Veldhuis JD, Dilley WG, et al. Stimulation of insulin secretion by a rapid intravenous calcium infusion with β-cell neoplasms of the pancreas. *J Clin Endocrinol Metab* 1986;62:210–216
30. Imamura M, Takahashi K, Adachi H, et al. Usefulness of selective arterial secretin injection test for localization of gastrinoma in the Zollinger-Ellison syndrome. *Ann Surg* 1987;205:230–239
31. Doppman JL, Miller DL, Chang R, et al. Intraarterial calcium stimulation test for detection of insulinomas. *Word J Surg* 1993;17:439–443
32. Doppman JL, Chang R, Fraker DL, et al. Localization of insulinomas to regions of the pancreas by intra-arterial stimulation with calcium. *Ann Intern Med* 1995;123:269–273
33. Klotter HJ, Weinel R, Rothmund M, et al. Intraarterielle Calciumprovokation zur präoperativen Lokalisationsdiagnostik des okkul-

ten Insulinoms [Letter to the editor]. *Deutsche Med Wochenschr* 1993; 118:881–883
34. Axelrod L. Insulinoma: cost-effective care in patients with a rare disease. *Ann Intern Med* 1995; 123:311–312
35. Defreyne L, Moser C, Scheidt T, et al. Intraarterielle Calcium-provokation zur präoperativen Lokalisationsdiagnostik des okkulten insulinoms. *Deutsche Med Wochenschr* 1992; 117:1829–1837
36. Norton JA. Invited commentary on pre- and intraoperative localization of insulinomas. *World J Surg* 1988; 12:396–397
37. O'Shea D, Rohrer-Theus A, Lynn JA, et al. Localization of Insulinomas by selective intraarterial calcium injection. *JCEM* 1996; 4:1623–1627
38. Hayashi T, Honda H, Yasumori K, et al. Selective intra-arterial injection of calcium for localization of insulinomas: proposed new criteria. *Nippon Igaku Hoshasen Gakkai Zasshi* 1995; 55:952–956
39. Ashcroft F, Rorsman P. Electrophysiology of the pancreatic β-cell. *Prog Biophys Molec Biol* 1989; 54:87–143
40. Ashcroft SJH. Glucoreceptor mechanisms and the control of insulin release and biosynthesis. *Diabetologica* 1980; 18:5–15
41. Servive FJ. Hypoglycemic disorders. *N Engl J Med* 1995; 332:1144–1152
42. Jain RK. Transport of molecules in the tumor interstitium: a review. *Cancer Res* 1987; 47:3039–3051
43. Glaser B, Shapiro B, Glowniak J, et al. Effects of secretin on the normal and pathological β-cell. *JCEM* 1988; 66:1138–1143
44. Sobotta-Becher. *Atlas der deskriptiven Anatomie des Menschen. 2. Teil: Die Eingeweide des Menschen einschließlich des Herzens. 15.* Auflage, München-Berlin, Urban & Schwarzenberg, 1962: 78–81
45. Kadir S. *Atlas of normal and variant angiographic anatomy,* 1st ed. Philadelphia: WB Saunders, 1991:303–304
46. Hayward I, Mindelzun RE, Jeffrey RB. Intrapancreatic accessory spleen mimicking pancreatic mass on CT. *J Comput Assist Tomogr* 1992; 16:984–985
47. Harris GN, Kase DJ, Bradnock H, et al. Accessory spleen causing a mass in the tail of the pancreas: MR imaging findings. *AJR* 1994; 163:1120–1121
48. King CMP, Reznek RH, Dacie JE, et al. Review imaging islet cell tumours. *Clin Radiol* 1994; 49:295–303
49. Gibril F, Doppman JL, Chang R, et al. Metastatic gastrinomas: localization with selective arterial injection of secretin. *Radiology* 1996; 198:77–84
