# Supplementary material for: Diagnostic value of ASVS for insulinoma localization: A systematic review and meta-analysis
Source: PLoS One. 2019 Nov 19;14(11):e0224928. doi: 10.1371/journal.pone.0224928 (PMC6863549; doi:10.1371/journal.pone.0224928)
Supplement: S2 File — (ZIP) [file pone.0224928.s002.zip › included studies/Preoperative localization of an insulinoma.pdf]

J Endocrinol Invest
DOI 10.1007/s40618-015-0406-4

ORIGINAL ARTICLE

Preoperative localization of an insulinoma: selective arterial calcium stimulation test performance

J. Morera[1] · A. Guillaume[1] · P. Courtheoux[2] · L. Palazzo[3] · A. Rod[1] · M. Joubert[1] · Y. Reznik[1]

Received: 28 June 2015 / Accepted: 29 October 2015
© Italian Society of Endocrinology (SIE) 2015

Abstract
Purpose Preoperative localization of an insulinoma is recommended to improve the cure rate, but non-invasive procedures can fail to detect the tumour. The objective of the study was to assess the performance of a selective arterial calcium stimulation test in the preoperative localization of insulinomas that were not detected by conventional imaging procedures.
Methods We conducted a monocenter retrospective case review of 13 patients who had endogenous hyperinsulinism and were treated between 1994 and 2013. Patients were selected on the basis of negative or doubtful non-invasive preoperative imaging. A selective arterial calcium stimulation test was performed by pancreatic and hepatic arteriography with selective intra-arterial calcium stimulation and hepatic venous sampling in order to obtain the plasma insulin measurement. We evaluated the efficacy of the test by comparing the results with an endoscopic ultrasound.
Results Twelve of the 13 patients underwent surgery, and the presence of an insulinoma was proven in 11 patients by pathological analysis of the tumour. An endoscopic ultrasound was consistent with surgery in 71.4 % of cases, while selective arterial calcium stimulation was consistent with surgery in 90.9 % and allowed detection of an insulinoma in two additional patients with a negative endoscopic ultrasound. One false-negative and one false-positive arterial calcium test were observed. No adverse events were recorded except transient skin flush following calcium injection in one patient.
Conclusion The selective arterial calcium stimulation test is a sensitive diagnostic procedure for localizing insulinomas and may be considered when non-invasive radiological imaging does not allow the detection of an occult insulinoma.

Keywords Endocrine pancreatic tumour · Hyperinsulinemic hypoglycaemia · Arteriography · Angiography

Introduction

Symptomatic hypoglycaemia in apparently healthy non-diabetic patients often results from an insulinoma; an insulinoma is a small endocrine tumour of the pancreas that may be malignant in approximately 10 % of cases [1].

The diagnosis of an insulinoma requires evidence of clinical and biochemical hypoglycaemia and localization or regionalization of the tumour [2, 3]. Surgical resection is the first-line approach for treating an insulinoma, and its success is highly dependent on preoperative or peri-operative localization of the tumour. In the absence of preoperative localization, insulinomas may be missed peri-operatively in 10–20 % of patients [4–6]. To circumvent this risk, a wide range of imaging procedures has been developed in order to preoperatively visualize pancreatic insulinomas. Available non-invasive procedures include ultrasonography (US), computed tomography (CT) and magnetic resonance imaging (MRI); the latter allows a detection rate of approximatively

✉ Y. Reznik
reznik-y@chu-caen.fr

1 Endocrinology Unit, Centre Hospitalo-Universitaire de Caen, Avenue de la côte de Nacre, CS 30001, 14033 Caen Cedex 9, France

2 Diagnostic Radiology Unit, Centre Hospitalo-Universitaire de Caen, Avenue de la côte de nacre, CS 30001, 14033 Caen Cedex 9, France

3 Endoscopic Ultrasound Unit, Trocadero Clinic, 75016 Paris, France

Published online: 17 November 2015

40 % and 70–85 % for the two previous procedures [7, 8]. Tumour imaging methods based on peptide receptor targeting may be an interesting option, especially for the detection of ectopic insulinomas. These tumours represent 1–2 % of all insulinomas [9] and are commonly located in the peri-pancreatic or peri-duodenal regions [10]. Somatostatin analogue-related imaging (e.g. ^{111}Inpentetreotide scintigraphy [11–13], ^{68}Gallium-DOTATATE positron emission tomography (PET), ^{68}Ga-DOTATOC-PET and ^{68}Ga-DOTANOC-PET [14–17]) exhibits high sensitivity for the detection of neuroendocrine tumours but are less accurate for the detection of insulinomas [18]. Other tracers are used for PET imaging, such as ^{11}C-5-Hydroxytryptophan-PET (C5-HTP-PET), with a high sensitivity for the detection of pancreatic neuroendocrine tumours. Nevertheless, no data are available concerning insulinoma detection [19]. Some authors [20], but not all [21], found that 18F-DOPA-PET may help detect nesidioblastosis or insulinoma. ^{11}In-DOTA-exendin-4-PET is also promising for the detection of benign but not malignant insulinomas [22, 23]. Invasive techniques may be offered to patients with endogenous hyperinsulinism that is ineffectively explored by current radiological procedures. Both endoscopic ultrasounds (EUSs) and selective arterial calcium stimulation tests (SACSTs) with hepatic venous sampling may be offered for localization of an occult insulinoma [24–26]; the latter is the procedure of choice for confirming non-insulinoma pancreatogenous hyperinsulinemia [18, 27].

SACST was first described by Doppman et al. and was proven effective for localizing insulinomas of the head or the body–tail region of the pancreas [24]. This dynamic testing consists of serial selective catheterization of the gastroduodenal, superior mesenteric and splenic arteries and then calcium gluconate infusion into the arterial vessels together with blood sampling of the hepatic venous effluent for insulin concentration determination. The procedure is rationalized with the observation that calcium stimulates the release of insulin from tumoural but not normal β cells. Few studies have evaluated the respective performances of SACST and EUS in situations of proven endogenous hyperinsulinemia [28–31]; no studies have evaluated the performance of SACST and EUS in cases with occult insulinomas with non-invasive imaging failure [32–34]. In the present study, we report our monocenter experience of the SACST procedure for localization of an insulinoma in 13 patients in whom non-invasive imaging techniques failed or were doubtful and compare the performances of SACST and EUS.

Materials and methods

Forty-eight patients with symptomatic hyperinsulinemic hypoglycaemia were investigated in the Division of Endocrinology at Caen University Hospital during the period from January 1978 until April 2013. Diagnosis of hyperinsulinemic hypoglycaemia was based on the occurrence of severe hypoglycaemic symptoms together with low fasting serum glucose levels (<50 mg/dl) and biochemical criteria of inappropriate hyperinsulinemia during a 72-h fasting test, i.e. symptomatic hypoglycaemia below 45 mg/dl together with plasma insulin level >3 mU/l and/or C peptide level >0.2 nmol/l and/or proinsulin level >5 pmol/l. Fasting was discontinued when the patient experienced significant neuroglycopenic symptoms or after 72 h.

Once the diagnosis of hyperinsulinemic hypoglycaemia was made, patients underwent preoperative localization by US, CT and/or MRI. Until 2005, one or several non-invasive radiological imaging procedures were performed, and SACST was offered in cases with negative or discordant findings. Starting in 2005, our centre referred patients with hyperinsulinemic hypoglycaemia to a physician with paramount expertise on EUS localization of insulinomas (L.P.), and EUS and/or SACST was performed for occult insulinomas.

SACST is a procedure derived from pancreatic angiography and is based on the stimulation of β cell tumours by a secretagogue, i.e. calcium gluconate. To sample hepatic venous blood, a five French catheter is inserted transfemorally into the right hepatic vein. The contralateral femoral artery is then punctured, and a five French catheter is introduced into the coeliac trunk and the mesenteric artery. Pancreatic and hepatic angiography consists of the selective catheterization of (1) the splenic artery (SA) supplying the body and tail and (2) the gastroduodenal artery (GDA) and the superior mesenteric artery (SMA) supplying the head of the pancreas. After each angiographic catheterization, calcium gluconate is injected intra-arterially at a dose of 0.025 mEq/kg body weight (i.e. 5.6 mg/kg) to evoke insulin secretion by the β cell tumour. Before (T0) and 20, 40 and 60 s after each arterial injection, 5 ml of hepatic vein blood was sampled. Blood samples, which were labelled with the time interval and the type of catheterized artery, were stored at 4 °C. Insulin concentrations were measured by an immunoradiometric assay (Cisbio®, France). All tests were carried out under local anaesthesia. A positive response to the calcium injection was defined as the doubling of insulin concentration from baseline with an insulin peak >100 µU/ml (this criteria was established in order to exclude false-positive patients with very low basal and stimulated insulin level). A glucose intravenous infusion (5 % glucose) was maintained during the SACST procedure in order to avoid hypoglycaemic episodes.

Results

Among the forty-eight patients investigated for hyperinsulinemic hypoglycaemia during the study period,

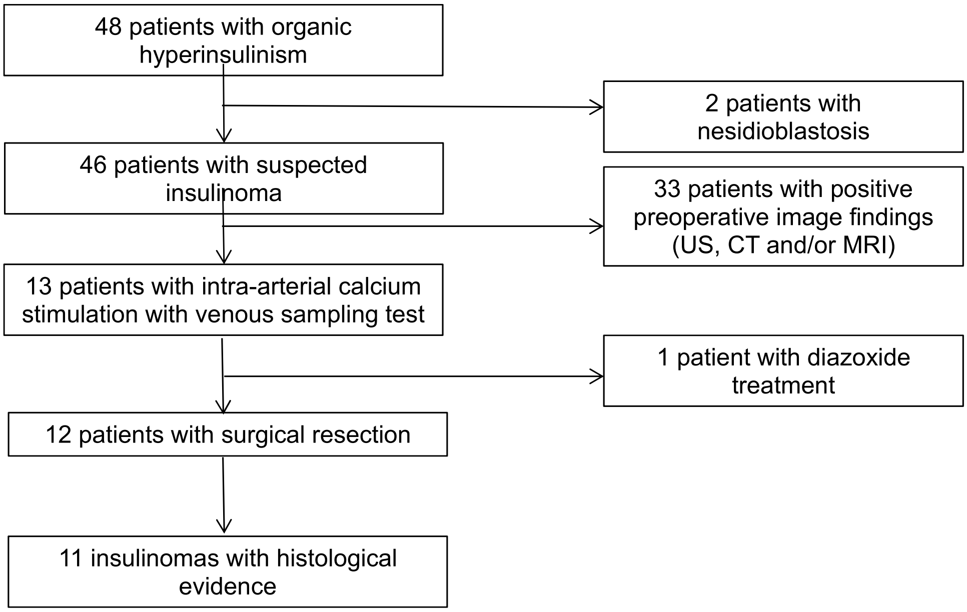

Fig. 1 Patients with organic hypoglycaemia in the endocrinology unit

non-invasive localization procedures localized an insulinoma in 33 patients and failed in 13 patients. Sensitivities of US, CT and MRI for tumour localization were 14, 48 and 46 %, respectively, compared with surgical localization. Two patients with nesidioblastosis were excluded from the study (Fig. 1). EUS and/or SACST was performed in these 13 patients including 10 women and 3 men, with an age range at diagnosis between 22 and 76 years (mean 53.2 ± 17.9 years). In all but one patient, symptomatic hypoglycaemia was proven during a 72-h fasting test (mean plasma glucose nadir 35 ± 5 mg/dl) after a fasting period ranging between 8 and 30 h (mean 18.1 ± 7.5 h). Insulin levels were unsuppressed at the time of the nadir glucose level, ranging from 3.1 to 32 mU/l (mean insulin level 9.3 ± 9.2 mU/l). In one patient, no hypoglycaemia occurred during fasting but hyperinsulinemic hypoglycaemia was detected after a meal (glucose level 40 mg/dl together with insulin level 100 mU/l). None of the 13 patients had multiple endocrine neoplasia type 1 syndrome.

In all patients, at least one non-invasive imaging procedure was performed preoperatively (mean number 2.64 procedures, $n = 10$ having 3 procedures, $n = 1$ having 2 procedures and $n = 2$ having 1 procedure); because of inconclusive results, EUS and/or SACST was subsequently performed (Table 1). In all patients, catheterization of the three arteries supplying the pancreas was successful. Table 2 summarizes the results of SACST. Overall, the insulin level in the right hepatic vein increased after calcium injection in the pancreatic arteries by 2.2- to 28-fold from the baseline insulin level, and the mean insulin peak measured in the right hepatic vein after the calcium bolus was 287 ± 293 mU/l (range 26–1040). In six patients (Tables 1, 2, patients nb 1, 2, 6, 7, 9, 10), SACST evoked a doubling from the baseline of insulin concentration in one sole pancreatic artery, indicating the presence of an insulinoma in the corresponding territory, and surgery confirmed the presence of an insulinoma in all cases (Table 1). In patient 3, an insulin level increase after SACST was observed in GDA and SMA; both vessels supplied the head of the pancreas. In patient 4, GDA SACST evoked a 3.2-fold insulin level increase together with a high basal insulin level (218 mU/l), suggesting the presence of an insulinoma in the head of the pancreas. The threefold insulin level increase during SA SACST was considered to be a false positive because both basal and stimulated insulin concentrations were very low (9 and 31 mU/l, respectively). In patient 5, all non-invasive procedures and EUS were negative. An increase in insulin level after SACST was observed in the three arteries, i.e. GDA, SMA and SA, but angiography showed an opacification of the SA after an iodine contrast injection in the GDA and the SMA, suggesting an anatomical arterial variation. It was therefore concluded that the insulinoma might be localized in the body or the tail of the pancreas. Thereafter, a second EUS was performed by another physician, with paramount expertise on EUS localization of insulinomas, which allowed identification of a solitary insulinoma in the tail of the pancreas. Tumour identification was confirmed by surgery. Such a case exemplifies the importance of the operator's expertise for EUS sensitivity. In patient 8, the SACST of two arteries supplying different territories of the pancreas evoked a positive response, but the gradient in the SMA was considered to be a false positive because basal and stimulated insulin concentrations were low. It was concluded that the insulinoma might be localized in the body or the tail of the pancreas; this result was confirmed by surgery. In patient

Table 1 Individual preoperative imaging, selective arterial calcium stimulation test and surgery data from the 13 patients

Patient	Year of diagnosis	US	CT	MRI	EUS	Conclusion angiography/SACST	IOUS	IOP	Surgery/location/size (mm)	Concordance SACST/surgery
1	1994	–	–	–	nd	Head	++	++	E/head/18	Yes
2	1995	–	–	–	–	Head	++	++	E/head/14	Yes
3	1995	–	–	–	–	Head	++	++	E/head/10	Yes
4	1997	–	–	nd	nd	Head	++	++	E/head/15	Yes
5	1997	–	–	–	0 (EUS no. 1) ++(EUS no. 2 performed after SACST)	Body or tail	++	++	DP/tail/10	Yes
6	2004	–	–	–	nd	Head	++	++	CDP/head/7	Yes
7	2004	–	–	–	nd	Body or tail	–	++	DP/tail/12	Yes
8	2004	–	–	–	++(EUS performed after SACST in 2005)	Body or tail (performed before EUS)	nd	++	E/tail/20	Yes
9	2007	–	+(tumour no. 1) ++(tumour no. 2)	–	++(tumour no. 2) (EUS performed after SACST)	Body or tail (performed before EUS)	++ (tumour no. 2)	++ (tumour no. 2)	DP (tumour no. 2)/body/12	Yes
10	2010	+ (tumour no. 1)	–	++ (tumour no. 2)	+(tumour no. 1) ++(tumour no. 2)	Body or tail	nd	–	DP (tumour no. 2)/tail/3 and 7	Yes
11	2011	–	–	–	+	Not conclusive	NA	NA	No	NA
12	2012	nd	nd	–	+	Not conclusive	–	–	E/extra-pancreatic/17 No histological evidence	NA
13	2013	nd	nd	–	++	Not conclusive	nd	++(head)	CDP/head/14	No

US ultrasonography, *CT* computed tomography, *MRI* magnetic resonance imaging, *EUS* endoscopy ultrasound, *SACST* selective arterial calcium stimulation test with venous sampling, *IOUS* intra-operative ultrasound, *IOP* intra-operative palpation, *Nd* not done, – no tumour seen or palpated, + lesion not characteristic of an adenoma, ++ lesion characteristic of an adenoma, *NA* not applicable, *E* enucleation, *DP* distal pancreatectomy, *CDP* cephalic duodenopancreatectomy

Table 2 Results of SACST listed per patient and per artery

Patients	1	2	3	4	5	6	7	8	9	10	11	12	13
Arterial gradient													
Superior mesenteric artery	28	0	5.4	0	34	0	0	2.6	0	0	0	0	0
Gastroduodenal artery	0	10	4.6	3.2	7.1	4	0	0	0	0	14.6	2.2	0
Splenic artery	0	0	0	3.3	12	0	3	13.4	10.5	9.8	4	0	0
Localization according gradient	Head	Head	Head	NC	NC	Head	Body or tail	NC	Body or tail	Body or tail	NC	Head	NC
Remarks	–	–	–	Very low basal and stimulated insulin level in SA	Anatomical arterial variation	–	–	Very low basal and stimulated insulin level in SMA	–	–	–	Very low basal and stimulated insulin level in GDA	
Conclusion SACST at the time of examination	Head	Head	Head	Head	Body or tail	Head	Body or tail	Body or tail	Body or tail	Body or tail	NC	NC	NC

Insulin gradients were measured as the ratio of venous hepatic insulin concentration at 20, 40 or 60 s after SACST to the insulin concentration before SACST

0 gradient < 2, *NC* not conclusive

11, an insulin level increase after SACST was observed in GDA and SA without technical pitfalls or anatomical variation explaining such misleading results; SACST was considered to be inconclusive. All imaging procedures failed to localize the tumour. The search for occult sulfonylurea consumption was negative, and a course with diazoxide successfully prevented the occurrence of hypoglycaemia until the present time. Due to patient's unwillingness to undergo surgery and diazoxide efficacy for preventing hypoglycaemia, no surgery was planned and long-term medical therapy and follow-up were offered to the patient. In patient 12, SACST was considered to be inconclusive because basal and stimulated insulin concentrations were low. EUS localized an atypical hyperechoic nodule extrinsic to the pancreas tail. After enucleation of the nodule, hypoglycaemia resolved, but histological examination showed no adenoma but only normal pancreas tissue. Other causes of hyperinsulinemic hypoglycaemia—autoimmune, drug induced—were excluded, suggesting the removal of an ectopic insulinoma during surgery, although histological assessment could not be obtained. In patient 13, no response to SACST in the three arteries was observed 1 month after diazoxide removal. EUS identified a solitary insulinoma in the head of the pancreas, and a cephalic duodenopancreatectomy confirmed the presence of an insulinoma.

No adverse event was recorded with SACST except a transient skin flush following calcium injection in one patient. Neither hypoglycaemia nor hypercalcaemia was recorded.

Twelve out of the 13 patients underwent surgical resection of an insulinoma. Most proven insulinomas were localized in the head (55 %), while 9 % were found in the body and 36 % were in the tail of the pancreas. The mean diameter of resected tumours was 12.2 ± 4.8 mm (range 3–20 mm). Two tumours were found in patient 10 (3 and 7 mm). In five patients (45.4 %), the insulinoma was resected by enucleation, in four patients (36.4 %) by distal pancreatectomy and in two (18.2 %) by cephalic duodenopancreatectomy. No recurrence of hypoglycaemia occurred during the follow-up period. In one patient (nb 12), no histological evidence of an insulinoma was obtained.

Intra-operative palpation (IOP) was systematically performed by the surgeon and correctly localized a histologically proven insulinoma in 10/11 patients (90.9 % sensitivity) (Table 1). In patient 10, the two small tumours inside the pancreas tail were not localized by intra-operative localization techniques. Intra-operative ultrasound (IOUS) was performed in 9 patients and exhibited a sensitivity of 87.5 %.

Overall, angiography coupled to SACST was concordant with surgery for the localization of 10/11 proven insulinomas (90.9 % sensitivity) (Table 1). EUS was performed 10 times in 9 patients and was concordant with surgery in

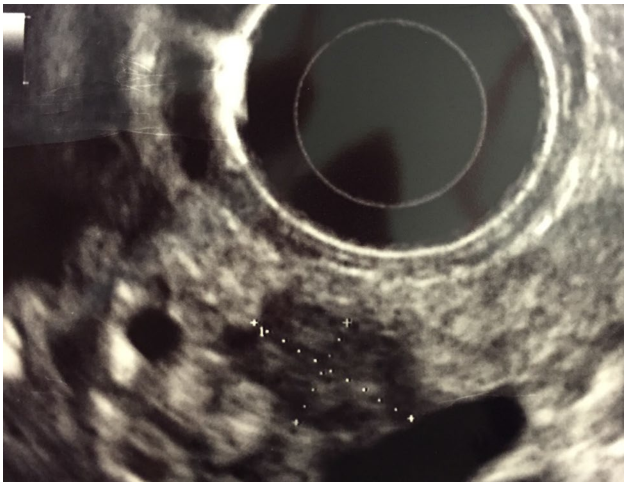

Fig. 2 Positive endoscopy ultrasound in patient nb 9 (L.P.) showing a solitary nodule of 10 mm in the body of the pancreas. A selective arterial calcium simulation test was performed and showed an insulin gradient of 10.5 in the splenic artery, supplying the body or the tail of the pancreas (insulinemia level of 16.4, 85.7, 173 and 164 mUI/l before calcium injection and at 20, 40 and 60 s after injection, respectively). EUS and SACST were both concordant with surgery, confirming the presence of a 12-mm-diameter insulinoma in the body of the pancreas

5/7 cases (71.4 % sensitivity). Figure 2 illustrates concordant EUS and SACST tumour localization. In two cases (patients 2 and 3), SACST detected an insulinoma while EUS did not. In patient 13, EUS detected an insulinoma while SACST did not.

Discussion and conclusions

Surgical removal is the treatment of choice for islet cell tumours that are responsible of hyperinsulinemic hypoglycaemia. A large number of insulinomas are identified intraoperatively by experienced surgeons. However, reports of undetected insulinomas were recorded in 10–27 % of surgical procedures in several studies [5, 6]; preoperative imaging enables more accurate surgical localization and sometimes avoids unnecessary extensive pancreatectomy.

Different localization procedures are available including US, CT, MRI, SACST, EUS and somatostatin scintigraphy. The Endocrine Society clinical practice guidelines suggest that US, CT and/or MRI should be the first choice for imaging techniques for insulinoma localization [26, 35]. Trans-abdominal US is a non-invasive procedure that is both readily available and inexpensive, but it is highly dependent on operator expertise and lacks sensitivity in overweight or obese patients. These limitations explain its variable sensitivity (14–100 %) [31, 36, 37] although sensitivity should be enhanced when using contrast-enhanced ultrasonography [38]. CT scanning is a non-invasive imaging technique that exhibits 24–66 % sensitivity [24, 28, 29, 31, 35, 36, 39, 40]. MRI has a higher sensitivity depending on operator expertise (42–82 %) and may detect secondary tumours from malignant insulinomas [29, 31, 35, 39, 41]. The comparative results of US, CT and MRI performed in our patients are in accordance with previous series. Somatostatin receptor (SSTR) scintigraphy (using ^{111}Inpentetreotide) exhibits low sensitivity (33–50 %) in most series [29, 35, 42], but not in all [43]. ^{68}Gallium-labelled somatostatin analogue (^{68}Ga-DOTATATE, ^{68}Ga-DOTATOC and ^{68}Ga-DOTANOC) positron emission tomography combined with CT (PET/CT) exhibits a higher sensitivity for detecting neuroendocrine tumours [14, 15], but no studies were performed in insulinomas that currently express SST2R in only 50 % of cases [44]. CT-coupled ^{11}C-5-Hydroxytryptophan-PET (C5-HTP-PET) exhibits a 100 % sensitivity for detection of pancreatic neuroendocrine tumours but data on insulinoma detection are lacking [19]. 18F-DOPA-PET is a promising technique that identified an insulinoma tumour in 9/10 cases in a study by Kauhanen et al. [20]. Such performance was not observed in another study [21]. ^{11}In-DOTA-exendin-4-PET may also be used for the detection of benign but not malignant insulinomas [22, 23]. Overall, these techniques may be helpful for tumour detection, but they are expensive and not readily available. Overall, insulinomas less than 10 mm in diameter are often missed by non-invasive techniques [8]. In such situations, invasive imaging is mandatory and EUS or SACST may be offered for localizing occult islet cell tumours, a situation occurring in approximately 20 % of cases in a large recent series [31, 35] but may occur in up to 75 % of cases, even in tertiary care experimental centres [32]. EUS is often chosen as a second- or first-line imaging tool for localizing insulinomas [18] because of its high spatial resolution that enables detection and precise anatomical location of very small lesions [45] with sensitivities between 75 and 94 % [29, 31, 41, 46]. Nevertheless, the technique demands considerable expertise (e.g. patient nb 5), and the technique is not available in all centres.

SACST combines angiography imaging and biochemical data from calcium-evoked stimulation of tumoural insulin secretion. Insulinomas may be visualized by angiography as a well-defined round or oval vascular blush corresponding to densified vascularity in comparison with normal healthy pancreatic parenchyma surrounding the tumour. Insulinomas are observed during the early arterial phase and during a variable length of the venous phase of the run [24]. SACST generally does not precisely localize the tumour but rather indicates whether the insulinoma is located on the right side (i.e. pancreatic head and neck) or the left side (i.e. pancreatic body and tail) of the superior mesenteric artery, independent of the tumour size [28].

Such information is relevant for choosing the surgical approach because a tumour located to the left of the SMA might be enucleated or might demand a distal pancreatectomy, whereas a tumour located to the right of the SMA might be enucleated or might be an indication for cephalic duodenal pancreatectomy.

SACST superiority over non-invasive imaging procedures for localizing an insulinoma has been suggested by previous studies [29, 39] and was confirmed in the present study with a 90.9 % sensitivity, a value comparable to the 78–100 % sensitivity found in previous series [28–30, 32, 33, 39, 40, 47]. Two recent studies performed in centres with high technical expertise also found a higher sensitivity of SACST (93 and 100 %) than EUS (75 and 77 %) for localizing an insulinoma [29, 31]. In our series, SACST and EU exhibit high sensitivities and may have similar performance when EUS is performed by a physician who has been highly trained in using the technique. Nevertheless, SACST and EUS are complementary techniques for detecting an occult insulinoma.

In our study, SACST sensitivity was similar to that of palpation combined with IOUS, which is considered to be the most effective method for localizing an insulinoma [48, 49]. However, IOUS was not performed in all patients from our series, suggesting possible underestimation of the performance of this technique. A high percentage of insulinomas can be identified intra-operatively in an experienced surgeon's hands. However, insulinomas may be missed peri-operatively in 10–20 % of patients [4–6] for whom preoperative investigations are of paramount importance for tumour localization.

In our study, no adverse events were observed with SACST; this was in agreement with previous studies. Hypoglycaemia is a rare event during SACST that has only been reported twice in more than 104 patients [24, 28, 30, 32–34, 48]. No adverse events related to the angiographic procedure were reported in our series or in other series.

SACST has some pitfalls and limitations. Multiple secretory bursts were observed after serial artery calcium injection in four out of 13 patients. In a large series of 45 SACSTs, Guettier et al. also found a 50 % rate of multiple secretory responses [32]. Such inconsistencies may relate to an overlap of arterial territories for tumoural blood supply, the presence of multiple insulinomas, erratic tumoural secretory behaviour or a lack of specificity of secretory bursts by normal pancreas territories. To circumvent false-positive responses to SACST, some authors recommend defining the insulin peak cut-off for interpreting SACST, as in our current practice [50]. Other authors suggest that the strongest response evoked by SACST may be chosen for the localization of the tumour [32]. The SACST procedure may also be repeated to identify the occult insulinoma after a first negative or inconsistent SACST [29].

In conclusion, our data confirm that SACST is an invasive but innocuous second-line procedure for localizing insulinomas that are unidentified by non-invasive imaging. SACST may be preferred to EUS in centres that are unable to provide paramount EUS expertise for detecting occult insulinomas. In expert centres, SACST and EUS are complementary procedures and sometimes may be coupled to reach maximal diagnostic performance.

Compliance with ethical standards

Conflict of interest The authors declare that they have no conflict of interest.

Ethical approval All procedures performed in studies involving human participants were in accordance with the ethical standards of the institutional ethic committee and with the 1964 Helsinki Declaration and its later amendments or comparable ethical standards.

Informed consent For this type of study (retrospective study), formal consent is not required.

References

1. Service FJ, McMahon MM, O'Brien PC, Ballard DJ (1991) Functioning insulinoma—incidence, recurrence and long-term survival of patients: a 60-year study. Mayo Clin Proc 66:711–719
2. Guettier JM, Gorden P (2006) Hypoglycemia. Endocrinol Metab Clin North Am 35:753–766
3. Service FJ (1995) Hypoglycemic disorders. N Engl J Med 332:1144–1152
4. Daggett PR, Goodburn EA, Kurtz AB, Le Quesne LP, Morris DV, Nabarro JD, Raphael MJ (1981) Is preoperative localisation of insulinomas necessary? Lancet 140:483–486
5. Angelini L, Bezzi M, Tucci G, Lirici MM, Candiani F, Rubaltelli L, Tremolada C, Tamburrano G, Fegiz G (1987) The ultrasonic detection of insulinomas during surgical exploration of the pancreas. World J Surg 11:642–647
6. Gower WR Jr, Fabri PJ (1990) Endocrine neoplasm (non-gastrin) of the pancreas. Semin Surg Oncol 6:98–109
7. Kaltsas GA, Besser GM, Grossman AB (2004) The diagnosis and medical management of advanced neuroendocrine tumors. Endocr Rev 25:458–511
8. Noone TC, Hosey J, Firat Z, Semelka RC (2005) Imaging and localization of islet-cell tumours of the pancreas on CT and MRI. Best Pract Res Clin Endocrinol Metab 19:195–211
9. Filipi CJ, Higgins GA (1973) Diagnosis and management of insulinomas. J Am Surg 125:231–239
10. Ramkumar S, Dhingra A, Jyotsna V, Ganie MA, Das CJ, Seth A, Sharma MC, Bal CS (2014) Ectopic insulin secreting neuroendocrine tumor of kidney, with recurrent hypoglycaemia: a diagnostic dilemma. BMC Endocrine Disord 34:36
11. Van der Lely AJ, de Herder WW, Krenning EP, Kwekkeboom DJ (2003) Octreoscan radioreceptor imaging. Endocrine 20:307–311
12. Kwekkeboom DJ, Krenning EP, Scheidhauer K, Lewington V, Lebtahi R, Grossman A, Vitek P, Sundin A, Plöckinger U (2009) ENETS consensus guidelines for the standards of care in neuroendocrine tumors: somatostatin receptor imaging with (111) In-pentetreotide. Neuroendocrinology 90:184–189

13. Perri M, Erba P, Volterrani D, Lazzeri E, Boni G, Grosso M, Mariani G (2008) Octreo-SPECT/CT imaging for accurate detection and localization of suspected neuroendocrine tumors. Q J Nucl Med Mol Imaging 52:323–333
14. Buchmann I, Henze M, Engelbrecht S, Eisenhut M M, Runz A, Schäfer M, Schilling T, Haufe S, Herrmann T, Haberkorn U (2007) Comparison of 68 Ga-DOTATOC PET and 111In-DTPAOC (Octreoscan) SPECT in patients with neuroendocrine tumours. Eur J Nucl Med Mol Imaging 34:1617–1626
15. Gabriel M, Decristoforo C, Kendler D, Dobrozemsky G, Heute D, Uprimny C, Kovacs P, Von Guggenberg E, Bale R, Virgolini IJ (2007) 68 Ga-DOTA-Tyr3-octreotide PET in neuroendocrine tumours: comparison with somatostatin receptor scintigraphy and CT. J Nucl Med 48:508–518
16. Haug AR, Clindea-Drimus R, Auernhammer CJ, Reincke M, Wängler B, Uebleis C, Schmidt GP, Göke B, Bartenstein P, Hacker M (2012) The role of 68 Ga-DOTATATE PET/CT in suspected neuroendocrine tumors. J Nucl Med 53:1686–1692
17. Prasad V, Ambrosini V, Hommann M, Hoersch D, Fanti S, Baum RP (2010) Detection of unknown primary neuroendocrine tumours (CUP-NET) using (68)Ga-DOTA-NOC receptor PETC/CT. Eur J Nucl Med Mol Imaging 37:67–77
18. Grimaldi F, Fazio N, Attanasio R, Frasoldati A, Papini E, Angelini F, Baldelli R, Berretti D, Bianchetti S, Bizzarri G, Caputo M, Castello R, Cremonini N, Crescenzi A, Davì MV, D'Elia AV, Faggiano A, Pizzolitto S, Versari A, Zini M, Rindi G, Oberg K (2014) Italian Association of Clinical Endocrinologists (AME) position statement: a stepwise clinical approach to the diagnosis of gastroenteropancreatic neuroendocrine neoplasms. J Endocrinol Invest 37:875–909
19. Koopmans KP, Neels OC, Kema IP, Elsinga PH, Sluiter WJ, Vanghillewe K, Brouwers AH, Jager PL, de Vries EG (2008) Improved staging of patients with carcinoid and islet cell tumors with 18F-dihydroxy-phenyl-alanine and 11C-5-hydroxy-tryptophan positron emission tomography. J Clin Oncol 26:1489–1495
20. Kauhanen S, Seppänen M, Minn H, Gullichsen R, Salonen A, Alanen K, Parkkola R, Solin O, Bergman J, Sane T, Salmi J, Välimäki M, Nuutila P (2007) Fluorine-18-L-dihydroxyphenylalanine (18F-DOPA) positron emission tomography as a tool to localize an insulinoma or beta-cell hyperplasia in adult patients. J Clin Endocrinol Metab 92:1237–1244
21. Tessonnier L, Sebag F, Ghander C, De Micco C, Reynaud R, Palazzo FF, Conte-Devolx B, Henry JF, Mundler O, Taïeb D (2010) Limited value of 18F-F-DOPA PET to localize pancreatic insulin-secreting tumors in adults with hyperinsulinemic hypoglycemia. J Clin Endocrinol Metab 95:303–307
22. Christ E, Wild D, Forrer F, Brändle C, De Micco C, Reynaud R, Palazzo FF, Conte-Delvox B, Henry JF, Mundler O, Taïeb D (2010) Limited value of 18F-F-DOPA PET to localize pancreatic insulin-secreting tumors in adults with hyperinsulinemic hypoglycaemia. J Clin Endocrinol Metab 94:4398–4405
23. Wild D, Christ E, Caplin ME, Kurzawinski TR, Forrer F, Brändle M, Seufert J, Weber WA, Bomanji J, Perren A, Ell PJ, Reubi JC (2011) Glucagon-like peptide-1 versus somatostatin receptor targeting reveals 2 distinct forms of malignant insulinomas. J Nucl Med 52:1073–1078
24. Doppman JL, Miller DL, Chang R, Shawker TH, Gorden P, Norton JA (1991) Insulinomas: localization with selective intraarterial injection of calcium. Radiology 178:237–241
25. Rösch T, Lightdale CJ, Botet JF, Boyce GA, Sivak MV Jr, Yasuda K, Heyder N, Palazzo L, Dancygier H, Schusdziarra V, Classen M (1992) Localization of pancreatic endocrine tumors by endoscopic ultrasonography. N Engl J Med 326:1721–1726
26. Cryer PE, Axelrod L, Grossman AB, Heller SR, Montori VM, Seaquist ER, Service FJ (2009) Evaluation and management of adult hypoglycemic disorders: an Endocrine Society Clinical Practice Guideline. J Clin Endocrinol Metab 94:709–728
27. Won JG, Tseng HS, Yang AH, Tang KT, Jap TS, Lee CH, Lin HD, Burcus N, Pittenger G, Vinik A (2006) Clinical features and morphological characterization of 10 patients with noninsulinoma pancreatogenous hypoglycemia syndrome (NIPHS). Clin Endocrinol (Oxf) 65:566–578
28. Chavan A, Kirschoff TD, Brabant G, Scheumann GFW, Wagner S, Galanski M (2000) Role of the intra-arterial calcium stimulation test in the preoperative localization of insulinomas. Eur Radiol 10:1582–1586
29. Morganstein DL, Lewis DH, Jackson J, Isla A, Lynn J, Devendra D, Meeran K, Todd JF (2009) The role of arterial stimulation and simultaneous venous sampling in addition to cross-sectional imaging for localisation of biochemically proven insulinoma. Eur Radiol 19:2467–2473
30. Defreyne L, König K, Lerch MM, Hesse UJ, Rottiers R, Feifel G, de Hemptinne B, Kramann B, Kunnen M (1998) Modified intra-arterial calcium stimulation with venous sampling test for preoperative localization of insulinomas. Abdom Imaging 23:322–331
31. Placzkowski KA, Vella A, Thompson GB, Grant CS, Reading CC, Charboneau JW, Andrews JC, Lloyd RV, Service FJ (2009) Secular trends in the presentation and management of functioning insulinoma at the Mayo Clinic, 1987–2007. J Clin Endocrinol Metab 94:1069–1073
32. Guettier JM, Kam A, Chang R, Skarulis MC, Cochran C, Alexander HR, Libutti SK, Pingpank JF, Gorden P (2009) Localization of insulinomas to regions of the pancreas by intraarterial calcium stimulation: the NIH experience. J Clin Endocrinol Metab 94:1074–1080
33. Pereira PL, Roche AJ, Maier GW, Huppert PE, Dammann F, Farnsworth CT, Duda SH, Claussen CD (1998) Insulinoma and islet cell hyperplasia: value of the calcium intraarterial stimulation test when findings of other preoperative studies are negative. Radiology 206:703–709
34. O'Shea D, Rohrer-Theurs AW, Lynn JA, Jackson JE, Bloom SR (1996) Localization of insulinomas by selective intraarterial calcium injection. J Clin Endocrinol Metab 81:1623–1627
35. Druce MR, Muthuppalaniappan VM, O'Leary B, Chew SL, Drake WM, Monson JP, Akker SA, Besser M, Sahdev A, Rockall A, Vyas S, Bhattacharya S, Matson M, Berney D, Reznek RH, Grossman AB (2010) Diagnosis and localisation of insulinoma: the value of modern magnetic resonance imaging in conjunction with calcium stimulation catheterization. Eur J Endocrinol 162:971–978
36. Kuzin NM, Egorov AV, Kondrashin SA, Lotov AN, Kuznetzov NS, Majorova JB (1998) Preoperative and intraoperative topographic diagnosis of insulinomas. World J Surg 22:593–598
37. Mehrabi A, Fischer L, Hafezi M, Dirlewanger A, Grenacher L, Diener MK, Fonouni H, Golriz M, Garoussi C, Fard N, Rahbari NN, Werner J, Büchler MW (2014) A systematic review of localization, surgical treatment options and outcome of insulinoma. Pancreas 43:675–686
38. An L, Li W, Yao KC, Liu R, Lv F, Tang J, Zhang S (2011) Assessment of contrast-enhanced ultrasonography in diagnosis and preoperative localization of insulinoma. Eur J Radiol 80:675–680
39. Wiesli P, Brändle M, Schmid C, Krähenbühl L, Furrer J, Keller U, Spinas GA, Pfammatter T (2004) Selective arterial calcium stimulation and hepatic venous sampling in the evaluation of hyperinsulinemic hypoglycemia: potential and limitations. J Vasc Interv Radiol 15:1251–1256
40. Brown CK, Bartlett D, Doppman JL, Gorden P, Libutti SK, Fraker DL, Shawker TH, Skarulis MC, Alexander HR (1997) Intraarterial calcium stimulation and intraoperative

ultrasonography in the localization and resection of insulinomas. Surgery 122:1189–1194

41. Varma V, Tariciotti L, Coldham C, Taniere P, Buckels JA, Bramhall SR (2011) Preoperative localisation and surgical management of insulinomas: single centre experience. Dig Surg 28:63–73
42. Vezzosi D, Bennet A, Rochaix P, Courbon F, Selves J, Pradere B, Buscail L, Susini C, Caron P (2005) Octreotide in insulinoma patients: efficacy on hypoglycemia, relationships with Octreoscan scintigraphy and immunostaining with anti-sst2A and anti-sst5 antibodies. Eur J Endocrinol 152:757–767
43. Kumbasar B, Kamel IR, Tekes A, Eng J, Fishman EK, Wahl RL (2004) Imaging of neuroendocrine tumors: accuracy of helical CT versus SRS. Abdom Imaging 29:696–702
44. Reubi JC, Waser B (2003) Concomittant expression of several peptide receptors in neuroendocrine tumours: molecular basis for in vivo multireceptor tumour targeting. Eur J Nucl Med Mol Imaging 30:781–793
45. McAuley G, Delaney H, Colville J, Lyburn I, Worsley D, Govender P, Torreggiani WC (2005) Multimodality preoperative imaging of pancreatic insulinomas. Clin Radiol 60:1039–1050
46. Zimmer T, Stölzel U, Bäder M, Koppenhagen K, Hamm B, Buhr H, Riecken EO, Wiedenmann B (1996) Endoscopic ultrasonography and somatostatin receptor scintigraphy in the preoperative localisation of insulinomas and gastrinomas. Gut 39:562–568
47. Doppman JL, Chang R, Fraker DL, Norton AJ, Alexander HR, Miller DL, Collier E, Skarulis MC, Gorden P (1995) Localization of insulinomas to regions of the pancreas by intra-arterial stimulation with calcium. Ann Intern Med 123:269–273
48. Wong M, Ida SH, Zahiah M, Azmi KN (2007) Intraoperative ultrasound with palpation is still superior to intra-arterial calcium stimulation test in localising insulinoma. World J Surg 31:586–592
49. Norton JA, Sigel B, Baker AR, Ettinghausen SE, Shawker TH, Krudy AG, Doppman JL, Taylor SI, Gordon P (1985) Localization of an occult insulinoma by intraoperative ultrasonography. Surgery 97:381–384
50. Hayashi T, Honda H, Yasumori K, Kawashima A, Kaneko K, Fukuya T, Tateshi Y, Ro T, Matsuda K (1995) Nihon Igaku Hoshasen Gakkai Zasshi 55:952–956
