# Supplementary material for: Diagnostic value of ASVS for insulinoma localization: A systematic review and meta-analysis
Source: PLoS One. 2019 Nov 19;14(11):e0224928. doi: 10.1371/journal.pone.0224928 (PMC6863549; doi:10.1371/journal.pone.0224928)
Supplement: S2 File — (ZIP) [file pone.0224928.s002.zip › included studies/经动脉钙剂刺激试验术前定位诊断胰岛素瘤的价值.pdf]

·介入放射学·

经动脉钙剂刺激试验术前定位诊断胰岛素瘤的价值

金征宇　赵平　李晓光　潘杰　赵玉沛　蔡力行　钟守先

【摘要】 目的　评价经动脉钙剂刺激试验对胰岛素瘤术前定位的诊断作用。方法　对 16 例临床疑为胰岛素瘤而胰腺 B 超和 CT 检查均阴性的患者,术前应用经动脉钙剂刺激肝静脉取血测定胰岛素(ASVS)的方法进行肿瘤定位。经各供胰动脉分别注射葡萄糖酸钙,在注射前及注射后 30、60、90、120、150 s 分别取 2 ml 肝静脉血测定胰岛素。动脉刺激后血胰岛素水平的峰值超过基础值 2 倍以上,则确定肿瘤位于该动脉供应的胰腺区域。结果　16 例患者,手术和病理证实孤立性胰岛素瘤 12 例和胰岛细胞增生 1 例,除 1 例胰岛素瘤外,ASVS 对肿瘤均做出了准确定位;另 3 例经手术探查或随访排除胰岛素瘤,ASVS 结果亦为阴性。因此 ASVS 术前定位胰岛素瘤或胰岛细胞增生的敏感性为 92.3%(12/13),特异性为 100%(3/3),准确性为 93.7%(15/16)。结论　经动脉钙剂刺激肝静脉取血测定胰岛素的方法安全准确,适用于其他影像学检查阴性的胰岛素瘤的术前定位。

【关键词】 胰岛素瘤; 放射学,介入性; 诊断技术,内分泌; 评价研究

Value of the calcium intraarterial stimulation test in preoperative localization of insulinoma *JIN Zhengyu**, *ZHAO Ping*, *LI Xiaoguang*, *PAN Jie*, *ZHAO Yupei*, *CAI Lixing*, *ZHONG Shouxian*. * *Department of Radiology*, *Peking Union Medical College Hospital*, *Chinese Academy of Medical Sciences*, *Beijing* 100730, China

【Abstract】 **Objective** To evaluate the efficacy of the intraarterial calcium test in preoperative localization of insulinomas. **Methods** Sixteen patients with suspected insulinoma underwent ASVS method, which meant injecting calcium stimulant into pancreatic arteries and then taking blood specimens from hepatic vein to gauge the insulin levels. Six samples, each 2 ml, were taken before and 30, 60, 90, 120, 150 seconds after the injection. **Results** Twelve solitary insulinomas and one hyperplasia were diagnosed after surgery. Except one insulinoma, ASVS allowed accurate localization of the tumor. For the other three cases, which were excluded the diagnosis of insulinoma by surgical exploration or follow-up, ASVS were negative. Thus the sensitivity, specificity, and overall accuracy of ASVS were 92.3%(12/13), 100%(3/3), and 93.7%(15/16), respectively. **Conclusion** ASVS is an accurate and safe technique to localize insulinomas, especially for those undetectable at preoperative morphologic studies.

【Key words】 Insulinoma; Radiology, interventional; Diagnostic techniques, endocrine; Evaluation studies

胰岛素瘤是最常见的功能性胰岛细胞瘤,90%属良性,手术切除可以治愈,故术前对肿瘤进行准确定位是手术成功的关键[1]。但由于大多数的胰岛素瘤均较小,不引起胰腺大小形态的改变,普通的影像学检查手段如 B 超、CT 或 MRI 均难以发现。钙剂是胰岛素分泌的刺激物,而在胰岛素瘤或胰岛细胞增生时,正常 B 细胞功能受到抑制,只有肿瘤或增生细胞能够对钙剂做出反应。向供应胰腺不同区域的动脉内注入钙剂,而后在肝静脉内取血测定胰岛素水平,如与基础值相比明显升高,就可推断出肿瘤所在的部位。此方法称为经动脉钙剂刺激肝静脉取血测定胰岛素(arterial calcium stimulation with hepatic venous sampling,ASVS),其定位准确性不依赖于瘤体的大小,因此对胰岛素瘤,特别是对影像学检查阴性的小胰岛素瘤术前定位准确性很高[1,2]。笔者所在单位自 1995 年起应用此项技术,取得了满意的效果,现报道如下。

材料与方法

一、研究对象

自 1995 年 9 月至 2000 年 2 月,共对 16 例临床

作者单位:100730 中国医学科学院 中国协和医科大学 北京协和医院放射科(金征宇、李晓光、潘杰),外科(赵平、赵玉沛、蔡力行、钟守先)

万方数据

疑为胰岛素瘤而胰腺 B 超和 CT 检查均阴性的患者进行了 ASVS 检查，其中男 6 例，女 10 例，年龄 9 ~ 63 岁。所有患者均排除了其他原因引起的高胰岛素血症。16 例患者空腹血糖(0.44 ~ 2.70 mmol/L)均明显低于正常。其中 13 例具有典型的 Whipple 三联征，即空腹时发作，发作时血糖低于 2.80 mmol/L，以及升高血糖后症状迅速缓解；另外 3 例患者 Whipple 三联征阴性。Whipple 三联征阳性的 13 例患者中，3 例曾行剖腹手术，其中 2 例剖腹探查未找到肿瘤，1 例为胰岛素瘤切除术后症状复发。

二、ASVS 的方法

在数字减影血管造影(DSA)引导下，按 Seldinger 方法首先经左股静脉插管至右肝静脉并保留，然后经右股动脉分别插管至胃十二指肠动脉(GDA)、脾动脉远侧(脾门处)(SA/D)、脾动脉近侧(SA/P)、肠系膜上动脉(SMA)及肝固有动脉(HA)。每插入一动脉先行血管造影并确定导管的准确位置，再将葡萄糖酸钙按 Ca^{2+} 0.010 ~ 0.025 mEq/kg 的剂量用生理盐水稀释成5 ml，1 次匀速注入。在注射前和注药后 30、60、90、120 和 150 s 分别抽取肝静脉血 2 ml，所有血标本均在 -20℃环境下储存备测，采用放射免疫法(以下简称放免)测定血胰岛素含量。每次动脉注射钙剂间隙不少于 15 min，以避免钙再循环的影响。注射钙剂后，若静脉血胰岛素水平超过注射前基础值的 2 倍，则提示分泌胰岛素的肿瘤位于该动脉供应的区域内。

结　果

16 例患者的 ASVS 结果与手术及随访对照见表 1。

13 例 Whipple 三联征阳性患者，均行手术探查，病理证实胰岛素瘤 12 例，β 胰岛细胞增生 1 例。

12 例胰岛素瘤均为单发，其中 1 例为原胰岛素瘤术后肝转移。肿瘤位于胰头钩突部 7 例，胰体部 2 例，胰尾部 2 例，右肝转移灶 1 例。对其中 11 例患者，ASVS 结果为阳性，且定位诊断与手术结果完全一致(图 1)。对 1 例位于胰腺钩突部的胰岛素瘤，血管造影结果阳性，但 ASVS 结果不能明确(例 5)。

3 例再手术患者，血管造影均为阴性，ASVS 检查，其中 2 例分别在胃十二指肠动脉和肠系膜上动脉行钙剂刺激后，肝静脉血胰岛素水平超过刺激前 5 倍和 2 倍，从而将肿瘤定位于胰头钩突部。另1 例在肝固有动脉行钙剂刺激后有胰岛素分泌高峰，术中发现右肝有一结节，病理证实为胰岛素瘤肝脏转移。

万方数据

表 1　16例患者概况、ASVS 定位、手术与随访结果

例号	性别	年龄(岁)	Whipple 三联征	SAG	ASVS GDA	ASVS SMA	ASVS SA/D	ASVS SA/P	ASVS HA	手术定位或随访结果
1	女	9	+	+	+	+	-	-	-	胰头钩突部
2	男	31	+	+	+	-	-	-	-	胰头钩突部
3	女	33	+	+	-	-	-	+	-	胰体
4	男	38	-	-	-	-	-	-	-	-
5	女	63	+	+	+	+	-	+	-	胰钩突部
6	女	52	-	-	-	-	-	-	-	-
7	男	12	+	+	+	-	-	-	-	胰头
8	女	34	+	+	-	-	+	-	-	胰尾
9	女	42	+	+	+	-	-	-	-	胰头
10	男	37	+	-	+	-	-	-	+	胰头钩突部
11	女	57	+	-	-	-	-	-	+	右肝
12	女	37	-	-	-	-	-	-	-	-
13	男	26	+	+	-	-	+	-	-	胰尾
14	女	46	+	+	-	-	-	+	-	胰体
15	女	33	+	-	+	-	-	-	-	胰头钩突部
16	男	20	+	-	-	-	±	±	-	胰体尾增生

注：+ 为阳性；- 为阴性；± 代表胰岛素水平较刺激前明显升高，但不足 2 倍

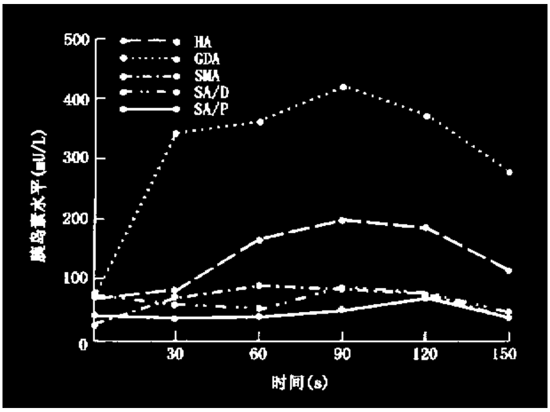

图 1　例10肝动脉和胃十二指肠动脉钙剂刺激后，肝静脉血胰岛素水平升高分别超过刺激前的 4 倍和 7 倍，手术示胰头钩突部胰岛素瘤

对 β 胰岛细胞增生患者(例 16)，血管造影无异常发现，ASVS 结果在脾动脉远近段刺激后肝静脉血胰岛素均有明显升高，但不足刺激前的 2 倍(图 2)。手术探查发现胰体尾局部增粗，但未扪及肿物，胰头部未见异常，故行胰体尾切除术，术后病理示胰岛细胞轻度增生(较正常值高近 4 倍)。

3 例 Whipple 三联征阴性患者，血管造影和 ASVS 结果均为阴性，故临床否定胰岛素瘤的诊断。其中 1 例坚持要求手术探查，术中未能找到肿瘤，随访 1 年低血糖症状未再发生。另 2 例未行手术，随访 3 年，多次行 B 超和 CT 复查仍未发现胰岛素瘤。

以手术病理及随访结果为参照标准，ASVS 术前

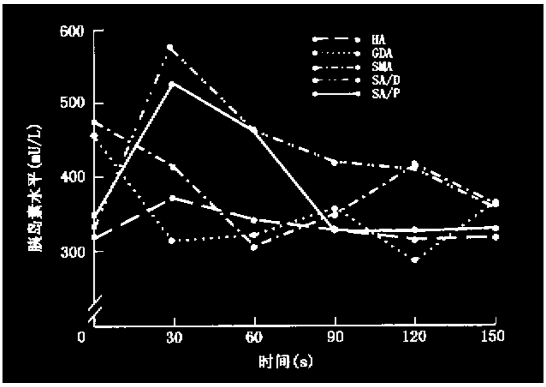

图 2　例16胰岛细胞增生患者,在脾动脉远近段刺激后肝静脉血胰岛素均有明显升高,但不足刺激前的 2 倍

定位胰岛素瘤或胰岛细胞增生的敏感性为 92.3%(12/13),特异性为 100%(3/3),准确性为 93.7%(15/16)。

在 ASVS 检查过程中及检查后患者均未发生明显的不良反应。

讨　论

根据典型的 Whipple 三联征和血浆胰岛素水平的升高,胰岛素瘤的诊断并不困难,但其术前定位诊断一直是外科医师和影像学家所关心的问题。尽管多数胰岛素瘤可在术中扪及而发现,但需要对胰腺进行充分游离,损伤较大,而且需要术者具有丰富的经验;即使如此,仍有 10%的小腺瘤术中不能扪及,因此术前对胰岛素瘤进行准确定位就十分必要[3]。

一、胰岛素瘤术前定位诊断方法的比较

目前用于胰岛素瘤术前定位诊断的方法很多,可分为形态学定位和功能性定位 2 大类。由于大多数的胰岛素瘤直径小于 2 cm,普通 B 超、CT 和 MRI 均难以发现,阳性率一般在 30%左右[4]。有报道内镜超声或术中超声对胰岛素瘤的定位准确率较高[5,6],可达 80%～100%,但这些方法技术复杂,要求操作者具有丰富的经验,临床推广比较困难。胰岛素瘤多数血供丰富,与普通 B 超、CT 和 MRI 相比,选择性动脉造影(SAG)的阳性率较高,一般在 50%～60%,最高可达 80%,但其对诊断胰岛细胞增生无能为力。鉴于术前形态学检查方法对于胰岛素瘤定位不理想,功能性试验如经皮经肝门静脉取血测定胰岛素(percutaneous transhepatic portal vein catheteration, PTPC)多年来被认为是最终的定位方法,其优势在于定位准确性不依赖于瘤体的大小,对于影像学检查阴性的所谓隐匿性胰岛素瘤或胰岛细胞增生的定位准确性很高。但 PTPC 法侵袭性大,技术要求高,有一定的痛苦和并发症,在一定程度上准确性也受回流静脉变异的影响,因此未能在临床上得到推广[2,7]。

1991 年 Doppman 等[8]首先报道用 ASVS 方法成功地对 4 例胰岛素瘤患者做出了术前准确定位,此后,其效果陆续被更多的病例所证实。最初根据血供将胰腺分为头颈和体尾 2 个区域,以后又将脾动脉注射分为远侧和近侧,先远后近分别刺激,以辨别体、尾部肿瘤。研究表明:从右肝静脉或左肝静脉采集血样测定胰岛素浓度并无明显差异。Doppman 等以注射钙剂后 30、90 s 血胰岛素水平升高 2 倍以上区域为阳性定位结果。1995 年 Doppman 等[9]报道 25 例胰岛素瘤患者的 ASVS 检查结果,敏感性为 88%(22/25),同组中 PTPC 敏感性为 67%(6/9),SAG 38%(9/25),MRI 41%(9/21),CT 17%(4/23),B 超 9%(2/22)。肿瘤直径为 6～25 mm(平均 15 mm)。由此可见,ASVS 定位胰岛素瘤的敏感性明显高于其他各种方法。本组 13 例胰岛素瘤(包括 1 例胰岛细胞增生),ASVS 对其中 12 例做出了准确定位,敏感性为 92.3%;尤其是 3 例再次手术患者,B 超、CT 以及血管造影均未能发现病灶,ASVS 定位结果与手术完全一致。

二、ASVS 结果判读及技术探讨

一般而言,ASVS 的结果是易于判读的。典型的胰岛素瘤表现为在相应动脉内刺激后 30 或 60 s 出现胰岛素高峰,达基础值的 2 倍以上,120 s 后回到基线水平。胃十二指肠动脉和(或)肠系膜上动脉刺激后阳性提示肿瘤位于胰头钩突部,脾动脉近端阳性提示胰体部,脾动脉远端阳性提示胰尾部,胰头钩突部肿瘤还可出现肝固有动脉刺激后高峰,单独的肝固有动脉阳性十分罕见,本组 1 例证实为位于右肝的转移灶。如果在供应胰腺不同解剖区域的动脉进行刺激后均出现阳性则不能对肿瘤进行定位,本组例 5 就是这种情况。出现这种情况的原因我们推测是由于血管变异或动脉插管后持续痉挛,钙剂返流入其他动脉,从而同时刺激了胰腺的不同区域所致。另外,有 10%左右的胰岛素瘤为多发,理论上也可出现这种情况,实际工作中笔者尚未遇到。

在作 ASVS 时,必须注意以下几个要点:(1)钙剂刺激前,选择性动脉造影是必要的,一方面可发现血管变异,另一方面与 ASVS 结果相互验证补充,对胰岛素瘤的定位准确性更高。如在造影时发现血管痉挛,可使用解痉剂。(2)造影后最好等待几分钟再

万方数据

行 ASVS,以避免对比剂对胰岛素放免测定的可能影响;每次动脉注射钙剂间隔不少于 15 min,以避免钙再循环的影响。(3)取血后,要立即冰冻保存,及时测定。

三、胰岛细胞增生的术前定位

对于胰岛细胞增生,任何形态学方法均不能定位,术中扪诊也难以发现,而 ASVS 可能对其定位有助。Pereira 等[1]曾报道 1 例位于胰头部的结节样增生,在肠系膜上动脉和胃十二指肠动脉行钙剂刺激后,血胰岛素水平分别升高 2 倍和 7 倍,但呈持续性升高,反应曲线不同于典型的胰岛素瘤。本组 1 例胰岛细胞轻度增生,位于胰体尾部,在脾动脉远侧和近侧行钙剂刺激后出现胰岛素分泌高峰,但均不到基础值的 2 倍,考虑与增生程度有关。由于病例数太少,ASVS 对胰岛细胞增生的定位诊断价值及其反应曲线尚需进一步研究。

总之,ASVS 方法是选择性血管造影技术的延伸,可以同时进行,不需额外复杂的操作;2 者结合,可以互相验证和补充,对胰岛素瘤和胰岛细胞增生的术前定位诊断准确性很高。本研究结果表明,ASVS 检查方法主要适用于临床诊断胰岛素瘤而影像学检查阴性的患者,以及前次手术探查阴性拟再手术的患者。

参 考 文 献

1 Pereira PL, Roche AJ, Maier GW, et al. Insulinoma and islet cell hyperplasia: value of the calcium intraarterial stimulation test when findings of other preoperative studies are negative. Radiology, 1998, 206:703-709.

2 赵平,金征宇,胡麦,等. 动脉钙剂刺激静脉采血测胰岛素定位诊断胰岛素瘤. 中华医学杂志,1998,78:192-194.

3 Bottger TC, Junginger T. Is preoperative radiographic localization of islet cell tumors in patients with insulinoma necessary? World J Surg, 1993, 17:427-432.

4 Comi RJ, Garden P, Doppman JL. Insulinoma. In: Vay Liang W, ed. The pancrease: biology pathobiology and disease. 2 nd ed. New York: Raven Press Ltd, 1993. 979-996.

5 Palazzo L, Roseau G, Salmeron M. Endoscopic ultrasonography in the preoperative localization of pancreatic endocrine tumors. Endoscopy, 1992, 24:350-353.

6 Van Heerden JA, Grant CS, Czako PF, et al. Occult functioning insulinomas: which localizing studies are indicated? Surgery, 1992, 112:1010-1015.

7 Vinik AI, Delbridge L, Moattari R, et al. Transhepatic portal vein catheterization for localization of insulinomas: a ten-year experience. Surgery, 1991, 109:1-11.

8 Doppman JL, Miller DL, Chang R, et al. Insulinomas: localzation with selective intraarterial injection of calcium. Radiology, 1991, 178:237-241.

9 Doppman JL, Chang R, Fraker DL, et al. Localization of insulinomas to regions of the pancreas by intra-arterial stimulation with calcium. Ann Intern Med, 1995, 123:269-273.

(收稿日期:2001-01-31)
(本文编辑:史红)

·读者·作者·编者·

读者来函及作者复函

编辑同志:

贵刊第 35 卷第 10 期第 755～758 页《多层面螺旋 CT 三维血管造影诊断脑动脉瘤临床价值的初步探讨》一文中,"材料与方法"段第 7 行"……数据采集层厚 1.0 mm,螺距 3.0/50 mm;……"与"讨论"段第 756 页右栏倒数第 2 行"……以 0.5 mm 层厚采集原始数据,……"在层厚上不相符合,且螺距是床的转动速度与层厚的比值,不宜用"3.0/5.0 mm"表示。特此指出,盼复!

此致

敬礼

读者:王亚非

2001.11.5

编辑同志:

贵刊转来的读者来信收到。经认真查阅文稿的底稿和已出版的杂志后,发现读者来信中指出的问题确实存在。数据采集层厚应统一为"1.0 mm";螺距应表示为"3.0/5.0"。对于本人撰稿时的笔误和核校样稿时的失误向广大读者深表歉意,并希望通过编辑部对王亚非同志表示衷心的感谢!

此致

敬礼

作者:朱玉森

2001.11.26

万方数据
