# Supplementary material for: Diagnostic value of ASVS for insulinoma localization: A systematic review and meta-analysis
Source: PLoS One. 2019 Nov 19;14(11):e0224928. doi: 10.1371/journal.pone.0224928 (PMC6863549; doi:10.1371/journal.pone.0224928)
Supplement: Attachment — Submitted filename: response letter.docx [file pone.0224928.s004.docx]

responds to academic editor

1. We have deposited my laboratory protocols in protocols.io. We get the DOI: dx.doi.org/10.17504/ protocols.io.7imhkc6.

2. We have no change to my financial disclosure.

3. We have upload the revision of my manuscript according to your direction.

Responds to the reviewers:

1. Thank you. We have addressed reviewers’ comments raised in a previous round of review.
2. The manuscript was written in accordance with normal criteria of standard diagnostic meta analysis. So We think it is technically sound, and the data do support the conclusions.
3. The manuscript comply with the Standard Writing of Meta-analysis. And We think statistical analysis been performed appropriately and rigorously.
4. All data underlying the findings described in the manuscript fully available without restriction. We already deposited the meta analysis and the relevant data and step to a public repository. We got the DOI: dx.doi.org/10.17504/ protocols.io.7imhkc6.
5. After we have finished the paper, we invited several mother tongue editor to polish the language.
6. Dear teacher,Thank you very much for your instruction. We also think that the wide range of expertise in the use of this investigation which would have significant implications on the results. For the role of GLP 1 receptor imaging in the localization of insulinoma, we also searched a lot of literature, and made a more detailed discussion.
7. Thanks for the peer review. We learnt a lot.
